# Advancing Parkinson’s Disease Diagnostics: The Potential of Arylpyrazolethiazole Derivatives for Imaging α-Synuclein Aggregates

**DOI:** 10.1021/acsomega.4c01301

**Published:** 2024-05-30

**Authors:** Federica Bonanno, Ran Sing Saw, Daniel Bleher, Ioannis Papadopoulos, Gregory D. Bowden, Kaare Bjerregaard-Andersen, Albert D. Windhorst, Bernd J. Pichler, Kristina Herfert, Andreas Maurer

**Affiliations:** † Werner Siemens Imaging Center, Department of Preclinical Imaging and Radiopharmacy, Eberhard Karls University Tübingen, Röntgenweg 13, Tübingen 72076, Germany; ‡ Cluster of Excellence iFIT (EXC 2180) “Image-Guided and Functionally Instructed Tumor Therapies”, Eberhard Karls University Tübingen, Röntgenweg 11, Tübingen 72076, Germany; § Department of Antibody Engineering and Biochemistry, H. Lundbeck A/S, Ottiliavej 9, Valby 2500, Denmark; ∥ Department of Radiology and Nuclear Medicine, Amsterdam UMC, Vrije Universiteit Amsterdam, De Boelelaan 1085c, 1081 HV Amsterdam, The Netherlands

## Abstract

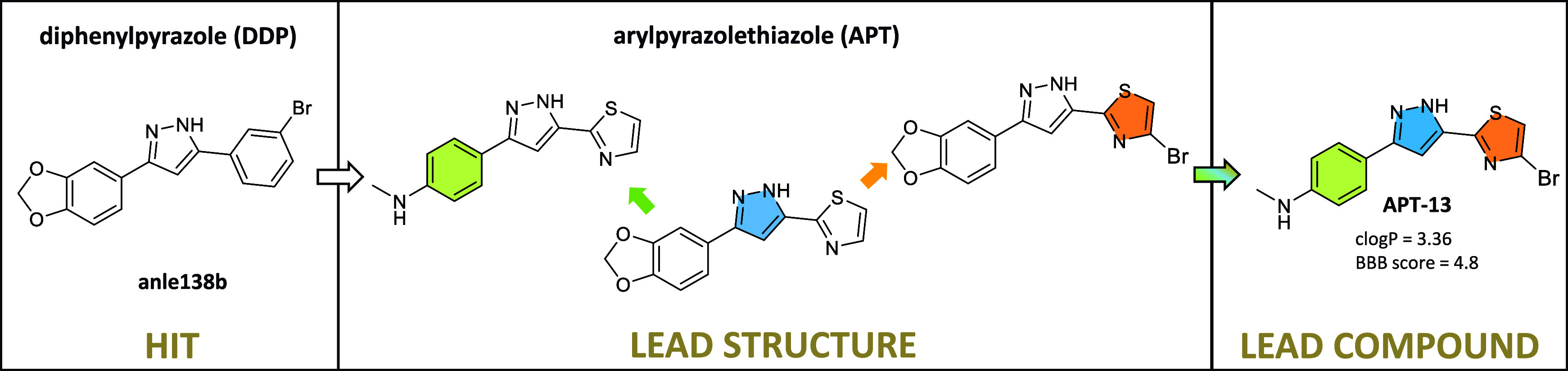

The development of positron emission tomography (PET) tracers capable of detecting α-synuclein (α-syn) aggregates in vivo would represent a breakthrough for advancing the understanding and enabling the early diagnosis of Parkinson’s disease and related disorders. It also holds the potential to assess the efficacy of therapeutic interventions. However, this remains challenging due to different structures of α-syn aggregates, the need for selectivity over other structurally similar amyloid proteins, like amyloid-β (Aβ), which frequently coexist with α-syn pathology, and the low abundance of the target in the brain that requires the development of a high-affinity ligand. To develop a successful PET tracer for the central nervous system (CNS), stringent criteria in terms of polarity and molecular size must also be considered, as the tracer must penetrate the blood–brain barrier and have low nonspecific binding to brain tissue. Here, we report a series of arylpyrazolethiazole (APT) derivatives, rationally designed from a structure–activity relationship study centered on existing ligands for α-syn fibrils, with a particular focus on the selectivity toward α-syn fibrils and control of physicochemical properties suitable for a CNS PET tracer. In vitro competition binding assays performed against [^3^H]MODAG-001 using recombinant α-syn and Aβ_1–42_ fibrils revealed **APT-13** with an inhibition constant of 27.8 ± 9.7 nM and a selectivity of more than 3.3 fold over Aβ. Radiolabeled [^11^C]**APT-13** demonstrated excellent brain penetration in healthy mice with a peak standardized uptake value of 1.94 ± 0.29 and fast washout from the brain (*t*_1/2_ = 9 ± 1 min). This study highlights the potential of **APT-13** as a lead compound for developing PET tracers to detect α-syn aggregates in vivo.

## Introduction

Misfolded α-synuclein (α-syn) is a major component of Lewy bodies and Lewy neurites, pathological aggregates that accumulate in the brains of individuals with Parkinson’s disease (PD), and other α-synucleinopathies, such as multiple system atrophy (MSA) and dementia with Lewy bodies (DLB).^1^ PD is one of the most common neurodegenerative disorders characterized by the loss of dopaminergic neurons in the substantia nigra, resulting in reduced dopamine levels in the brain, which causes typical PD-related motor symptoms, such as bradykinesia, rigidity, tremor, and postural instability.^2^ Although these symptoms typically become clinically evident during the intermediate stages of the disease, the underlying pathology likely begins years earlier in the olfactory bulb and lower brainstem.^3^ The protein accumulation progressively spreads to other central nervous system (CNS) regions in a self-promoting pathological process, causing the disease to progress.^4^ Currently, α-syn inclusions in the brain can only be detected through histological examination of post-mortem brain tissue or inferred by measuring misfolded α-syn protein levels in cerebrospinal fluid.^5^ Thus, the possibility of detecting and quantifying α-syn deposition in vivo in the brain would represent a breakthrough achievement for the field. Positron emission tomography (PET) is a noninvasive imaging technique that uses radioactive ligands to localize and quantify specific targets in vivo with high sensitivity. Such a method can enable the detection of α-syn aggregates with accuracy when a suitable radioligand is utilized. This makes PET a valuable tool for the study of disease progression and novel drug development, as well as for early and accurate diagnosis in patients.

Diagnosing the disease at the earliest possible stage is crucial, as early intervention will be essential to limiting the neurodegenerative progression when therapies become available.

**Figure 1:**
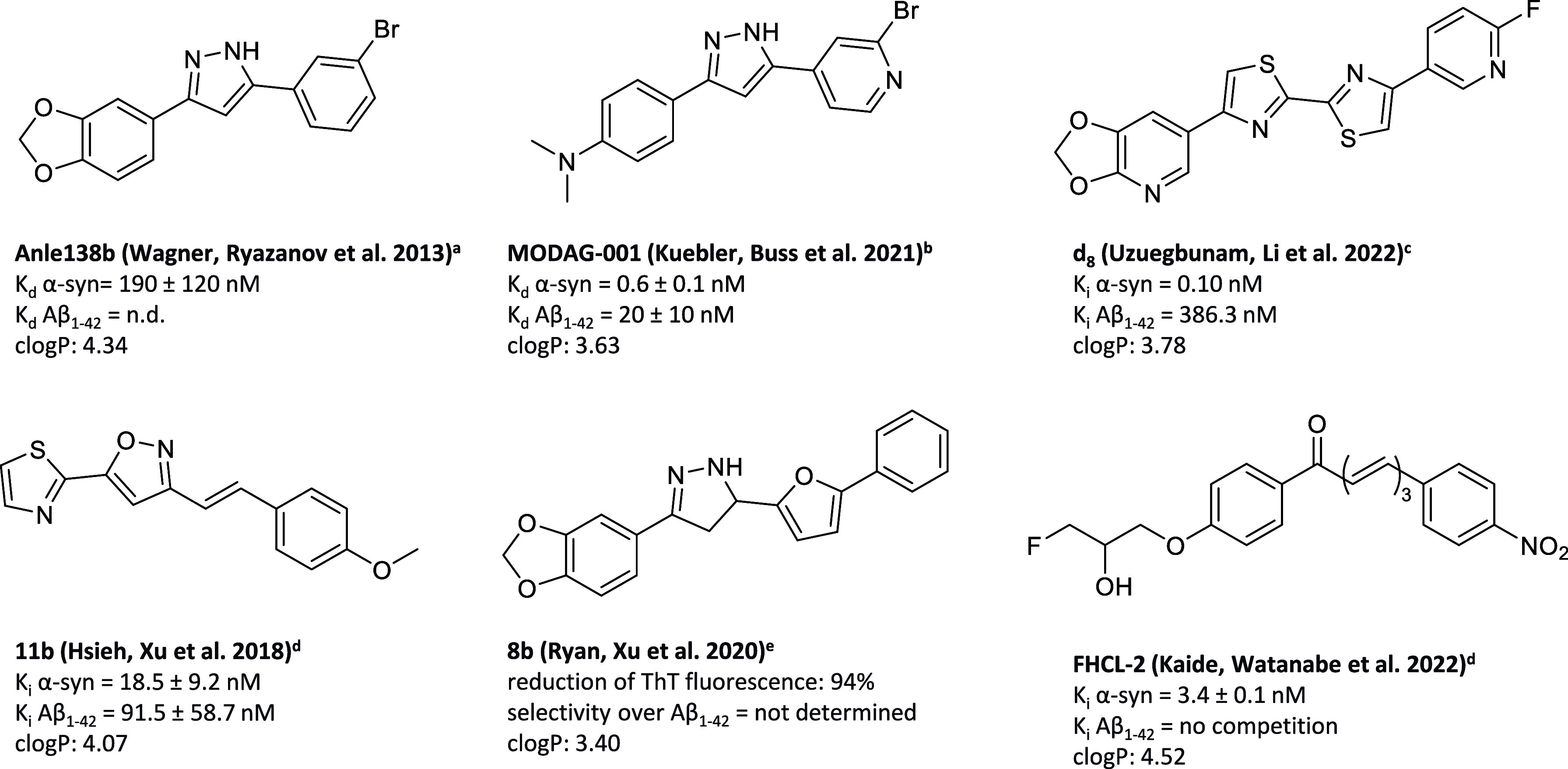
Ligands that have been reported to bind to α-synuclein aggregates. Results obtained by ^a^fluorescence spectroscopy; ^b^in vitro saturation binding assays using recombinant fibrils; ^c^in vitro competition binding assay against [^3^H]DCVJ; ^d^in vitro thioflavin competition binding assay; ^e^in vitro thioflavin fluorescence assay; *c*log*P* calculated by molinspiration. (n.d. = not determined).

Developing a PET tracer for α-syn aggregates faces several challenges. Considering the density of binding sites in the target pathology, aggregated α-syn is low in abundance, necessitating a high-affinity ligand, most likely in the subnanomolar range, that is also selective for α-syn relative to amyloid-beta (Aβ) and tau aggregates, which are often colocalized and structurally similar.^5^ Since most of α-syn is localized intracellularly, a successful PET tracer would have to cross the cell membrane to engage its target in addition to the blood–brain barrier (BBB) through passive diffusion.^5^ The BBB penetrance should be high, with a recommended standard uptake value (SUV) above 1.0 within a few minutes.^6^ A low signal-to-noise ratio in the brain interferes with quantification and can hinder the imaging of pathological α-syn. Therefore, the tracer should have a low nondisplaceable binding (NDB).^6^ As a “rule of thumb”, candidate CNS PET agents should be moderately lipophilic (log*D*_7.4_ = 1–3)^7^ and relatively small (<500 Da)^8,9^ to achieve the desired outcome parameters. Although several small molecules have been identified that bind to α-syn fibrils^10^ (Figure 1), so far, none have been translated to clinical routine as a result of poor selectivity toward α-syn or inadequate pharmacokinetic profiles.

Recently, significant advancements have been made in detecting pathological α-syn deposits. The ligand [^18^F]ACI-12589 demonstrated promising results, showing high affinity and specificity for α-syn aggregates in in vitro autoradiography (AR) and homogenate binding assays of MSA and PD tissues. Clinical evaluations revealed its effective binding in MSA patients in regions associated with α-syn pathology, although it did not bind in PD patients.^11^ However, its off-target binding to monoamine oxidase-B (MAO-B), as indicated by preblocking experiments with selegiline, highlights a limitation. Another ligand, [^18^F]F0502B, exhibited good binding affinity in the single-digit nanomolar range to human brain lysates and high selectivity against Aβ fibrils in vitro. This ligand retained in the brain of macaques injected with α-syn preformed fibrils and adeno-associated virus α-syn A53T.^12^ However, the absence of a quantitative kinetic and metabolic analysis of [^18^F]F0502B limits its current understanding. Furthermore, [^11^C]MODAG-005 has recently been published, but to date it has only been evaluated on one patient.^13^

Given these findings, there is a pressing need to explore further and develop new tracer candidates that meet all the requirements for a CNS PET tracer suitable for clinical applications. Here, we report the design, synthesis, in vitro and in vivo evaluation of a series of low molecular weight arylpyrazolethiazole (APT) derivatives with optimized lipophilicity. This structural class demonstrates nanomolar range binding affinities and high selectivity for α-syn over Aβ fibrils (3.3 to 20.7-fold), underscoring their potential of effective CNS PET tracers.

## Results and Discussion

### Molecular Design of the APT Library

A common feature of the reported α-syn ligands is the presence of a multiple conjugated aromatic rings system: they can consist of an aromatic ring, which is generally a varied substituted benzene ring, with or without heteroatoms; a central core, usually a chalcone system,^14^ or a heterocycle;^15−17^ and a second aromatic ring, such as a benzene ring or heterocycle (Figure 1).

Anle138b, the first described diphenylpyrazole (DPP) derivative,^15^ is a promising drug candidate against α-synucleinopathies due to its ability to inhibit the aggregation of α-syn oligomers. However, it has unfavorable properties as a PET tracer due to its high lipophilicity (calculated log*P* for anle138b = 4.34).

Moreover, [^11^C]MODAG-001,^16^ a recently developed derivative of anle138b^15^ with enormous potential as a very high-affinity ligand for α-syn fibrils and excellent ability to penetrate the mouse brain, showed no detectable binding in in vitro AR in brain sections of Lewy body dementia (LBD), possibly due to high nonspecific binding (NSB), as well as concomitant moderate binding affinity to Aβ fibrils (reported *K*_d_ for Aβ_1–42_ = 20 ± 10 nM) in Alzheimer’s disease (AD) brain tissue. Both high lipophilicity and low selectivity over other amyloid protein aggregates are well-known hurdles against the development of imaging agents. Nevertheless, the DPP class of compounds provides a promising template for further structure–activity relationship (SAR)-based research of small molecule ligands for α-syn aggregates. Recently, it has been shown that the pyrazole ring in anle138b^15^ significantly contributes to its binding affinity. Antonschmidt and colleagues elucidated a possible binding mode of anle138b using nuclear magnetic resonance (NMR) spectroscopy studies.^18^ Anle138b establishes stable polar interactions with the backbone moieties inside a tubular cavity of the fibrils either through hydrogen bonds via the pyrazole ring or halogen bonds involving the bromine atom.^18^

Recently, Uzuegbunam et al. identified a class of potent α-syn ligands consisting of bisthiazole-based analogs, pointing out that the inclusion of a methylenedioxy functional group in the structure significantly enhances binding affinity and selectivity for α-syn fibrils.^17^ A 1,3-benzodioxole moiety is also recurrent in other known ligands with high affinity for α-syn aggregates, including anle138b.^15,17,19^ Furthermore, in known classes of compounds, a thiazole ring is reported for some selective ligands for α-syn over Aβ.^17,20^

**Figure 2:**
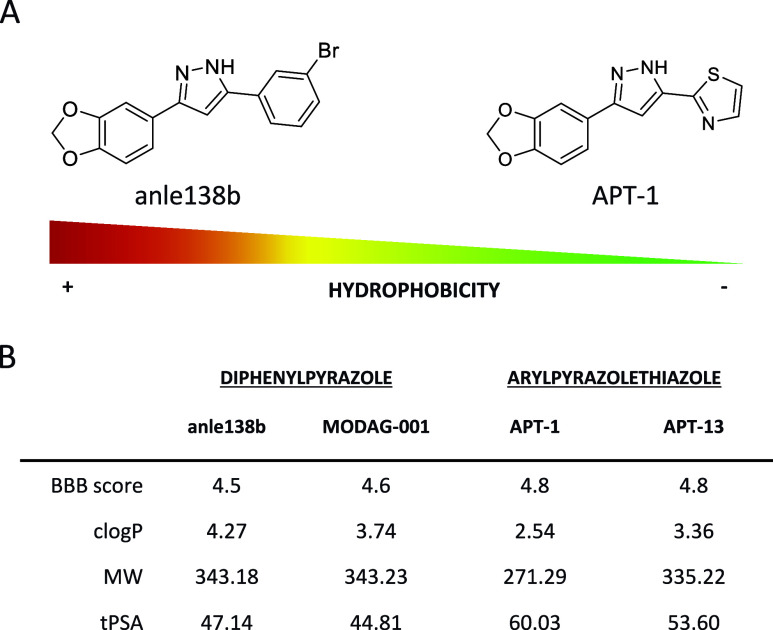
(A) Structure of anle138b and **APT-1**. (B) Properties of some derivatives of the APT class of compounds; *c*log*P*, MW, and tPSA calculated with Chemicalize software; BBB score calculated with the Excel sheet provided in the literature.^21^*c*log*P* = calculated log*P*; MW = molecular weight; tPSA = topological polar surface area.

In this study, we designed a class of APT compounds in which a thiazole ring replaces a benzene ring of the DPP analogs. This substitution significantly decreased lipophilicity, as exemplified by the comparison between anle138b and **APT-1** (Figure 2). **APT-1** was designed as the starting scaffold for all further SAR studies.

Figure 2 illustrates that the APT class of compounds exhibit promising calculated physicochemical properties, rendering them suitable for the development of a CNS PET tracers. We evaluated properties, including lipophilicity, topological polar surface area (tPSA), and a computational BBB permeability score.^22^ The BBB score is an algorithm proposed for predicting BBB penetration, in which molecules with a BBB score above 4 (with 6 being the highest) are more likely to penetrate the brain. All APTs have a calculated BBB score greater than 4.

**Figure 3:**
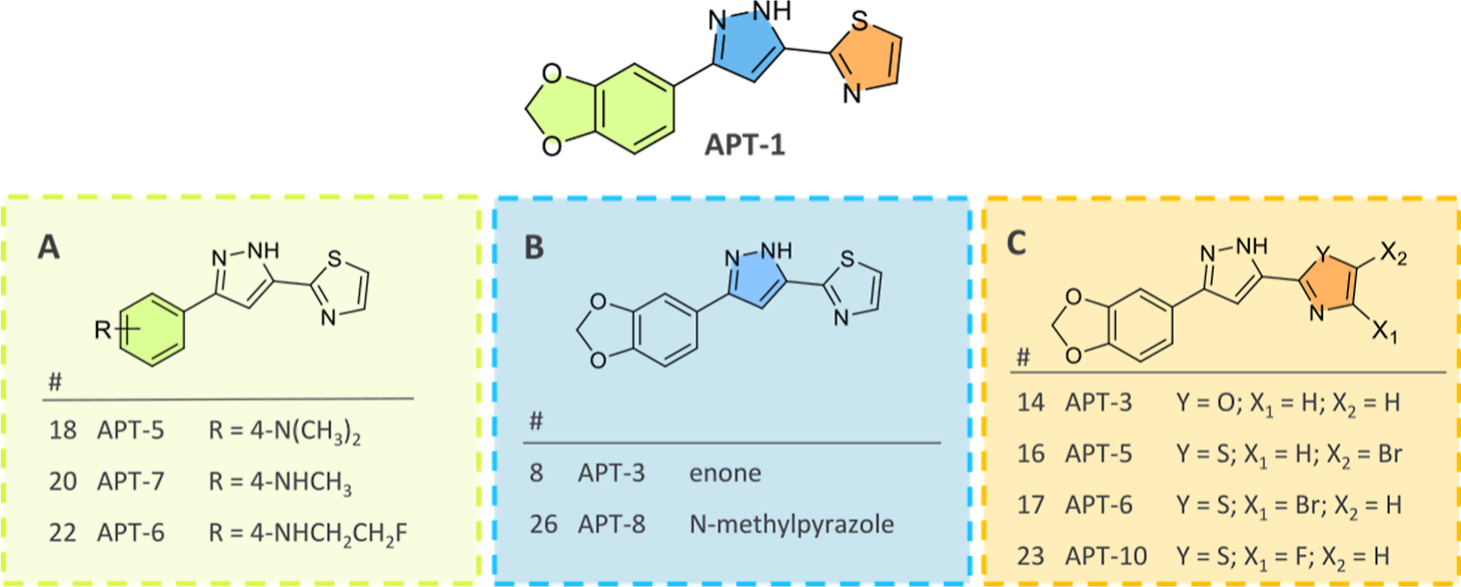
Modification of the **APT-1** structure.

Further modifications of **APT-1** targeted three parts of the molecule:

(A) The 1,3-dioxole was replaced in the *para* position to give the derivatives *N*,*N*-dimethylaniline, *N*-methylaniline, and *N*-(2-fluoroethyl)aniline. We assumed the significance of an electro-donating group in the *para* position to strengthen the hydrogen (H)-bond on the pyrazole ring and, in the case of the secondary amine, along with adding an electron bond donor. The inclusion of an *N*,*N*-dimethylaniline in MODAG-001 also proved beneficial^16^ (Figure 3A).
(B) Considering that pyrazoles can act as H-bond acceptors and donors with the amide hydrogens and carbonyl oxygens of a peptide backbone, the relevance of an H-bond donor (HBD) was investigated. To prove this, the pyrazole ring was replaced with a chalcone moiety as a bridging system. The *N*-methylpyrazole derivative was also included to further verify the observation that an HBD on the pyrazole improves binding to the pocket (Figure 3B).
(C) The thiazole ring was further substituted with halogens to favor hydrophobic interactions at the site (Figure 3C).

The direct comparison of the results obtained from modifying the three rings of **APT-1** led to the identification of **APT-13** as a lead compound. **APT-13** exhibits the highest affinity for α-syn (*K*_i_ = 27.8 ± 9.7 nM) of the series with good selectivity over Aβ while also possessing favorable pharmacokinetic properties as a PET tracer (Figure 7). To further verify the significance of the pyrazole ring for high affinity, chalcone analog derivatives **APT-11**, **APT-12**, and **APT-14** were also synthesized.

### Chemical Synthesis

**Scheme 1:**
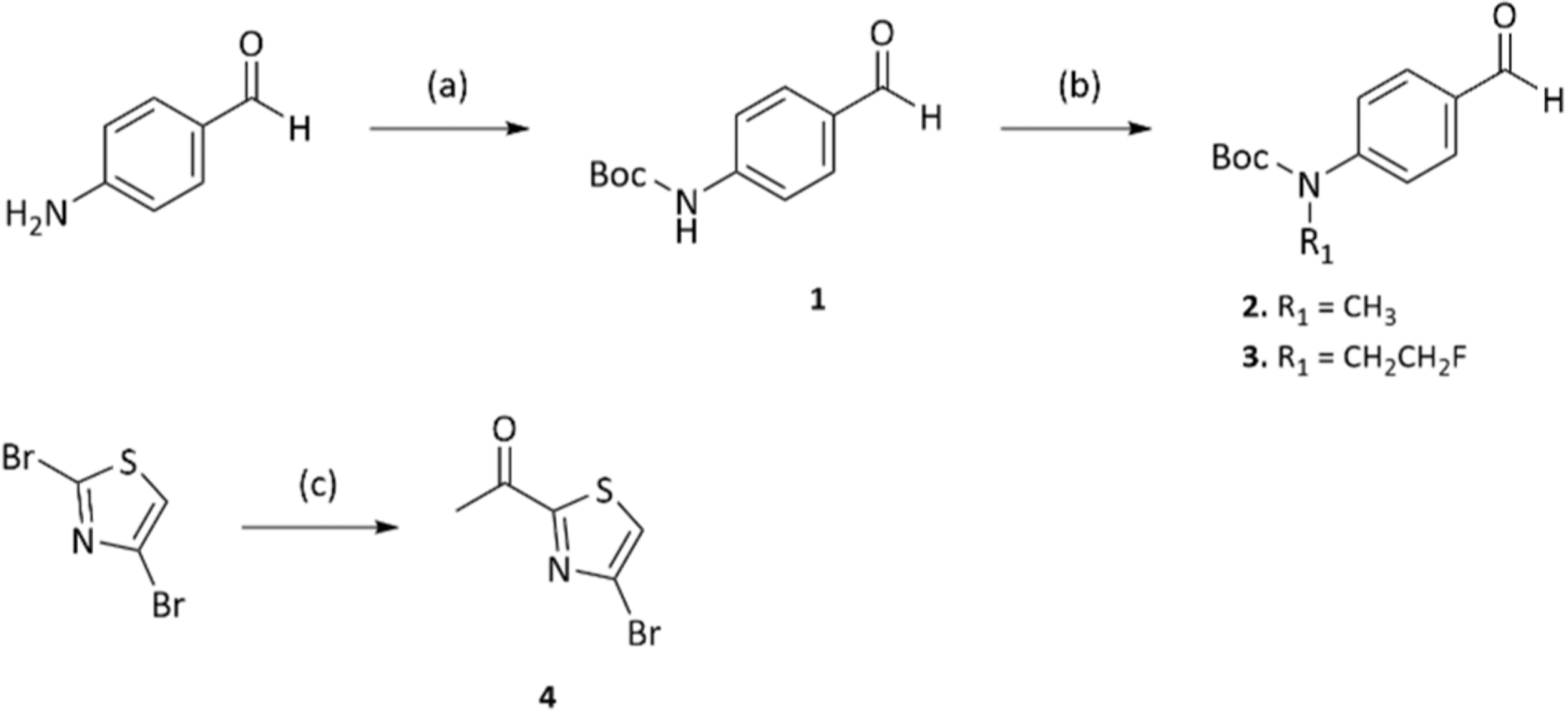
Synthesis of Intermediates **1–4** Reagents and conditions: (a) Boc-anhydride, THF, 18 h reflux, 48%; (b) NaH, MeI or 1-fluoro-2-iodoethane, *N*,*N*-dimethylformamide (DMF), 15 h, r.t., 59–72%; (c) *n*-BuLi, *N*-acetyl morpholine, THF, 3 h, −78 °C, 62%

**Scheme 2:**
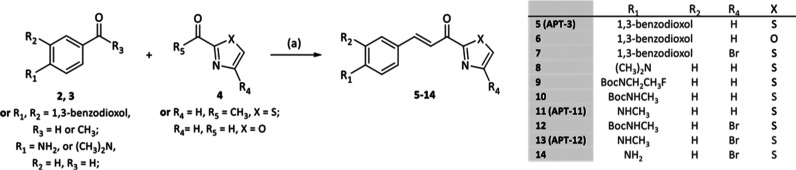
General Synthetic Route A Reagents and conditions: (a) piperidine, EtOH, reflux, 3 h, 46–94%; (a.2) HCl, 1.5–3 h, 115 °C (only for compounds **11** and **13**)

The chalcone derivatives were obtained via a base-catalyzed aldol condensation of the desired aldehyde substrate (Scheme 1, intermediates 1–3) with the corresponding ketone derivative to give the α,β-unsaturated carbonyl derivatives **5**–**14** (Scheme 2, general synthetic route A). The resulting chalcones ^1^H and ^13^C NMR spectra showed peaks consistent with a single product, with *J* = 15.8 Hz, suggesting that only the isomer E was formed.

**Scheme 3:**
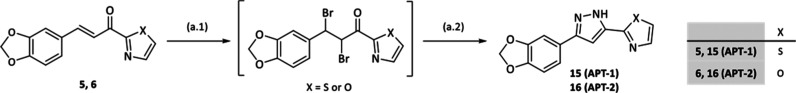
General Synthetic Route B Reagents and conditions: (a.1) Br_2_, CHCl_3_, 0 °C, 2 h; (a.2) hydrazine, EtOH, reflux, 24 h, 45–72%

**Scheme 4:**
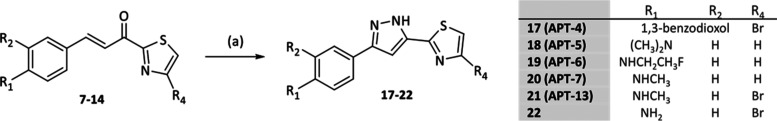
General Synthetic Route C Reagents and conditions: (a) hydrazine, I_2_, EtOH, reflux, 24 h, 22–40%; (a.2) HCl, 1.5–3 h, 115 °C (only for compounds **22–24**)

**Scheme 5:**
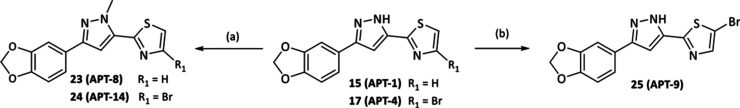
Synthesis of **APT-8**, **APT-9**, and **APT-14** Reagents and conditions: (a) NaH, MeI, 20 h, r.t., 56, 74%; (b) NBS, CHCl_3_, 18 h, 65 °C, 50%

**Scheme 6:**
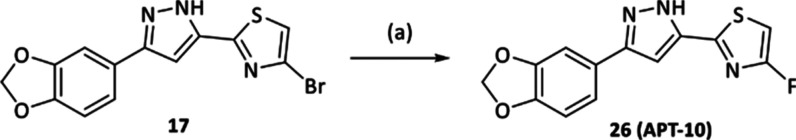
Synthesis of **APT-10** Reagents and conditions: (a) (1) *n*-BuLi, THF, 30 min, −70 °C, (2) *N*-fluorobenzenesulfonimide, THF, 2 h, −70 °C, 31%

Subsequently, compounds **15** and **16** were synthesized in two steps following the general synthetic route B (Scheme 3). The respective enones were subjected to the electrophilic addition of Br_2_, followed by their reaction with hydrazine monohydrate under reflux to afford the corresponding pyrazoles. Derivatives **17**–**22** in which ring A is substituted with *N*,*N*-dimethylaniline (**18**), *N*-methylaniline (**20**, **21**), *N*-(2-fluoroethyl)aniline (**19**), and aniline (**22**) moieties while rings B and C remain unchanged, and derivative **17**, in which the ring C is 4-bromo substituted, were accessed via a one-pot reaction (Scheme 4, general synthetic route C) from the corresponding α, β-unsaturated ketones by using hydrazine hydrate and I_2_ as oxidant under reflux.^22^ Compounds **25** and **26** were accessed by electrophilic halogenation from derivatives **15** and **17**, respectively (Schemes 5 and 6). The N-methylated analog of **15**, compound **23**, and the N-methylated analog of **17**, compound **24**, were obtained via methylation using methyl iodide (Scheme 5).

### In Vitro Competition Binding Assay on Recombinant α-Syn and Aβ Fibrils

Although α-syn aggregates represent the primary form of misfolded protein aggregates found in PD and other synucleinopathies, several studies have shown that Aβ aggregates often coincide with α-syn deposition. For in vivo application, probes targeting α-syn must exhibit high sensitivity and selectivity.

The APT library underwent in vitro testing to evaluate its binding affinity to human recombinant α-syn fibrils. Promising compounds were further tested on Aβ fibrils. Recent in silico studies have revealed that α-syn fibrils have multiple segments where binding can occur, and distinct classes of compounds can interact with different binding sites on the target.^23^ Since the DPP and APT class of compounds share similarities, the APT library was tested in a competition binding assay with [^3^H]MODAG-001 to determine its relative binding affinity and inhibition constant (*K*_i_). The binding assay measured the displacement of the radioligand by increasing concentrations of competitor compounds, using fixed concentrations of fibrils and radioligand. The results demonstrated reciprocal competition in binding to the target, suggesting that the two compounds compete for the same binding site.

**Figure 4:**
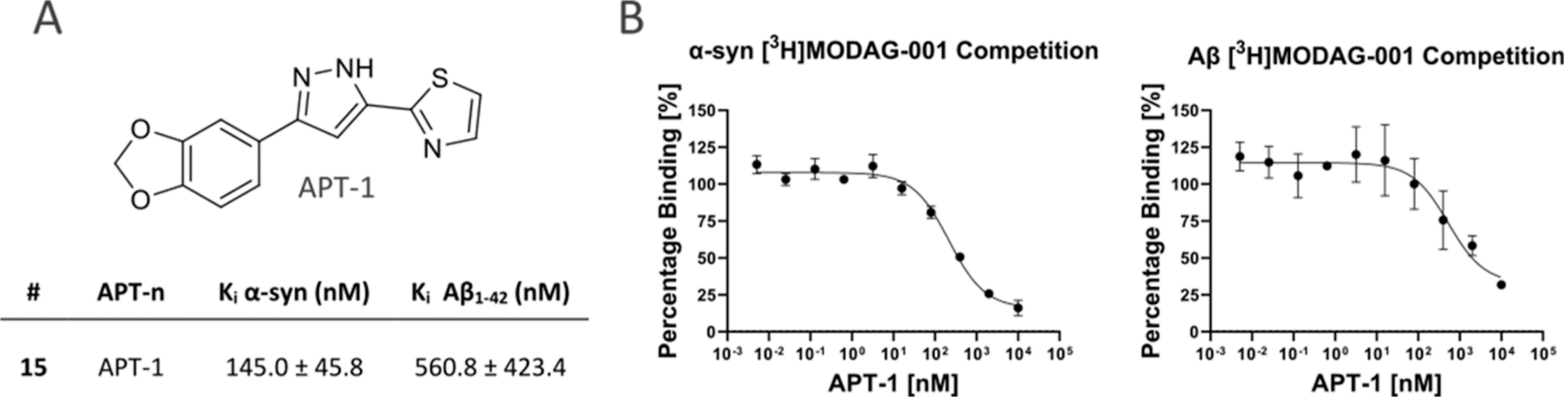
Binding affinity of **APT-1**. (A) Structure and binding affinity data on α-syn and Aβ (mean *K*_i_ ± SEM) of **APT-1** determined by competition binding assay against [^3^H]MODAG-001; (B) competition binding assay curves on α-syn and Aβ fibrils for **APT-1** against [^3^H]MODAG-001.

The replacement of a phenyl group with a thiazole ring in the DPP class, which led to **APT-1**, resulted in a moderate affinity for α-syn fibrils but a significantly lower binding to Aβ fibrils (Figure 4). Speculating that this modification could help improve selectivity to the target, we pursued further modification of scaffold **15** to enhance the binding affinity.

**Figure 5:**
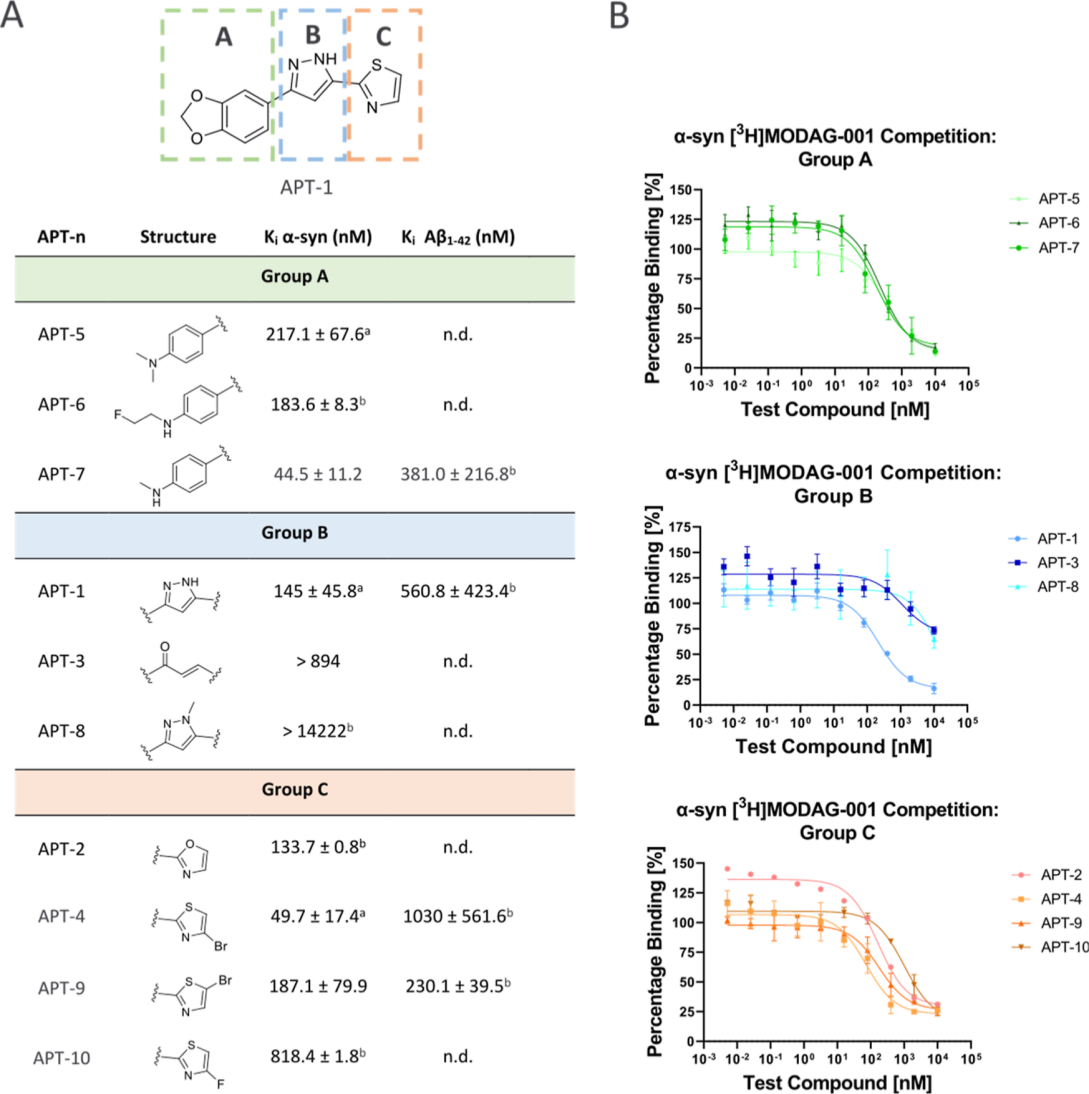
Results from competition binding assays against [^3^H]MODAG-001 on α-syn and Aβ fibrils. (A) Table with data, reported in MEAN *K*_i_ + SEM, three data points (dp); ^a^dp = 4, ^b^dp = 2; n.d. = not determined. (B) Competition binding assay curves of groups A, B, and C on α-syn fibrils.

On ring A, modification of the 1,3-benzodioxole ring with an *N*-methylaniline led to an improved affinity for the target (*K*_i_ α-syn = 44.5 ± 11.2 nM), while maintaining a high *K*_i_ on Aβ (*K*_i_ Aβ = 381.0 ± 216.8 nM). Replacing the methyl group with a fluoroethyl moiety in **APT-6**, on the other hand, resulted in modest affinity, suggesting that the size of the substituent also plays a role in target binding (Figure 5A,B).

On ring B, the replacement of the pyrazole ring with an enone had no positive impact on the binding affinity of the ligand with α-syn. This could be due to low affinity for the target or the absence of competition with [^3^H]MODAG-001 on the same binding pocket. As expected, methylation of the pyrazole ring also resulted in a loss of competition, suggesting the importance of an H-bond donor at this position (Figure 5A,B).

Regarding ring C, the substitution of a thiazole with an oxazole ring resulted in reduced binding. The effect of a halogen on the thiazole ring was also tested. The 4-bromothiazole **APT-4** was a better binder than the corresponding thiazole derivative **APT-1**, but replacing the bromine with fluorine led to a loss of binding. The 3-bromothiazole **APT-9** also leads to poor binding. Notably, the presence of a 4-bromine on the thiazole ring further improves the selectivity over Aβ (*K*_i_ Aβ = 1030 ± 561.6 nM). The improvement of affinity with the bromine in the 4-position of the thiazole ring (**APT-4** versus **APT-1**) can be attributed to strong halogen bond interaction with the target (Figure 5A,B). The halogen bond strength is strictly influenced by local changes in the electronic structure of the molecule.^24^ In 4-bromothiazole, the pyridine-like nitrogen in proximity to the halogen withdraws electron density, which increases the δ-hole and consequently its ability of halogen bonding. On the other hand, in 3-bromothiazole, the δ-hole of the bromine is surrounded by a negative surface due to the lone pair of the sulfur atom directed to the halogen. As a result, the bromine is less accessible to halogen bonding.

In general, these ligands demonstrated a much weaker affinity to Aβ than for α-syn fibrils, suggesting a general selectivity for α-syn over Aβ aggregates by this structural class.

**Figure 6:**
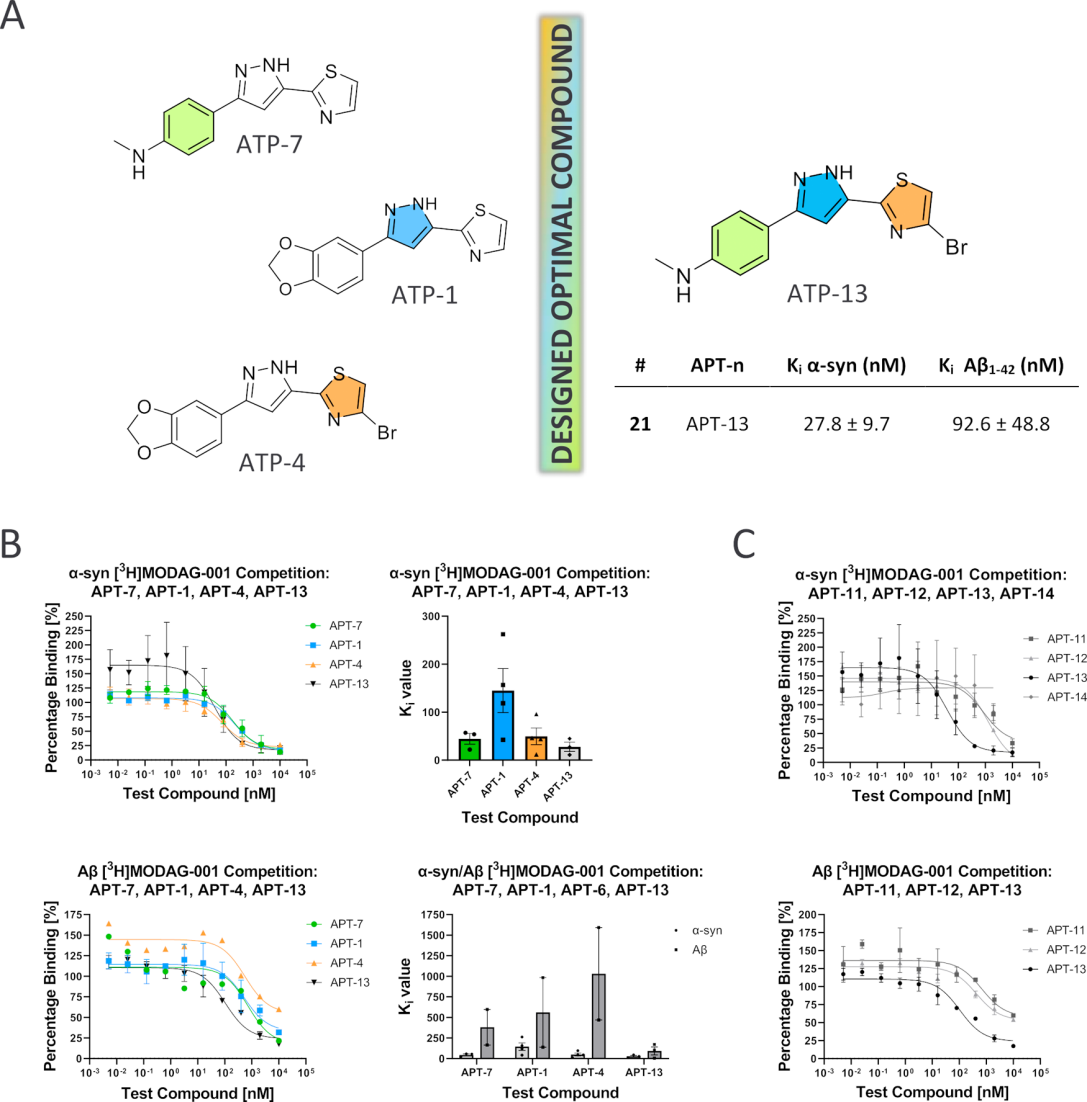
(A) Development of **APT-13**. (B) Binding affinity results comparison of **APT-7**, **APT-1**, **APT-4**, and **APT-13** shown in the binding competition curves (left) and *K*_i_ values (right) on α-syn (top) and Aβ (bottom). (C) Competition binding assay curves in comparison of **APT-11**, **APT-12**, **APT-13**, and **APT-14** on α-syn (top) and **APT-11**, **APT-12**, **APT-13** on Aβ (bottom).

Subsequent modifications of **APT-1** involved the three different rings of the molecule, and **APT-4** and **APT-7** showed a good affinity for α-syn fibrils with high selectivity over Aβ of >8 and >20-fold, respectively. A second generation of compounds **APT-11**, **APT-12**, **APT-13**, and **APT-14** were designed. Consistent with our previous results, **APT-13** displayed the highest binding affinity to α-syn (*K*_i_ α-syn = 27.8 ± 9.7 nM), and although the selectivity over Aβ was reduced (*K*_i_ Aβ = 92.6 ± 48.8 nM), it is still >3-fold (Figure 6A,B). The increased binding on Aβ could be explained by the higher log*P* of compound **APT-13** (clogP of **APT-13** = 3.51 versus *c*log*P* of **APT-1** = 2.61) due to the presence of a halogen in the structure.

It is important to note that **APT-13** still binds to Aβ fibrils nearly five times less effectively than the DPP compound, MODAG-001 (reported *K*_d_ for Aβ_1–42_ = 20 ± 10 nM, and in this work, *K*_d_ for Aβ_1–42_ = 6.2 ± 3 nM, due to the use of a different fibrils batch). As expected, **APT-12**, the 4-bromothiazole chalcone derivative, exhibited low affinity for α-syn (*K*_i_ α-syn = 198.1 ± 142.3 nM) and no competition toward Aβ, and **APT-11**, the nonbrominated analog, appeared in a loss of binding affinity for α-syn fibrils (*K*_i_ α-syn = 1009 ± 34 nM). Also consistent with our previous results, **APT-14**, the *N*-methyl derivative of **APT-4**, resulted in a loss of competition (Figure 6C).

In summary, according to the data described above, general SAR conclusions are available for further optimization of binding affinity and selectivity on α-syn: for ring A, the substitution with an *N*-monomethylaniline, as it is also seen in the recently published MODAG-005,^13^ is better than the *N*,*N*-dimenthylaniline and 1,3-dioxole; for ring B, the presence of the pyrazole ring is required; for ring C, 4-bromo substituted thiazole favors binding to α-syn.

### Synthesis of [^11^C]**APT-13**

**APT-13** exhibited the highest affinity to α-syn aggregates among the compounds tested, with a K_i_ value of 27.8 ± 9.7 nM. Despite its selectivity over Aβ_1–42_ being lower than that of other APTs tested, it still exceeds a 3-fold difference, making it a potential candidate for pathological α-syn PET imaging. Furthermore, the *N*-methylaniline ring allows for ^11^C-methylation at this site.

**Scheme 7:**
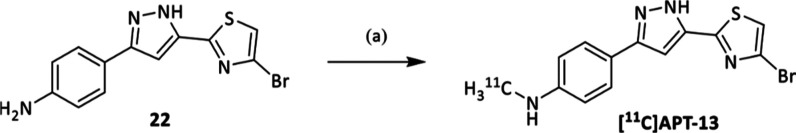
^11^C-Labeling Reaction of **APT-13** Reagents and conditions: (a) [^11^C]CH_3_OTf, 2-butanone, 75 °C, 2 min.

[^11^C]**APT-13** was radiolabeled by direct methylation of its aniline precursor **22** using [^11^C]methyl triflate ([^11^C]MeOTf) (Scheme 7). The methylation with [^11^C]MeOTf afforded [^11^C]**APT-13** in a radiochemical yield (RCY) of 13.5 ± 1.8% (from trapped [^11^C]MeOTf to formulated tracer) and a molar activity (*A*_m_) of 98.7 ± 12.7 GBq/μmol.

### Log*D* Determination and Serum Stability Assay of [^11^C]**APT-13**

High lipophilicity can drastically reduce the ability of a tracer to cross the BBB, as well as reduce the imaging resolution due to high NSB. Therefore, the log*D* value is an effective tool for the prediction of the response of a tracer. The log*D* value experimentally measured for [^11^C]**APT-13** was 3.16 (Table S3).

To assess the stability of [^11^C]**APT-13** against degradation, a serum stability assay was performed. The incubation of [^11^C]**APT-13** with human serum at 37 °C did not show evidence of degradation within 90 min (Figure S5).

### In Vivo PET Imaging of [^11^C]**APT-13** in Mice

**Figure 7:**
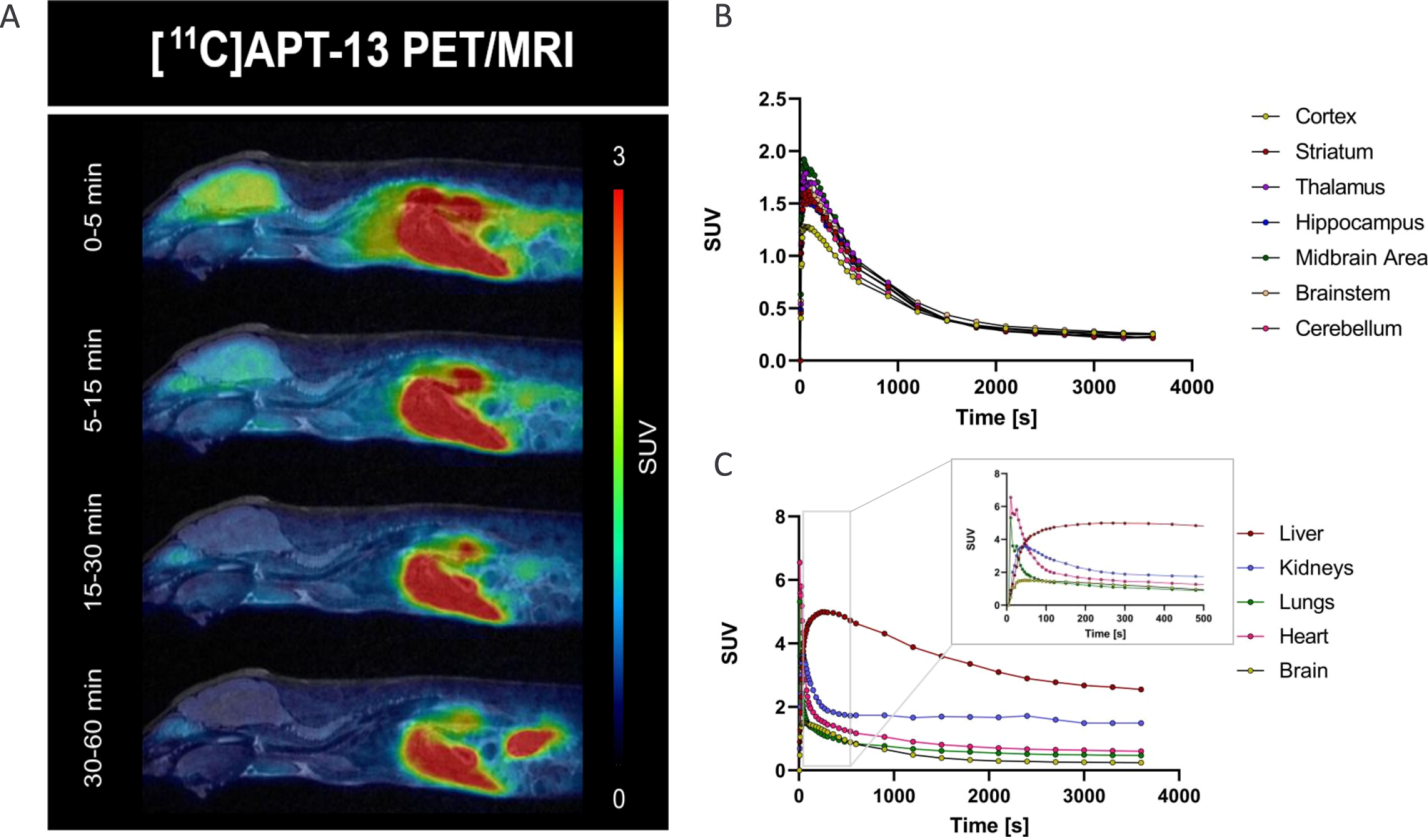
In vivo evaluation of the pharmacokinetic profile in wild-type mice after intravenous injection of [^11^C]**APT-13**. (A) whole-body PET-MRI sagittal images at different time points; (B) time–activity curves of selected brain regions; (C) time–activity curves of selected organs.

To assess the pharmacokinetic profile of [^11^C]**APT-13** in vivo, wild-type mice (*n* = 3) were iv injected, and the organ distribution was assessed over 60 min with dynamic PET and anatomical MR imaging (Figure 7A). [^11^C]**APT-13** demonstrated fast and high brain uptake with peak SUV of 1.94 ± 0.29 and rapid clearance from brain tissues (*t*_1/2_ = 9 ± 1 min) (Figure 7B). This result largely aligned with the BBB score prediction for **APT-13**, which was calculated to be 4.8.^21^ Tracer clearance was largest through the liver (SUV ≈ 5), and high uptake was also observed in the heart (SUV ≈ 6) and lungs (SUV ≈ 5). Uptake with fast clearance was also observed in the kidney, indicating renal excretion within the first few minutes (Figure 7C).

## Conclusions

This study demonstrated **APT-13** as a promising new ligand for α-syn aggregates. The low molecular weight compound, optimized for lipophilicity, was identified from a SAR study examining the three-ring systems of compound **15** (**APT**-**1**) as a scaffold. All compounds were readily accessed through efficient and easily scalable chemical syntheses. Notably, replacing a phenyl ring with a thiazole significantly improved selectivity for α-syn over Aβ, while maintaining high affinity for α-syn fibrils. Additionally, incorporating a bromine atom at position-4 of the thiazole ring enhanced its selective binding to α-syn aggregates and improved the affinity, presumably through a strong halogen bond interaction with the target. Additionally, this study confirmed the importance of the core pyrazole ring to act as a H-bond donor for ligand binding.

In vitro **APT-13**, identified after a SAR study of a library of APT, exhibited a binding affinity in the nanomolar range for α-syn fibrils (*K*_i_ = 27.8 ± 9.7 nM) and more than 3-fold greater selectivity over Aβ. However, it is important to note that structural differences between recombinant human fibrils and α-syn aggregates from human brain tissue have been reported^25,26^ and future studies using binding experiments in human pathological brain tissues and imaging studies in rodent models with different types of injected fibrils need to be considered. Furthermore, recent advances in high-resolution cryo-electron microscopy have unveiled various morphologies of α-syn fibrils, which appear to be distinct in PD compared to the protein folds found in MSA.^26,27^ Thus, characterization of differential binding to various fibril types will be of high interest in future work.

The potential of [^11^C]**APT-13**, methylated at the aniline group, as a desirable ligand for in vivo applications in detecting α-syn pathology was further supported by its excellent brain penetration and rapid washout from the brain.

Moreover, the APT class of compounds generally exhibited low activity on Aβ fibrils.

Consequently, the APT system presents a good candidate scaffold for developing low-lipophilic ligands that selectively bind to α-syn inclusions, serving as α-syn PET tracers.

## Experimental Section

### Chemistry

All chemicals were purchased from Sigma-Aldrich (St. Louis, MO, USA), abcr GmbH (Karlsruhe, Germany), Carl Roth (Karlsruhe, Germany) and used without any further purification. Reaction progress was monitored by thin-layer chromatography (TLC) on 0.20 mm Polygram SIL G/UV254 (silica gel 60) TLC plates (Macherey-Nagel, Düren, Germany) with the chosen eluent mixture and/or analytical liquid chromatography/mass spectrometry (LC/MS) analysis (ESI detector, Agilent, Santa Clara, CA, USA) equipped with a Luna 5 μm C18 (2) 100 Å 50 × 2 mm column (Phenomenex, Torrance, CA, USA) under the following gradient: 0–7.60 min (0 to 100% B), 7.60–8.80 (100% B), 8.80–9.30 min (100 to 0% B), 9.30–13.0 min (0% B); solvent A: 0.1% formic acid (FA) in H_2_O; solvent B: acetonitrile (ACN); flow: 0.4 mL/min. Purification of crude compounds was performed using an automated flash chromatography on an Isolera 4 system (Biotage, Uppsala, Sweden). ^1^H and ^13^C NMR spectra were acquired on an Avance III AV 600 (^1^H: 600.13 MHz; ^13^C: 150.61 MHz) spectrometer (Bruker, Billerica, MA, USA). All chemical shifts (δ) are reported as parts per million (ppm) and referenced to residual solvent peaks (chloroform-*d*: δH = 7.26, δC = 77.16, DMSO-*d*_6_: δH = 2.50, δC = 39.52). ^1^H and ^13^C NMR spectra of compounds 1–26 are reported in the Supporting Information.

### General Procedures for the Synthesis of APTs

General procedure A:^28^ To a mixture of the corresponding ketone (1 mmol) and aldehyde (1.3 mmol) in ethanol, piperidine (1.5 mmol) was added, and the reaction mixture was refluxed at 70 °C under stirring. After the completion of the reaction (3–5 h), ethanol was distilled off, and the residue was poured into ice water. It was kept overnight in the refrigerator. The resulting solid was collected by filtration, washed with distilled water, and purified by flash column chromatography to get the corresponding thiazolyl chalcone in 46–94%.

General procedure B: b.1. To a stirred solution of the α,β-unsaturated carbonyl derivative (1 mmol) in chloroform (5 mL) was added a solution of dibromine (1.5 mmol) dropwise in chloroform (2 mL) at 0 °C. After being stirred for 2.5 h at 0 °C, the mixture was diluted with petroleum ether (PE), refrigerated, and the resulting precipitate collected by filtration, washed with *n*-hexane (10 mL), and dried. The crude product was used for step b.2 without further purification. b.2. A solution of b.1 (1 mmol) and hydrazine hydrate (5 mmol) in ethanol (10 mL) was heated under reflux for 24 with stirring. The reaction mixture was extracted with ethyl acetate (EA), washed with brine and dried over anhydrous MgSO_4_, concentrated under vacuum, and purified through silica gel column chromatography using a mixture of EA and PE as eluent (35–45% of EA) to afford the corresponding pyrazoles in 45–72%.

General procedure C:^22^ To a stirred solution of the α,β-unsaturated carbonyl derivative (1 mmol) and hydrazine HCl salts (3 mmol) in ethanol (5 mL) were added molecular iodine (3 mmol), and then the reaction was heated to reflux for 24 h under a nitrogen atmosphere. The reaction mixture was concentrated, quenched with 5% Na_2_S_2_O_3_, and then extracted with EA (10 mL × 3). The combined organic layer was dried over anhydrous MgSO_4_, concentrated under vacuum, and purified through silica gel column chromatography using a mixture of EA and PE as eluent to afford the corresponding pyrazoles in 22–40%.

General procedure D: To a stirred solution of Boc-aniline derivative (1 mmol), was added HCl 5 M (1 mL) at 115 °C. After the completion (1.5–3 h), the reaction mixture was cooled to room temperature, washed with 3 M NaOH (5 mL), and extracted with EA. The crude product was purified by silica gel chromatography (PE/EA, gradient 65/35–50/50) to give the corresponding final products.

### *tert*-Butyl (4-Formylphenyl)carbamate (**1**)

A mixture of 4-aminobenzaldehyde (1.00 g, 121 mmol, 1.0 equiv) and di-*tert*-butyldicarbonate (4.64 g, 21.1 mmol, 2.5 equiv) in THF (20 mL) was heated to reflux for 24 h. The mixture was poured into water (30 mL) and extracted with EtOAc (3 × 35 mL). The combined organic layers were washed with water and brine, dried over MgSO_4_, filtered, and concentrated. The crude product was purified by flash column chromatography (PE/EA, gradient 80/20–60/40) to give the pure product as light-yellow solid (900 mg, 48%). ^1^H NMR (600 MHz, chloroform-*d*): δ 9.87 (s, 1H), 7.80 (d, *J* = 8.6 Hz, 2H), 7.54 (d, *J* = 8.6 Hz, 2H), 7.05 (s, 1H), 1.51 (s, 9H). ^13^C NMR (151 MHz, chloroform-*d*): δ 191.01, 152.06, 144.20, 131.34, 131.28, 117.81, 81.54, 28.25. Mass found (ESI^+^), 222.150 [M + H]^+^.

### *tert*-Butyl (4-Formylphenyl)(methyl)carbamate (**2**)

To a solution of **1** (25 mg, 0.11 mmol, 1.0 equiv) in anhydrous DMF (1.5 mL) was added sodium hydride, 60% dispersion in mineral oil (5.7 mg, 0.15 mmol, 1.3 equiv) under argon at 0 °C. After the reaction mixture was stirred at room temperature for 30 min, methyl iodide (10.9 μL, 0.17 mmol, 1.5 equiv) was added with additional stirring overnight. After quenching with saturated NH_4_Cl aqueous solution, the mixture was extracted with EA. The extract was washed with saturated NaHCO_3_ aqueous solution and brine and dried over MgSO_4_. After filtration, the filtrate was concentrated in vacuo. The residue was purified by silica gel column chromatography (PE/EA, gradient 80/20–60/40) to obtain the title product as a white solid (19 mg, 72%). ^1^H NMR (600 MHz, chloroform-*d*): δ 9.94 (s, 1H), 7.83 (d, *J* = 8.6 Hz, 2H), 7.44 (d, *J* = 8.5 Hz, 2H), 3.31 (s, 3H), 1.47 (s, 10H). ^13^C NMR (151 MHz, chloroform-*d*): δ 191.27, 154.00, 149.27, 132.66, 130.17, 124.66, 81.43, 36.83, 28.27. Mass found (ESI+): 236.100 [M + H]^+^.

### *tert*-Butyl (2-Fluoroethyl)(4-formylphenyl)carbamate (**3**)

To a solution of **1** (50 mg, 0.23 mmol, 1.0 equiv) in anhydrous DMF (5.5 mL) was added sodium hydride, 60% dispersion in mineral oil (18.3 mg, 0.46 mmol, 2 equiv) under argon at 0 °C. After the reaction mixture was stirred at room temperature for 30 min, 1-fluoro-2-iodoethane (26.7 μL, 0.34 mmol, 1.5 equiv) was added with additional stirring overnight. After quenching with saturated NH_4_Cl aqueous solution, the mixture was extracted with EA. The extract was washed with saturated NaHCO_3_ aqueous solution and brine and dried over MgSO_4_. After filtration, the filtrate was concentrated in vacuo. The residue was purified by silica gel column chromatography (PE/EA, gradient 80/20–60/40) to obtain the title product as an off-white solid (36 mg, 59%). ^1^H NMR (600 MHz, chloroform-*d*): δ 9.98 (s, 1H), 7.85 (d, *J* = 8.5 Hz, 2H), 7.44 (d, *J* = 8.5 Hz, 1H), 4.62 (dt, *J* = 47.4, 4.9 Hz, 2H), 3.96 (dt, *J* = 24.8, 4.9 Hz, 2H), 1.46 (s, 9H). ^13^C NMR (151 MHz, chloroform-*d*): δ 191.08, 190.80, 153.77, 148.33, 133.80, 131.20, 130.27, 126.84 (d, *J* = 2.2 Hz), 117.84, 83.73–79.71 (m), 50.65 (d, *J* = 21.0 Hz), 28.22. Mass found (ESI^+^), 268.050 [M + H]^+^, 290.100 [M + Na]^+^.

### 1-(4-Bromothiazol-2-yl)ethan-1-one (**4**)

*n*-Butyllithium (5.57 mL, 2.5 M in hexane, 13.92 mmol, 1.15 equiv) was added dropwise to a solution of 2,4-dibromothiazole (3.00 g, 12.10 mmol, 1.0 equiv) in THF (20 mL) under an argon atmosphere at −78 °C. After stirring at −78 °C for 20 min, *N*-acetyl morpholine (2.12 mL, 2.34 g, 18.15 mmol, 1.5 equiv) was added dropwise and stirred for 3 h at −78 °C. The reaction mixture was quenched with sat. NaHCO_3_ (20 mL) and extracted with ethyl acetate (30 mL × 3). The combined organic layers were washed with brine (30 mL), dried over MgSO_4_, filtered, concentrated, and purified by flash chromatography using a silica gel column with a PE/EA (95/5) to afford the desired acetyl thiazole **4** as a white solid (1.54 g, 6.55 mmol, 62%). ^1^H NMR (600 MHz, chloroform-*d*): δ (ppm) = 7.57 (s, 1H), 2.67 (s, 3H); ^13^C NMR (151 MHz, chloroform-*d*): δ 190.40, 166.91, 126.92, 125.11, 25.86. Mass found (ESI^+^), 207.900 [M + H]^+^.

### (*E*)-3-(Benzo[*d*][1,3]dioxol-5-yl)-1-(thiazol-2-yl)prop-2-en-1-one (**5**, **APT-3**)

The title compound was prepared according to general procedure A. To a mixture of 1-(thiazol-2-yl)ethan-1-one (600 mg, 0.499 mL, 4.81 mmol, 1.0 equiv) and benzo[*d*][1,3]dioxole-5-carbaldehyde (795 mg, 5.30 mmol, 1.2 equiv) in ethanol (5 mL), piperidine (0.520 mL, 5.06 mmol, 1.05 equiv) was added and the reaction mixture refluxed for 4 h. After the completion of the reaction, ethanol was distilled off, and the residue was poured into ice water. It was kept overnight in the refrigerator. The resulting solid was collected by filtration, washed with distilled water, and purified by flash column chromatography to give the corresponding thiazolyl chalcone as a yellow solid (774 mg, 3 mmol, 62%). ^1^H NMR (600 MHz, chloroform-*d*): δ 8.04 (d, *J* = 3.0 Hz, 1H), 7.93 (d, *J* = 15.9 Hz, 1H), 7.77 (d, *J* = 15.9 Hz, 1H), 7.68 (d, *J* = 3.0 Hz, 1H), 7.24 (d, *J* = 1.7 Hz, 1H), 7.19 (dd, *J* = 8.0, 1.7 Hz, 1H), 6.85 (d, *J* = 8.0 Hz, 1H), 6.02 (s, 2H). ^13^C NMR (151 MHz, chloroform-*d*): δ 181.51, 168.92, 150.40, 148.51, 145.65, 144.60, 129.33, 126.10, 125.92, 118.78, 108.66, 107.09, 101.69. Mass found (ESI^+^), 260.100 [M + H]^+^.

### (*E*)-3-(Benzo[*d*][1,3]dioxol-5-yl)-1-(oxazol-2-yl)prop-2-en-1-one (**6**)

A solution of 1-(benzo[*d*][1,3]dioxol-5-yl)ethan-1-one (180 mg, 1.12 mmol, 1 equiv), oxazole-2-carbaldehyde (0.118 mL, 149 mg, 1.45 mmol, 1.3 equiv), NaOH (60 mg, 1.45 mmol, 1.3 equiv), and Ba(OH)_2_·8H_2_O (21 mg, 0.06 mmol, 0.05 equiv) in methanol (5 mL) were stirred at room temperature for 24 h. The reaction mixture was cooled to +4 °C, and a resulting precipitate was collected by filtration and purified by flash chromatography using a silica gel column with a PE/EA (95/5) to provide the desired compound as a yellow powder (185 mg, 0.76 mmol, 68%). ^1^H NMR (600 MHz, chloroform-*d*): δ 7.83 (d, *J* = 15.6 Hz, 1H), 7.73 (s, 1H), 7.66 (dd, *J* = 8.2, 1.7 Hz, 1H), 7.55–7.50 (m, 2H), 7.30 (s, 1H), 6.89 (d, *J* = 8.1 Hz, 1H), 6.06 (s, 2H). ^13^C NMR (151 MHz, chloroform-*d*): δ 186.91, 160.25, 152.31, 148.55, 139.81, 132.18, 129.63, 127.96, 127.38, 125.22, 108.40, 108.03, 101.99. Mass found (ESI^+^), 244.050 [M + H]^+^.

### (*E*)-3-(Benzo[*d*][1,3]dioxol-5-yl)-1-(4-bromothiazol-2-yl)prop-2-en-1-one (**7**)

The title compound was prepared according to general procedure A using 1-(4-bromothiazol-2-yl)ethan-1-one (550 mg, 2.70 mmol), benzo[*d*]dioxole-5-carbaldehyde (490 mg, 3.24 mmol), piperidine (0.326 mL, 281 mg, 3.24 mmol), ethanol (90 mL) to give the product as a yellow solid (745 mg, 82%). ^1^H NMR (600 MHz, chloroform-*d*): δ 7.93 (d, *J* = 15.8 Hz, 1H), 7.71 (d, *J* = 15.8 Hz, 1H), 7.59 (s, 1H), 7.20 (dd, *J* = 8.0, 1.7 Hz, 1H), 6.86 (d, *J* = 8.0 Hz, 1H), 6.05 (s, 2H). ^13^C NMR (151 MHz, chloroform-*d*): δ 179.25, 167.69, 149.68, 147.52, 145.63, 128.04, 125.79, 125.41, 123.79, 116.73, 107.69, 106.16, 100.78. Mass found (ESI^+^), 339.900 [M + H]^+^.

### (*E*)-3-(4-(Dimethylamino)phenyl)-1-(thiazol-2-yl)prop-2-en-1-one (**8**)

The title compound was prepared according to general procedure A using 1-(thiazol-2-yl)ethan-1-one (450 mg, 3.61 mmol), 4-(dimethylamino)benzaldehyde (593 mg, 3.97 mmol), piperidine (0.382 mL, 329 mg, 3.79 mmol), ethanol (30 mL) to give an orange solid (873 mg, 94%). ^1^H NMR (600 MHz, chloroform-*d*): δ 7.95 (d, *J* = 3.0 Hz, 1H), 7.92 (d, *J* = 15.7 Hz, 1H), 7.66 (d, *J* = 15.7 Hz, 1H), 7.58–7.53 (m, 3H), 6.66 (d, *J* = 8.5 Hz, 2H), 2.98 (s, 6H). ^13^C NMR (151 MHz, chloroform-*d*): δ 181.36, 169.81, 152.04, 146.71, 144.43, 131.09, 125.58, 115.53, 112.30, 40.41, 40.38. Mass found (ESI^+^), 259.050 [M + H]^+^.

### *tert*-Butyl (*E*)-(2-Fluoroethyl)(4-(3-oxo-3-(thiazol-2-yl)prop-1-en-1-yl)phenyl)carbamate (**9**)

The title compound was prepared according to general procedure A using 1-(thiazol-2-yl)ethan-1-one (120 mg, 0.95 mmol), *tert*-butyl (2-fluoroethyl)(4-formylphenyl)carbamate [**3**] (325 mg, 1.19 mmol), piperidine (0.120 mL, 104 mg, 1.19 mmol), ethanol (35 mL) to give the product as a light orange solid (149 mg, 42%). ^1^H NMR (600 MHz, chloroform-*d*): δ 8.06 (d, *J* = 3.0 Hz, 1H), 7.99 (d, *J* = 15.9 Hz, 1H), 7.91 (d, *J* = 16.0 Hz, 1H), 7.70 (d, *J* = 3.0 Hz, 1H), 7.68 (d, *J* = 8.5 Hz, 2H), 7.31 (d, *J* = 8.5 Hz, 2H), 4.61 (dt, *J* = 47.3, 5.0 Hz, 2H), 3.93 (dt, *J* = 24.7, 5.0 Hz, 2H), 1.45 (s, 9H). ^13^C NMR (151 MHz, chloroform-*d*): δ 181.58, 168.64, 154.10, 145.12, 144.96, 144.70, 132.29, 129.43, 127.10, 126.46, 120.53, 81.78 (d, *J* = 144.5 Hz), 81.13, 50.62 (d, *J* = 21.0 Hz), 28.27. Mass found (ESI+), 399.00 [M + Na]^+^.

### *tert*-Butyl (*E*)-Methyl(4-(3-oxo-3-(thiazol-2-yl)prop-1-en-1-yl)phenyl)carbamate (**10**)

The title compound was prepared according to general procedure A using 1-(thiazol-2-yl)ethan-1-one (120 mg, 0.95 mmol), *tert*-butyl (4-formylphenyl)(methyl)carbamate [**2**] (252 mg, 1.05 mmol), piperidine (0.144 mL, 124 mg, 1.4 mmol), ethanol (20 mL) to give the product as a yellow solid (189 mg, 57%). ^1^H NMR (600 MHz, chloroform-*d*): δ 8.04 (d, *J* = 3.0 Hz, 1H), 7.96 (d, *J* = 15.9 Hz, 1H), 7.84 (d, *J* = 15.9 Hz, 1H), 7.68 (d, *J* = 3.0 Hz, 1H), 7.63 (d, *J* = 8.6 Hz, 2H), 7.44 (d, *J* = 8.3 Hz, 2H), 6.82 (s, 1H), 1.51 (s, 9H). ^13^C NMR (151 MHz, chloroform-*d*): δ 181.71, 169.05, 152.43, 145.70, 144.73, 141.36, 130.29, 129.40, 126.36, 118.93, 118.42, 81.26, 28.40. Mass found (ESI^+^), 345.150 [M + H]^+^, 367.050 [M + Na]^+^.

### (*E*)-3-(4-(Methylamino)phenyl)-1-(thiazol-2-yl)prop-2-en-1-one (**11**, **APT-11**)

The title compound was prepared according to general procedure D using *tert*-butyl (*E*)-methyl(4-(3-oxo-3-(thiazol-2-yl)prop-1-en-1-yl)phenyl)carbamate [**9**] (20 mg, 0.06 mmol), HCl 5 M (0.5 mL) to give the desired product as a red solid (11 mg, 77%). ^1^H NMR (600 MHz, chloroform-*d*): δ 8.03 (d, *J* = 3.0 Hz, 1H), 7.98 (d, *J* = 15.7 Hz, 1H), 7.74 (d, *J* = 15.8 Hz, 1H), 7.65 (d, *J* = 3.0 Hz, 1H), 7.59 (d, *J* = 8.6 Hz, 2H), 6.66 (d, *J* = 8.7 Hz, 2H), 2.91 (s, 3H). ^13^C NMR (151 MHz, chloroform-*d*): δ 181.46, 169.65, 151.06, 146.74, 144.49, 131.29, 125.79, 124.57, 115.67, 112.90, 30.72. Mass found (ESI+): 245.100 [M + H]^+^.

### *tert*-Butyl (*E*)-(4-(3-(4-Bromothiazol-2-yl)-3-oxoprop-1-en-1-yl)phenyl)(methyl)carbamate (**12**)

The title compound was prepared according to general procedure A using 1-(4-bromothiazol-2-yl)ethan-1-one (120 mg, 0.59 mmol), *tert*-butyl (4-formylphenyl)(methyl)carbamate [**2**] (155 mg, 0.65 mmol), piperidine (0.089 mL, 77 mg, 0.88 mmol), ethanol (15 mL) to give the product as a light yellow solid (214, 86%). ^1^H NMR (600 MHz, chloroform-*d*): δ 7.97 (d, *J* = 15.9 Hz, 1H), 7.81 (d, *J* = 15.9 Hz, 1H), 7.67 (d, *J* = 8.6 Hz, 2H), 7.59 (s, 1H), 7.32 (d, *J* = 8.6 Hz, 2H), 3.29 (s, 3H), 1.47 (s, 9H). ^13^C NMR (151 MHz, chloroform-*d*): δ 180.63, 168.83, 154.52, 146.83, 146.39, 131.22, 129.77, 127.17, 125.39, 125.31, 119.43, 81.37, 37.21, 28.62. Mass found (ESI^+^), 424.950 [M + H]^+^, 447.000 [M + Na]^+^.

### (*E*)-1-(4-Bromothiazol-2-yl)-3-(4-(methylamino)phenyl)prop-2-en-1-one (**13**, **APT-12**)

The title compound was prepared according to general procedure D using *tert*-butyl (*E*)-(4-(3-(4-bromothiazol-2-yl)-3-oxoprop-1-en-1-yl)phenyl)(methyl)carbamate [**11**] (20 mg, 0.05 mmol), HCl 5 M (0.5 mL) to give the desired product as an orange solid (10 mg, 65%). ^1^H NMR (600 MHz, chloroform-*d*): δ 7.98 (d, *J* = 15.7 Hz, 1H), 7.67 (d, *J* = 15.7 Hz, 1H), 7.61 (d, *J* = 8.7 Hz, 2H), 7.54 (s, 1H), 6.71 (d, *J* = 8.5 Hz, 2H), 2.93 (s, 4H). ^13^C NMR (151 MHz, chloroform-*d*): δ 179.06, 168.49, 149.84, 146.48, 130.52, 129.09, 125.53, 123.93, 123.27, 114.17, 112.31, 28.67. Mass found (ESI^+^), 324.900 [M + H]^+^, 346.950 [M + Na]^+^.

### (*E*)-3-(4-Aminophenyl)-1-(4-bromothiazol-2-yl)prop-2-en-1-one (**14**)

The title compound was prepared according to general procedure A using 1-(4-bromothiazol-2-yl)ethan-1-one (35 mg, 0.29 mmol), 4-aminobenzaldehyde (23 mg, 0.19 mmol), piperidine (0.026 mL, 23 mg, 0.26 mmol), ethanol (5 mL) to give the product as an orange solid (45 mg, 85%). ^1^H NMR (600 MHz, DMSO-*d*_6_): δ 8.28 (s, 1H), 7.84 (d, *J* = 15.6 Hz, 1H), 7.56 (d, *J* = 8.2 Hz, 2H), 7.47 (d, *J* = 15.6 Hz, 1H), 6.62 (d, *J* = 8.2 Hz, 2H), 6.19 (s, 2H). ^13^C NMR (151 MHz, DMSO-*d*_6_): δ 179.41, 169.70, 153.75, 148.37, 132.45, 126.66, 125.97, 121.85, 114.22, 112.54. Mass found (ESI^+^), 310.950 [M + H]^+^.

### 2-(3-(Benzo[d][1,3]dioxol-5-yl)-1*H*-pyrazol-5-yl)thiazole (**15**, **APT-1**)

The title compound was prepared according to general procedure B using (*E*)-3-(benzo[d][1,3]dioxol-5-yl)-1-(thiazol-2-yl)prop-2-en-1-one [**5**] (150 mg, 0.64 mmol), dibromine (0.037 mL, 114 mg, 0.71 mmol), chloroform (10 mL), hydrazine hydrate (0.088 mL, 88 mg, 2.5 mmol), ethanol (5 mL) to give the desired product as a light brown solid (41 mg, 38%). ^1^H NMR (600 MHz, DMSO-*d*_6_): δ 13.48 (s, 1H), 7.87 (s, 1H), 7.68 (s, 1H), 7.43 (d, *J* = 1.8 Hz, 1H), 7.35 (d, *J* = 8.3 Hz, 1H), 7.12 (s, 1H), 7.03 (d, *J* = 8.1 Hz, 1H), 6.08 (s, 2H). ^13^C NMR (151 MHz, DMSO-*d*_6_): δ 162.16, 148.39, 147.95, 147.70, 144.24, 143.55, 123.26, 119.78, 109.29, 106.37, 101.84, 100.15. Mass found (ESI^+^), 272.100 [M + H]^+^.

### 2-(3-(Benzo[*d*][1,3]dioxol-5-yl)-1*H*-pyrazol-5-yl)oxazole (**16**, **APT-2**)

The title compound was prepared according to general procedure B using (*E*)-3-(benzo[*d*][1,3]dioxol-5-yl)-1-(oxazol-2-yl)prop-2-en-1-one [**6**] (240 mg, 1.10 mmol), dibromine (0.057 mL, 177 mg, 1.32), chloroform (10 mL), hydrazine hydrate (0.146 mL, 146 mg, 3.64 mmol), ethanol (7 mL) to give the desired product as a light brown solid (131 mg, 78%). ^1^H NMR (600 MHz, DMSO-*d*_6_): δ 13.62 (s, 1H), 8.16 (s, 1H), 7.43 (d, *J* = 1.9 Hz, 2H), 7.37–7.32 (m, 2H), 7.13 (d, *J* = 2.1 Hz, 1H), 7.03 (d, *J* = 8.0 Hz, 1H), 6.08 (s, 2H). ^13^C NMR (151 MHz, DMSO-*d*_6_): δ 157.49, 148.41, 147.98, 143.93, 141.74, 139.76, 128.44, 123.08, 119.79, 109.30, 106.37, 101.99, 101.86. Mass found (ESI+): 256.050 [M + H]^+^.

### 2-(3-(Benzo[*d*][1,3]dioxol-5-yl)-1*H*-pyrazol-5-yl)-4-bromothiazole (**17**, **APT-4**)

The title compound was prepared according to general procedure C using (*E*)-3-(benzo[*d*][1,3]dioxol-5-yl)-1-(4-bromothiazol-2-yl)prop-2-en-1-one [**7**] (340 mg, 1.02 mmol), hydrazine monohydrate (0.118 mL, 118 mg, 2.31 mmol), I_2_ (548 mg, 2.16 mmol), ethanol (40 mL) to afford the desired product as a white solid (137 mg, 39%). ^1^H NMR (600 MHz, DMSO-*d*_6_): δ 13.70 (s, 1H), 7.79 (s, 1H), 7.44 (d, *J* = 1.9 Hz, 1H), 7.37 (dd, *J* = 7.7, 2.2 Hz, 1H), 7.17 (s, 1H), 7.01 (d, *J* = 8.0 Hz, 1H), 6.08 (s, 2H). ^13^C NMR (151 MHz, DMSO-*d*_6_): δ 163.26, 148.38, 148.00, 145.12, 124.59, 123.42, 119.84, 117.99, 114.05, 109.25, 106.42, 101.84, 100.49. Mass found (ESI^+^), 351.000 [M + H]^+^.

### *N*,*N*-Dimethyl-4-(5-(thiazol-2-yl)-1*H*-pyrazol-3-yl)aniline (**18**, **APT-5**)

The title compound was prepared according to general procedure C using (*E*)-3-(4-(dimethylamino)phenyl)-1-(thiazol-2-yl)prop-2-en-1-one [**8**] (125 mg, 0.45 mmol), hydrazine monohydrate (0.035 mL, 35 mg, 0.68 mmol), I_2_ (150 mg, 0.59 mmol), ethanol (15 mL) to give the desired product as an orange solid (27 mg, 22%). ^1^H NMR (600 MHz, DMSO-*d*_6_): δ 13.30 (s, 1H), 7.86 (d, *J* = 3.2 Hz, 1H), 7.64 (d, *J* = 8.6 Hz, 3H), 6.97 (s, 1H), 6.79 (d, *J* = 8.8 Hz, 2H), 2.95 (s, 6H). ^13^C NMR (151 MHz, DMSO-*d*_6_): δ 162.47, 150.83, 147.60, 145.06, 143.47, 126.76, 119.57, 116.91, 112.71, 98.64, 40.36. Mass found (ESI^+^), 271.050 [M + H]^+^.

### *N*-(2-Fluoroethyl)-4-(5-(thiazol-2-yl)-1*H*-pyrazol-3-yl)aniline (**19**, **APT-6**)

The title compound was prepared according to general procedure C (*tert*-butyl (*E*)-(2-fluoroethyl)(4-(3-oxo-3-(thiazol-2-yl)prop-1-en-1-yl)phenyl)carbamate [**13**] (45 mg, 0.11 mmol), hydrazine monohydrate (0.011 mL, 11 mg, 0.22 mmol), I_2_ (57 mg, 0.22 mmol), ethanol (5 mL). The crude Boc-protected derivative was deprotected according to general procedure D to give the desired compound as an off-white solid (11 mg, 34%). ^1^H NMR (600 MHz, DMSO-*d*_6_): δ 13.31 (s, 1H), 7.85 (d, *J* = 3.3 Hz, 1H), 7.66 (d, *J* = 3.3 Hz, 1H), 7.56 (d, *J* = 8.3 Hz, 2H), 6.93 (s, 1H), 6.70 (d, *J* = 8.4 Hz, 2H), 6.15 (t, *J* = 5.8 Hz, 1H), 4.57 (dt, *J* = 47.6, 5.0 Hz, 2H), 3.45–3.38 (m, 2H). ^13^C NMR (151 MHz, DMSO): δ 148.82, 147.07, 144.67, 143.02, 127.93, 126.46, 119.17, 114.44, 112.15, 98.00, 82.51 (d, *J* = 165.4 Hz), 42.99 (d, *J* = 20.4 Hz). Mass found (ESI+), 389.050 [M + H]^+^.

### *N*-Methyl-4-(5-(thiazol-2-yl)-1*H*-pyrazol-3-yl)aniline (**20**, **APT-7**)

The title compound was prepared according to general procedure C using (*tert*-butyl (*E*)-methyl(4-(3-oxo-3-(thiazol-2-yl)prop-1-en-1-yl)phenyl)carbamate [**9**] (75 mg, 0.20 mmol), hydrazine monohydrate (0.021 mL, 21 mg, 0.41 mmol), I_2_ (104 mg, 0.41 mmol), ethanol (5.5 mL). The crude Boc-protected derivative was deprotected according to general procedure D to give the desired product as a light brown solid (21 mg, 40%). ^1^H NMR (600 MHz, DMSO-*d*_6_): δ 13.25 (s, 1H), 7.85 (d, *J* = 3.3 Hz, 1H), 7.66 (d, *J* = 3.3 Hz, 1H), 7.55 (d, *J* = 8.4 Hz, 2H), 6.91 (s, 1H), 6.61 (d, *J* = 8.4 Hz, 2H), 5.97 (s, 1H), 2.71 (s, 3H). ^13^C NMR (151 MHz, DMSO-*d*_6_): δ 162.07, 150.63, 147.53, 145.36, 143.47, 126.87, 119.57, 115.97, 112.10, 98.29, 30.00. Mass found (ESI+), 257.100 [M + H]^+^.

### 4-(5-(4-Bromothiazol-2-yl)-1*H*-pyrazol-3-yl)-*N*-methylaniline (**21**, **APT-13**)

The title compound was prepared according to general procedure C using *tert*-butyl (*E*)-(4-(3-(4-bromothiazol-2-yl)-3-oxoprop-1-en-1-yl)phenyl)(methyl)carbamate [**11**] (70 mg, 0.16 mmol), hydrazine monohydrate (0.016 mL, 16 mg, 0.31 mmol), I_2_ (79 mg, 0.31 mmol), ethanol (5.5 mL). The crude Boc-protected derivative was deprotected according to general procedure D to give the desired compound as an orange solid (21 mg, 31%). ^1^H NMR (600 MHz, DMSO-*d*_6_): δ 13.34 (s, 1H), 7.75 (s, 1H), 7.56 (d, *J* = 8.2 Hz, 2H), 6.95 (s, 1H), 6.62 (d, *J* = 7.4 Hz, 2H), 2.72 (s, 3H). ^13^C NMR (151 MHz, DMSO-*d*_6_): δ 163.44, 159.74, 150.27, 129.82, 126.49, 124.06, 117.30, 115.76, 111.66, 98.04, 29.55. Mass found (ESI+), 336.00 [M + H]^+^.

### 4-(5-(4-Bromothiazol-2-yl)-1*H*-pyrazol-3-yl)aniline (**22**)

The title compound was prepared according to general procedure C using (*E*)-3-(4-aminophenyl)-1-(4-bromothiazol-2-yl)prop-2-en-1-one [**14**] (50 mg, 0.15 mmol), hydrazine monohydrate (0.016 mL, 16 mg, 0.30 mmol), I_2_ (77 mg, 0.30 mmol), ethanol (2 mL) to give the desired product as a light orange solid (12 mg, 25%). ^1^H NMR (600 MHz, DMSO-*d*_6_): δ 13.37 (s, 1H), 7.77 (s, 1H), 7.50 (d, *J* = 8.1 Hz, 2H), 6.95 (s, 1H), 6.65 (d, *J* = 8.1 Hz, 2H), 5.65 (s, 2H). ^13^C NMR (151 MHz, DMSO-*d*_6_): δ 163.84, 149.32, 146.39, 145.72, 126.96, 124.47, 117.73, 116.77, 114.60, 98.52. Mass found (ESI+), 322.950 [M + H]^+^.

### 2-(3-(Benzo[*d*][1,3]dioxol-5-yl)-1-methyl-1*H*-pyrazol-5-yl)thiazole (**23**, **APT-8**)

NaH (60% mineral oil dispersion) (6 mg, 0.15 mmol, 1.3 equiv) was carefully added to a round-bottom flask. The flask was cooled to 0 °C and anhydrous THF (1.5 mL) was added. A solution of 2-(3-(benzo[*d*][1,3]dioxol-5-yl)-1*H*-pyrazol-5-yl)thiazole [**18**] (30 mg, 0.11 mmol, 1 equiv) in dry THF (1 mL) was added to the solution of NaH in THF at 0 °C, and the resulting mixture was stirred at room temperature for 30 min. A solution of MeI (0.021 mL, 0.17 mmol, 1.5 equiv) in THF (0.5 mL) was then added and the mixture was allowed to stir overnight at room temperature. The reaction was quenched by the addition of water (5 mL) followed by extraction with EA (5 mL × 3), drying with MgSO_4_, filtration, and concentration under reduced pressure. The crude product was then purified by flash chromatography (PE/EA, gradient 40–95% B) to afford the desired product as a white solid (18 mg, 56%). ^1^H NMR (600 MHz, DMSO-*d*_6_): δ 7.86 (d, *J* = 3.2 Hz, 1H), 7.67 (d, *J* = 3.2 Hz, 1H), 7.19 (d, *J* = 1.6 Hz, 1H), 7.09–7.02 (m, 2H), 6.82 (s, 1H), 6.11 (s, 2H), 3.88 (s, 3H). ^13^C NMR (151 MHz, DMSO-*d*_6_): δ 161.58, 148.29, 148.12, 145.28, 145.09, 143.59, 123.47, 123.22, 119.86, 109.48, 109.08, 103.75, 101.98, 38.31. Mass found (ESI+), 386.050 [M + H]^+^.

### 2-(3-(Benzo[*d*][1,3]dioxol-5-yl)-1-methyl-1*H*-pyrazol-5-yl)-4-bromothiazole (**24**, **APT-14**)

NaH (60% mineral oil dispersion) (15 mg, 0.37 mmol, 1.3 equiv) was carefully added to a round-bottom flask. The flask was cooled to 0 °C and THF was added (5 mL). A solution of 2-(3-(benzo[*d*][1,3]dioxol-5-yl)-1*H*-pyrazol-5-yl)-4-bromothiazole [**17**] (100 mg, 0.29 mmol, 1 equiv) in dry THF (3 mL) was added to the solution of NaH in THF at 0 °C, and the resulting mixture was stirred at room temperature for 30 min. A solution of MeI (0.054 mL, 0.63 mmol, 1.5 equiv) in THF (1 mL) was then added and the mixture was allowed to stir overnight at room temperature. The reaction was quenched by the addition of water (10 mL) followed by extraction with EA (10 mL × 3), drying with MgSO_4_, filtration, and concentration under reduced pressure. The crude product was then purified by flash chromatography (PE/EA, gradient 45–95% B) to afford the desired product as a white solid (78 mg, 74%). ^1^H NMR (600 MHz, CDCl_3_): δ 7.15 (s, 1H), 6.91–6.88 (m, 3H), 6.83 (s, 1H), 6.04 (s, 2H), 3.90 (s, 3H). ^13^C NMR (151 MHz, chloroform-*d*): δ 162.93, 148.35, 148.06, 145.32, 144.81, 125.35, 123.43, 122.83, 115.95, 109.12, 108.68, 104.29, 101.53, 37.84. Mass found (ESI+), 365.850 [M + H]^+^.

### 2-(3-(Benzo[*d*][1,3]dioxol-5-yl)-1*H*-pyrazol-5-yl)-5-bromothiazole (**25**, **APT-9**)

After solubilizing **15** (20 mg, 0.07 mmol, 1.0 equiv) in chloroform (2 mL), *N*-bromosuccinimide (NBS) (0.016 mL, 20 mg, 0.11 mmol, 1.5 equiv) was added to the solution with stirring. The mixture was stirred at 60 °C for 3 h, then H_2_O (3 mL) was added and extracted with EA (3 mL × 3), washed with brine, dried with anhydrous MgSO_4_, and filtered. The solvent was removed in vacuo, and the crude product was purified by silica-gel column chromatography (PE/EA, gradient 25–55% B) to afford the title compound as a white solid (13 mg, 50%). ^1^H NMR (600 MHz, DMSO-*d*_6_): δ 13.90 (s, 1H), 7.96 (s, 1H), 7.78 (d, 1H), 7.31–7.27 (m, 2H), 7.10 (s, 1H), 6.11 (s, 2H). ^13^C NMR (151 MHz, DMSO-*d*_6_): δ 159.69, 148.08, 147.61, 143.96, 143.41, 141.68, 122.01, 121.30, 120.26, 108.74, 107.93, 101.63, 89.03. Mass found (ESI+), 351.900 [M + H]^+^.

### 2-(3-(Benzo[*d*][1,3]dioxol-5-yl)-1*H*-pyrazol-5-yl)-4-fluorothiazole (**26**, **APT-10**)

**17** (70 mg, 0.20 mmol, 1.0 equiv) was dissolved in anhydrous THF (5 mL), and after cooling to −78 °C, *n*-BuLi 2.5 M in hexane (0.095 mL, 15.4 mg, 0.24 mmol, 1.2 equiv) was added and the mixture was stirred at −78 °C for 20 min. *N*-Fluorobenzenesulfonimide (95 mg, 0.30 mmol, 1.5 equiv) in THF (1 mL) was added at-78 °C and stirred at room temperature for 1 h. Sat. NH_4_Cl was added, and the mixture was extracted with EA, dried over anhydrous MgSO_4_, and filtrated, and the solvent evaporated under reduced pressure. The crude product was purified by flash chromatography using a silica-gel column (PE/EA, 3/1) to give the title compound as a white solid (18 mg, 0.06 mmol, 31%). ^1^H NMR (600 MHz, DMSO-*d*_6_): δ 13.63 (s, 1H), 7.43 (d, *J* = 1.8 Hz, 1H), 7.35 (dd, *J* = 8.1, 1.8 Hz, 1H), 7.15 (d, *J* = 4.4 Hz, 1H), 7.12 (s, 1H), 7.03 (d, *J* = 8.1 Hz, 1H), 6.08 (s, 2H). ^13^C NMR (151 MHz, DMSO): δ 162.02 (d, *J* = 296.9 Hz), 148.47, 148.38, 148.02, 144.57, 128.09, 125.19 (d, *J* = 13.3 Hz), 123.09, 119.83, 109.26, 106.38, 101.85, 99.75. Mass found (ESI+), 389.050, 291.00.

### Radiochemistry

#### Automated Synthesis of [^11^C]**APT-13**

[^11^C]CO_2_ was prepared by the bombardment of ^14^N_2_ target gas containing 1% O_2_ using a PETtrace 860 cyclotron (GE Healthcare, Chicago, IL, USA). [^11^C]methyl iodide ([^11^C]MeI) was produced via gas phase conversion^29^ using a TRACERlab FX MeI module (GE Healthcare) and transferred to a TRACERlab FX M module (GE Healthcare). [^11^C]methyl triflate ([^11^C]MeOTf) was generated by the reaction of [^11^C]MeI with a silver triflate column in an in-flow-through process at 200 °C under helium gas flow. The precursor **22** (1–2 mg, 3–6 μmoles) was dissolved in 500 μL of 2-butanone. No carrier added [^11^C]MeOTf was bubbled through the reaction solution, previously cooled to −20 °C, and the mixture was heated to 75 °C for 2 min. After reaching room temperature, the solution was diluted with 1.5 mL of H_2_O and transferred to the HPLC system. The crude product was purified by semipreparative HPLC on a Supercosil LC-ABZ+, 5 μm, 250 × 10 mm column and eluted with an isocratic flow of 55% ACN in water at a flow rate of 6 mL/min with a retention time of 8.3 ± 0.4 min (Figure S1). Chromatograms were registered using a UV detector (254 nm) and a NaI radioactivity detector. The HPLC fraction containing the purified product was diluted with 50 mL of water and subsequently loaded onto a Sep-Pak Plus Light C18 cartridge (Waters Corporation, Milford, MA, USA), previously conditioned with 10 mL of ethanol and 10 mL of water. The cartridge was washed with 5 mL of water, and the product was eluted with 0.5 mL of ethanol and formulated with the addition of 5 mL of phosphate-buffered saline (PBS; Thermo Fisher Scientific, Waltham, MA, USA). Radiochemical and chemical purity of the radiolabeled compound, as well as carrier content for calculation of molar radioactivity, were determined by analytical radio-HPLC on a 1260 Infinity HPLC-System (Agilent Technologies, Santa Clara, CA, USA) equipped with a NaI (Tl) scintillator system. Separation was achieved on a Luna C18(2), 5 μm, 250 × 4.6 mm column with 35% ACN in 0.1% aqueous trifluoracetic acid (TFA) as mobile phase and at a flow rate of 1.5 mL/min in 10 min. The radiolabeled product was eluted at a retention time of 5.9 ± 0.2 min (Figure S2). At the end of the synthesis (EOS), the radiochemical yield (% RCY) was 13.5 ± 1.8% (from trapped [^11^C]MeOTf to formulated radioligand) and the *A*_m_ was 119.6 ± 33.8 GBq/μmol.

#### Synthesis of [^3^H]MODAG-001

MODAG-001 was tritiated by RC Tritec AG, Teufen Switzerland. The *A*_m_ was 2.9 GBq/μmol, and the radiochemical purity was >99%, as determined via high-performance liquid chromatography (HPLC).

### Biological Evaluation

#### Calculation of BBB Score

All compounds properties were calculated using Chemicalize software (ChemAxon, Budapest, Hungary) and entered into the Excel sheet provided in the literature for the calculation of BBB score.^22^ All values are reported in the Supporting Information (Table S1).

#### Preparation of α-Syn and Aβ_1–42_ Fibrils

α-Syn fibrils were generated as the previously reported method.^30^ The generation of Aβ_1–42_ fibril was adapted from Bagchi et al. and previously described by Kuebler et al.^16,31^ Synthetic lyophilized human Aβ_1–42_ peptide with >90% purity purchased from EMC Microcollections GmbH (Tuebingen, Germany) was dissolved in dimethyl sulfoxide (DMSO; 5 mg in 221.5 μL). Deionized water (4.1 mL) and 1 M Tris–HCl (111 μL, pH 7.6) were added to obtain a final monomeric concentration of 250 μM. Aggregation was induced by incubating the monomers in Eppendorf Thermomixer at 37 °C with shaking at 800 rpm for 72 h. Generated fibrils were sonicated for 3 min in a water bath (Elmasonic S 60H, Elma Schmidbauer GmbH, Singen, Germany). The final products were aliquoted, frozen on dry ice, and stored at −80 °C until use. Aβ_1–42_ fibril quality was evaluated via thioflavin (ThT) fluorescence and radioligand saturation assay with [^3^H]PiB and [^3^H]MODAG-001.

#### In Vitro Fibrils Binding Experiments

The dissociation constant (*K*_d_) values of [^3^H]MODAG–001 for human recombinant α-syn and synthetic human Aβ_1–42_ fibrils have previously been determined using saturation binding assays.^30^ To determine the relative binding affinity (inhibition constant, *K*_i_) of test compounds, competition binding assays of [^3^H]MODAG-001 were performed. Test compounds were dissolved in DMSO to a stock concentration of 1 mM, which resulted in ≤1% DMSO concentration in the final assay. Human recombinant α-syn (50 nM) and synthetic human Aβ_1–42_ fibrils (1 μM) diluted in PBS (Gibco DPBS, no calcium, no magnesium, Thermo Fisher Scientific, Waltham, MA, USA) were incubated with increasing concentrations of test compounds (5 pM to 10 μM) against 1 nM [^3^H]MODAG–001 in 30 mM Tris–HCl, 0.1% bovine serum albumin, and 0.05% Tween20 in 96-well micro test low-binding plates (ratiolab GmbH, Dreieich, Germany) to a total volume of 200 μL/well. Plates were incubated on a shaker (MaxQ 6000, Thermo Fisher Scientific Inc., Marietta, OH, USA) at 50 rpm for 2 h at 37 °C, covered by removable sealing tapes (PerkinElmer, Waltham, MA, USA). Vacuum filtration and read-out were performed as previously reported (Kuebler, Buss et al. 2021). Briefly, a glass fiber Filtermat B (PerkinElmer, Waltham, MA, USA) incubated with 0.5% polyethylenimine (PEI; Sigma-Aldrich Chemie GmbH, Taufkirchen, Germany) for 30 min at 4 °C before filter harvesting was used with a FilterMate Harvester (PerkinElmer, Waltham, MA, USA). Melt-on scintillator sheets (MeltiLex B/HS, PerkinElmer, Waltham, MA, USA) were molten onto dried filters. Accumulation of tritium was counted in a liquid scintillation Wallac MicroBeta TriLux counter (PerkinElmer, Waltham, MA, USA) at 2 min per well. Radioactivity in counts per minute for each concentration of the test compound was normalized to the radioactivity obtained in the absence of the test compound for conversion to percentage binding of the reference tracer. The resulting values were plotted against increasing concentrations of the test compounds. Data points were fitted using nonlinear regression analysis in GraphPad Prism (GraphPad Software, Inc., Version 8.4.0, La Jolla, CA, USA). The binding curve for [^3^H]MODAG-001 saturation assay on α-syn fibrils is reported in the Supporting Information (Figure S6).

#### Log*D* Determination of **APT-13**

The logD was calculated experimentally by adding [^11^C]**APT-13** (1.5 MBq) to a 1.5 mL Eppendorf vial containing 500 μL of octanol (OCT) and 500 μL of PBS. The vial was vigorously vortexed and then, to achieve effective phase separation, centrifuged. Aliquots of 350 μL were collected from both OCT and PBS phases. The OCT sample was further washed with 350 μL of PBS; the phases mixture was shaken and centrifuged, and 200 μL of both phases were taken into new reaction tubes. The amount of radioactivity in each sample was quantified using a γ-counter. The log OCT/PBS values were calculated from the mean of *n* = 3 determinations (Table S3).

#### Serum Stability Assay

Human serum was obtained from human male AB plasma, USA origin, sterile-filtered (Sigma-Aldrich, St. Louis, MO, USA).

The tracer formulation was mixed with an equal volume of human serum (250 μL) and incubated into a thermo-shaker at 37 °C, 300 rpm. At different time points (0, 15, 30, 60, and 90 min), 70 μL samples were drawn and mixed with ice-cold acetonitrile (1:1 dilution) and quickly vortexed. The dilute sample was incubated for 2 min on ice and centrifuged (MiniSpin centrifuge, Eppendorf) for 2 min at 13,400 rpm. An aliquot of the supernatant (120 μL) was analyzed by analytical radio-HPLC on a Luna C18(2), 5 μm, 250 × 4.6 mm column with 35% ACN in 0.1% aqueous TFA as mobile phase and at a flow rate of 1.5 mL/min in 10 min. The radiolabeled product was eluted at a retention time of 5.9 ± 0.2 min (Figure S5).

#### In Vivo PET/MR Imaging

Mouse experiments were conducted in accordance with the European directives on the protection and use of laboratory animals (Council Directive 2010/63/UE) and with the German animal protection law and with the approval of the local authorities (Regierungspräsidium Tuebingen (Germany), R3/19G). Healthy wild-type female C57BL/6J mice were purchased from Charles River Laboratories (Sulzfeld, Germany).

To evaluate the pharmacokinetics of [^11^C]**APT-13**, PET and subsequent MRI scans were performed in healthy mice (female, 21 weeks old, C57BL/6J) to determine brain uptake and washout as well as organ uptake of the liver, lungs, heart, kidneys, and brain. Scans were performed in a small animal Inveon PET scanner (Siemens Healthcare, Erlangen, Germany) and a 7T small animal MRI scanner (Bruker BioSpin GmbH, Ettlingen, Germany). The mice (*n* = 3) were anesthetized with 1.5% isoflurane evaporated in 100% oxygen at a flow of 0.8 L/min, and a catheter was placed in the tail vein. The body temperature was kept at 37 °C by using a feedback temperature control unit for a heat pump that was connected to the PET bed. Five seconds after the start of the PET acquisition, 15.2 ± 0.5 MBq (410 ± 13 μCi) of [^11^C]**APT-13** was injected intravenously. PET data were acquired in a dynamic scan for 60 min plus an extra 13 min of transmission measurement with a ^57^Co-source for attenuation correction. Subsequently, mice were placed in the same bed position in the MRI, and an anatomical scan was performed using a Turbo RARE T2 sequence. For analysis, the 60 min of acquired PET data were divided into 39-time frames (12 × 5, 6 × 10, 6 × 30, 5 × 60, and 10 × 300 s) and reconstructed using the OSEM3D reconstruction algorithm. The PET scans were coregistered to the whole-body MRI scan using PMOD software (version 4.203; PMOD Technologies, Zürich, Switzerland), and the volume of interest (VOI) of the different organs (liver, lungs, heart, kidney, and brain) were hand-drawn according to the anatomical localization. VOI were selected from the mouse brain atlas in PMOD, time activity curves were extracted, and SUV were calculated by normalizing to the injected activity and animal weight.

## Supporting Information Available

Semipreparative HPLC and radio HPLC chromatograms, calibration curve for the calculation of the molar activity, additional information on the biological evaluation, serum stability assay radio HPLC chromatograms, log*D* determination results, ^1^H and ^13^C NMR spectra, and HPLC-MS chromatograms of the tested compounds and the radiolabeling precursor (PDF)

## Abbreviations

^11^C: carbon-11
Aβ: amyloid β
ACN: acetonitrile
AD: Alzheimer’s disease
*A*_m_: molar activity
α-syn: α-synuclein
APT: arylpyrazolethiazole
AR: autoradiography
BBB: blood–brain barrier
CNS: central nervous system
CNS PET MPO: central nervous system positron emission tomography multiparameter optimization
DLB: dementia with Lewy bodies
dp: data points
DPP: diphenylpyrazole
FA: formic acid
HBD: hydrogen-bond donor
*K*_i_: inhibition constant
MAO-B: monoamine oxidase-B
[^11^C]MeOTf: [^11^C]methyl triflate
MSA: multiple system atrophy
NMR: nuclear magnetic resonance
NDB: nondisplaceable binding
NSB: nonspecific binding
PD: Parkinson’s disease
PET: positron emission tomography
RCY: radiochemical yield
SAR: structure–activity relationship
SEM: standard error of the mean
SUV: standardized uptake value
ThT: thioflavin T, tPSA, topological polar surface area
VOI: volume of interest

## References

[ref1] Spillantini M. G.; Schmidt M. L.; Lee V. M. Y.; Trojanowski J. Q.; Jakes R.; Goedert M. α-Synuclein in Lewy bodies. Nature 1997, 388 (6645), 839–840. 10.1038/42166. 9278044

[ref2] Gelb D. J.; Oliver E.; Gilman S. Diagnostic criteria for Parkinson disease. Arch. Neurol. 1999, 56 (1), 33–39. 10.1001/archneur.56.1.33. 9923759

[ref3] Del Tredici K.; Braak H. Review: Sporadic Parkinson’s disease: development and distribution of α-synuclein pathology. Neuropathol. Appl. Neurobiol. 2016, 42 (1), 33–50. 10.1111/nan.12298. 26662475

[ref4] Jucker M.; Walker L. C. Self-propagation of pathogenic protein aggregates in neurodegenerative diseases. Nature 2013, 501 (7465), 45–51. 10.1038/nature12481. 24005412 PMC3963807

[ref5] Fayyad M.; Salim S.; Majbour N.; Erskine D.; Stoops E.; Mollenhauer B.; El-Agnaf O. M. A. Parkinson’s disease biomarkers based on α-synuclein. J. Neurochem. 2019, 150 (5), 626–636. 10.1111/jnc.14809. 31265130

[ref6] Mathis C. A.; Lopresti B. J.; Ikonomovic M. D.; Klunk W. E. Small-molecule PET Tracers for Imaging Proteinopathies. Semin. Nucl. Med. 2017, 47 (5), 553–575. 10.1053/j.semnuclmed.2017.06.003. 28826526 PMC5657567

[ref7] Mathis C. A.; Lopresti B. J.; Ikonomovic M. D.; Klunk W. E. Small-molecule PET Tracers for Imaging Proteinopathies. Semin. Nucl. Med. 2017, 47 (5), 553–575. 10.1053/j.semnuclmed.2017.06.003. 28826526 PMC5657567

[ref8] McCluskey S. P.; Plisson C.; Rabiner E. A.; Howes O. Advances in CNS PET: the state-of-the-art for new imaging targets for pathophysiology and drug development. Eur. J. Nucl. Med. Mol. Imaging 2020, 47 (2), 451–489. 10.1007/s00259-019-04488-0. 31541283 PMC6974496

[ref9] Pike V. W. Considerations in the development of reversibly binding PET radioligands for brain imaging. Curr. Med. Chem. 2016, 23 (18), 1818–1869. 10.2174/0929867323666160418114826. 27087244 PMC5579844

[ref10] Korat S. ˇ.; Bidesi N. S. R.; Bonanno F.; Di Nanni A.; Hoàng A. N. N.; Herfert K.; Maurer A.; Battisti U. M.; Bowden G. D.; Thonon D.; et al. Alpha-Synuclein PET Tracer Development—An Overview about Current Efforts. Pharmaceuticals 2021, 14 (9), 847 10.3390/ph14090847. 34577548 PMC8466155

[ref11] Smith R.; Capotosti F.; Schain M.; Ohlsson T.; Vokali E.; Molette J.; Touilloux T.; Hliva V.; Dimitrakopoulos I. K.; Puschmann A.; et al. The α-synuclein PET tracer [18F] ACI-12589 distinguishes multiple system atrophy from other neurodegenerative diseases. Nat. Commun. 2023, 14 (1), 6750 10.1038/s41467-023-42305-3. 37891183 PMC10611796

[ref12] Xiang J.; Tao Y.; Xia Y.; Luo S.; Zhao Q.; Li B.; Zhang X.; Sun Y.; Xia W.; Zhang M.; et al. Development of an α-synuclein positron emission tomography tracer for imaging synucleinopathies. Cell 2023, 186 (16), 3350–3367.e19. 10.1016/j.cell.2023.06.004. 37421950 PMC10527432

[ref13] Saw R. S., Buss S., Schmidt F., Ryazanov S., Leonov A., Kuebler L., Bleher D., Papadopoulos I., Roeben B., Schmidt F., Reimold M., Bonanno F., Grotegerd A.-K., Ruf V., Dahl B., Sandiego C., Henry K., Fehrenbacher B., Schaller M., Kahle P., Gasser T., Brockmann K., Reischl G., Fougère C. l., Pichler B., Maurer A., Griesinger C., Giese A., Herfert K. 11C MODAG 005 – a novel PET tracer targeting alpha-synuclein aggregates in the brain, 2024; PREPRINT (Version 1) available at Research Square 10.21203/rs.3.rs-2189800/v1.

[ref14] Kaide S.; Watanabe H.; Iikuni S.; Hasegawa M.; Ono M. Synthesis and Evaluation of 18F-Labeled Chalcone Analogue for Detection of α-Synuclein Aggregates in the Brain Using the Mouse Model. ACS Chem. Neurosci. 2022, 13 (20), 2982–2990. 10.1021/acschemneuro.2c00473. 36197745

[ref15] Wagner J.; Ryazanov S.; Leonov A.; Levin J.; Shi S.; Schmidt F.; Prix C.; Pan-Montojo F.; Bertsch U.; Mitteregger-Kretzschmar G.; et al. Anle138b: a novel oligomer modulator for disease-modifying therapy of neurodegenerative diseases such as prion and Parkinson’s disease. Acta Neuropathol. 2013, 125, 795–813. 10.1007/s00401-013-1114-9. 23604588 PMC3661926

[ref16] Kuebler L.; Buss S.; Leonov A.; Ryazanov S.; Schmidt F.; Maurer A.; Weckbecker D.; Landau A. M.; Lillethorup T. P.; Bleher D.; et al. [11 C] MODAG-001—towards a PET tracer targeting α-synuclein aggregates. Eur. J. Nucl. Med. Mol. Imaging 2021, 48, 1759–1772. 10.1007/s00259-020-05133-x. 33369690 PMC8113290

[ref17] Uzuegbunam B. C.; Li J.; Paslawski W.; Weber W.; Svenningsson P.; Ågren H.; Yousefi B. H. Toward Novel [18F] Fluorine-Labeled Radiotracers for the Imaging of α-Synuclein Fibrils. Front. Aging Neurosci. 2022, 14, 830704 10.3389/fnagi.2022.830704. 35572127 PMC9099256

[ref18] Antonschmidt L.; Matthes D.; Dervişoğlu R.; Frieg B.; Dienemann C.; Leonov A.; Nimerovsky E.; Sant V.; Ryazanov S.; Giese A.; et al. The clinical drug candidate anle138b binds in a cavity of lipidic α-synuclein fibrils. Nat. Commun. 2022, 13 (1), 5385 10.1038/s41467-022-32797-w. 36104315 PMC9474542

[ref19] Ryan P.; Xu M.; Jahan K.; Davey A. K.; Bharatam P. V.; Anoopkumar-Dukie S.; Kassiou M.; Mellick G. D.; Rudrawar S. Novel Furan-2-yl-1H-pyrazoles Possess Inhibitory Activity against α-Synuclein Aggregation. ACS Chem. Neurosci. 2020, 11 (15), 2303–2315. 10.1021/acschemneuro.0c00252. 32551538

[ref20] Hsieh C. J.; Xu K.; Lee I.; Graham T. J. A.; Tu Z.; Dhavale D.; Kotzbauer P.; Mach R. H. Chalcones and Five-Membered Heterocyclic Isosteres Bind to Alpha Synuclein Fibrils in Vitro. ACS Omega 2018, 3 (4), 4486–4493. 10.1021/acsomega.7b01897. 30221226 PMC6130786

[ref21] Gupta M.; Lee H. J.; Barden C. J.; Weaver D. F. The Blood–Brain Barrier (BBB) Score. J. Med. Chem. 2019, 62 (21), 9824–9836. 10.1021/acs.jmedchem.9b01220. 31603678

[ref22] Zhang X.; Kang J.; Niu P.; Wu J.; Yu W.; Chang J. I_2_-Mediated Oxidative C–N Bond Formation for Metal-Free One-Pot Synthesis of Di-Tri-and Tetrasubstituted Pyrazoles from α,β-Unsaturated Aldehydes/Ketones and Hydrazines. J. Org. Chem. 2014, 79 (21), 10170–10178. 10.1021/jo501844x. 25279429

[ref23] Hsieh C. J.; Ferrie J. J.; Xu K.; Lee I.; Graham T. J. A.; Tu Z.; Yu J.; Dhavale D.; Kotzbauer P.; Petersson E. J.; et al. Alpha Synuclein Fibrils Contain Multiple Binding Sites for Small Molecules. ACS Chem. Neurosci. 2018, 9 (11), 2521–2527. 10.1021/acschemneuro.8b00177. 29750499 PMC6736640

[ref24] Wilcken R.; Zimmermann M. O.; Lange A.; Joerger A. C.; Boeckler F. M. Principles and applications of halogen bonding in medicinal chemistry and chemical biology. J. Med. Chem. 2013, 56 (4), 1363–1388. 10.1021/jm3012068. 23145854

[ref25] Heise H.; Celej M. S.; Becker S.; Riedel D.; Pelah A.; Kumar A.; Jovin T. M.; Baldus M. Solid-State NMR Reveals Structural Differences between Fibrils of Wild-Type and Disease-Related A53T Mutant α-Synuclein. J. Mol. Biol. 2008, 380 (3), 444–450. 10.1016/j.jmb.2008.05.026. 18539297

[ref26] Yang Y.; Shi Y.; Schweighauser M.; Zhang X.; Kotecha A.; Murzin A. G.; Garringer H. J.; Cullinane P. W.; Saito Y.; Foroud T.; et al. Structures of α-synuclein filaments from human brains with Lewy pathology. Nature 2022, 610 (7933), 791–795. 10.1038/s41586-022-05319-3. 36108674 PMC7613749

[ref27] Li Y.; Zhao C.; Luo F.; Liu Z.; Gui X.; Luo Z.; Zhang X.; Li D.; Liu C.; Li X. Amyloid fibril structure of α-synuclein determined by cryo-electron microscopy. Cell Research 2018, 28 (9), 897–903. 10.1038/s41422-018-0075-x. 30065316 PMC6123497

[ref28] Venkatesan P.; Maruthavanan T. Piperidine-mediated synthesis of thiazolyl chalcones and their derivatives as potent antimicrobial agents. Nat. Prod. Res. 2012, 26 (3), 223–234. 10.1080/14786419.2010.536161. 21834630

[ref29] Larsen P.; Ulin J.; Dahlstrøm K.; Jensen M. Synthesis of [11C]iodomethane by iodination of [11C]methane. Appl. Radiat. Isot. 1997, 48 (2), 153–157. 10.1016/S0969-8043(96)00177-7.

[ref30] Di Nanni A.; Saw R. S.; Bowden G. D.; Bidesi N. S. R.; Bjerregaard-Andersen K.; Korat S. ˇ.; Herth M. M.; Pichler B. J.; Herfert K.; Maurer A. The Structural Combination of SIL and MODAG Scaffolds Fails to Enhance Binding to α-Synuclein but Reveals Promising Affinity to Amyloid β. Molecules 2023, 28 (10), 4001 10.3390/molecules28104001. 37241742 PMC10224536

[ref31] Bagchi D. P.; Yu L.; Perlmutter J. S.; Xu J.; Mach R. H.; Tu Z.; Kotzbauer P. T. Binding of the radioligand SIL23 to α-synuclein fibrils in Parkinson disease brain tissue establishes feasibility and screening approaches for developing a Parkinson disease imaging agent. PLoS One 2013, 8 (2), e55031 10.1371/journal.pone.0055031. 23405108 PMC3566091

