# Supplementary material for: Advancing Parkinson’s Disease Diagnostics: The Potential of Arylpyrazolethiazole Derivatives for Imaging α-Synuclein Aggregates
Source: ACS Omega. 2024 May 30;9(23):24774–88. doi: 10.1021/acsomega.4c01301 (PMC11170759; doi:10.1021/acsomega.4c01301)
Supplement: Supplementary file 1 — ao4c01301_si_001.pdf [file ao4c01301_si_001.pdf]

Supplementary Materials

Advancing Parkinson's Disease Diagnostics: the Potential of Arylpyrazolethiazole Derivatives for Imaging α-Synuclein Aggregates

Federica Bonanno[1], Ran Sing Saw[1], Daniel Bleher[1], Ioannis Papadopoulos[1], Gregory D. Bowden[1,2], Kaare Bjerregaard-Andersen[3], Albert D. Windhorst[3], Bernd J. Pichler[1,2], Kristina Herfert[1], Andreas Maurer[1,2]

[1] *Werner Siemens Imaging Center, Department of Preclinical Imaging & Radiopharmacy, Eberhard Karls University Tübingen, Röntgenweg 13, 72076 Tübingen, Germany*

[2] *Cluster of Excellence iFIT (EXC 2180) "Image-Guided and Functionally Instructed Tumor Therapies", Eberhard Karls University Tübingen, Röntgenweg 11, 72076 Tübingen, Germany*

[3] *Department of Antibody Engineering and Biochemistry, H. Lundbeck A/S, Ottiliavej 9, 2500 Copenhagen, Denmark.*

[4] *Department of Radiology and Nuclear Medicine, Amsterdam UMC, Vrije Universiteit Amsterdam, De Boelelaan 1085c, 1081 HV Amsterdam, the Netherlands*

Table of Contents

Table of Contents 1

Radiochemistry 2

1.1 Radiosynthesis 2

1.2 Calibration curve 2

1.3 Quality control 4

2. Biological evaluation 5

2.1 Serum stability assay 6

2.2 Binding assay curves 7

1. ^{1}H and ^{13}C NMR spectra of compounds 12

4. HPLC-MS chromatograms of APT-1-14 38

Radiochemistry

1.1 Radiosynthesis

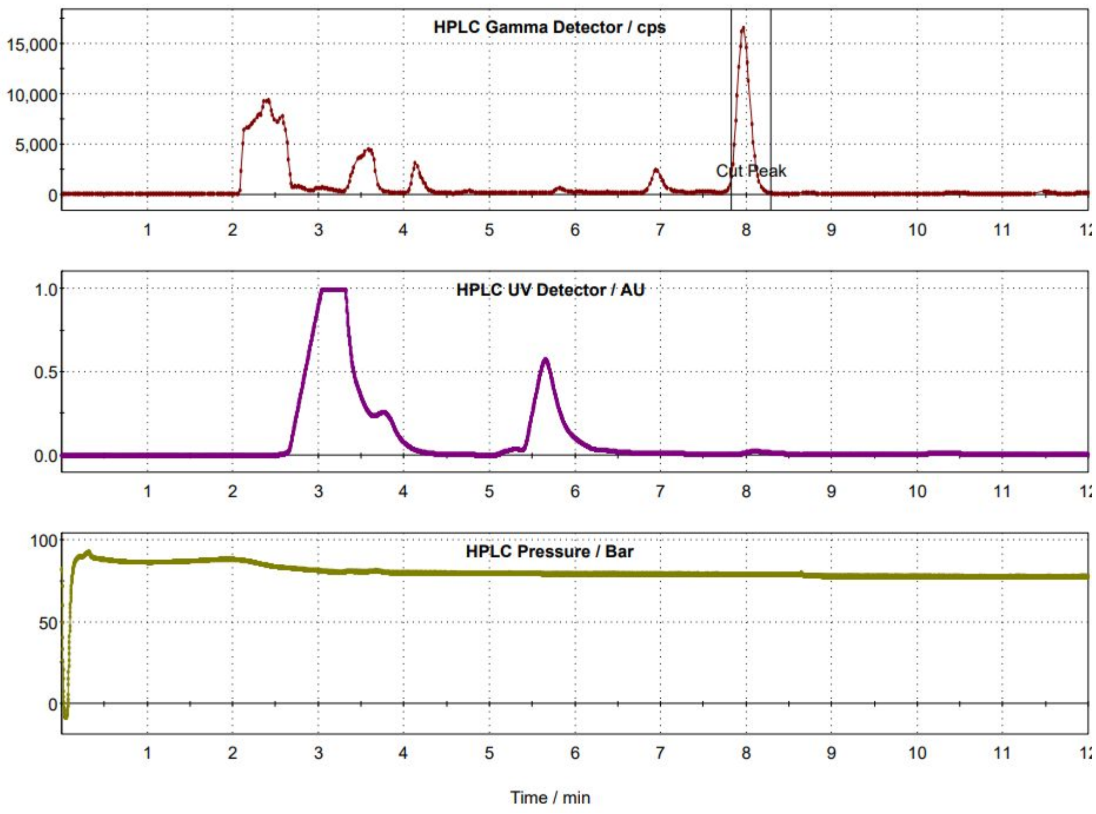

Figure S1. Example of semipreparative HPLC chromatogram of [^{11}C]APT-13 (radioactivity detector chromatogram, UV Sig. 254 and system pressure) using a Supercosil ABZ+, 5 µm, 250 × 10 mm column, isocratic method: 55% ACN in water, 6 mL/min. Retention time: [^{11}C]APT-13 ≈ 8 min.

1.2 Calibration curve

<u>Preparation of stock solution</u>: Dissolve 1 mg of APT-13 in 1mL of ACN/water (QC eluent)

<u>Dilution series</u>:

1. 1/10: 100ul Stock sol. + 900 ul QC-eluent
2. 1/20: 500ul Dil. 1 + 500 ul QC-eluent
3. 1/100: 200ul Dil. 2 + 800 ul QC-eluent
4. 1/200: 500 ul Dil. 3 + 500 ul QC-eluent

A

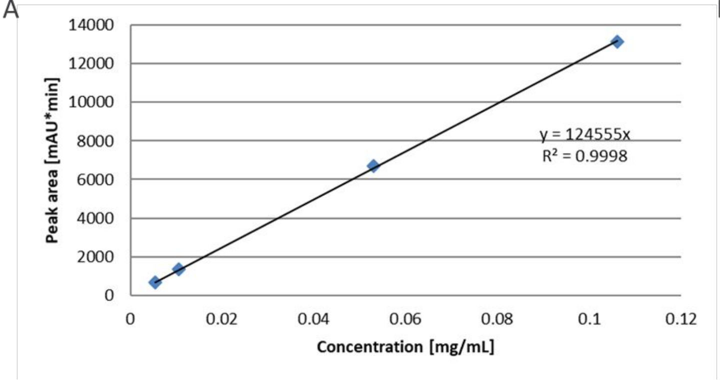

B

Dil. series	Concentration in mg/mL	Peak area in mAU*min	t_R in min
Stock sol.	1.06 mg	58339	5.5
1	0.106	13138	5.9
2	0.053	6722	5.9
3	0.0106	1358	5.9
4	0.0053	675	5.9

Figure S2. A. Calibration curve for the calculation of the molar activity. B. Table with integration area of peaks of the dilution series 1-4 at δ UV Sig. 254. t_R = retention time.

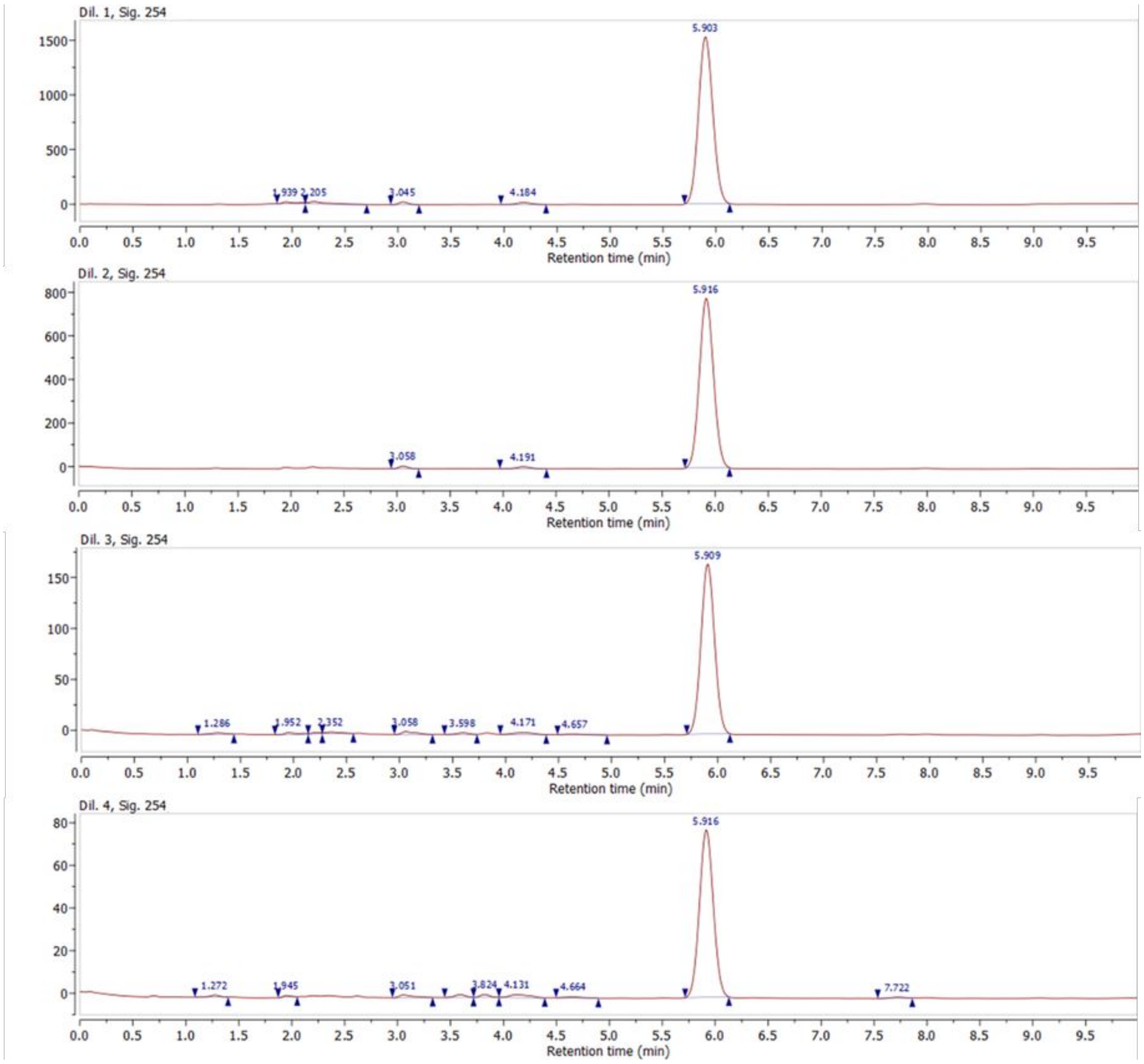

Figure S3. Chromatograms at UV Sig. 254 of the dilution series using a Luna column 5 µm C18(2) 100 Å 250 x 4.6 mm, isocratic method: 35% ACN in 0,1% TFA, 1,5 mL/min. Retention time: APT-13 ≈ 5.903-5.916.

1.3 Quality control

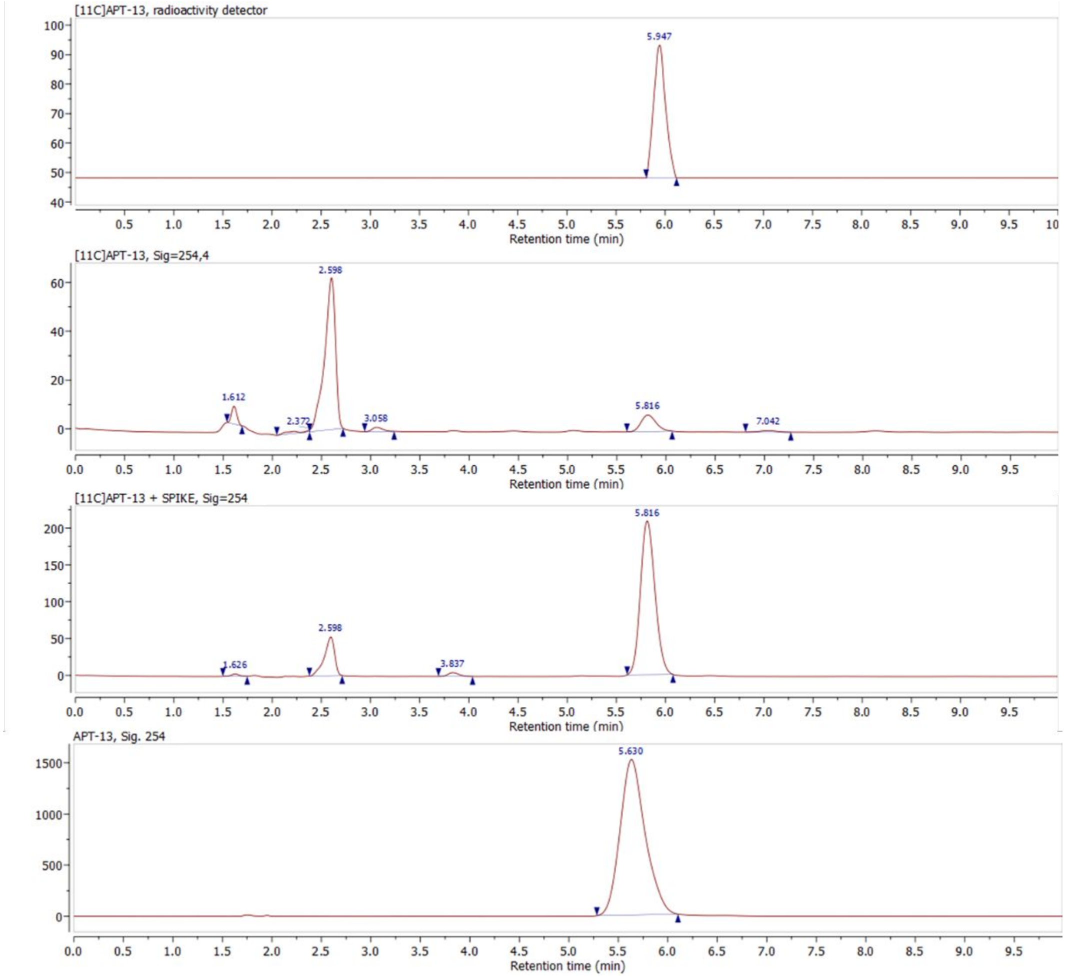

Figure S4. Example of analytical quality control (QC) HPLC of [^{11}C]APT-13 (radioactivity detector chromatogram, Sig. 254; Sig. 346 and with the addition of spike at Sig. 254) and its non-radioactive reference compound (chromatogram at Sig. 254) using a Luna column 5 µm C18(2) 100 Å 250 x 4.6 mm, isocratic method: 35% ACN in 0,1% TFA, 1,5 mL/min. Retention time: [^{11}C]APT-13 ≈ 5.947, APT-13 ≈ 5.816 min.

2. Biological evaluation

Table S1. Chemical properties, BBB score and CNS MPO for all APT-compounds calculated with Chemicalize. n.d. = not determined

#	cLogP	tPSA (Å^2)	MW (g/mol)	Aromatic rings	Heavy atoms	Hydrogen bond acceptors	Hydrogen bond donors	BBB score
**APT-1**	2.54	60.03	271.29	3	19	4	1	4.59
**APT-2**	1.82	73.17	255.23	3	19	4	1	4.33
**APT-3**	2.55	48,42	259.28	2	18	4	0	4.76
**APT-4**	3.51	60,03	350,19	3	20	4	1	4.70
**APT-5**	3.02	44.81	270.35	3	19	3	1	5.00
**APT-6**	2.59	53.60	288.34	3	20	2	2	4.78
**APT-7**	2.39	16.13	256.33	3	18	3	2	4.62
**APT-8**	2.68	49.19	285.32	3	20	4	0	4.47
**APT-9**	3.47	60.03	350.19	3	20	4	1	4.78
**APT-10**	3.07	60.03	289.28	3	20	4	1	4.57
**APT-11**	2.4	41.99	244.31	2	17	3	1	5.01
**APT-12**	3.37	41.99	323.21	2	18	3	1	4.93
**APT-13**	3.36	53.60	335.22	3	19	3	2	4.80
**APT-14**	3.61	49.19	364.22	3	21	5	0	4.41

2.1 Serum stability assay

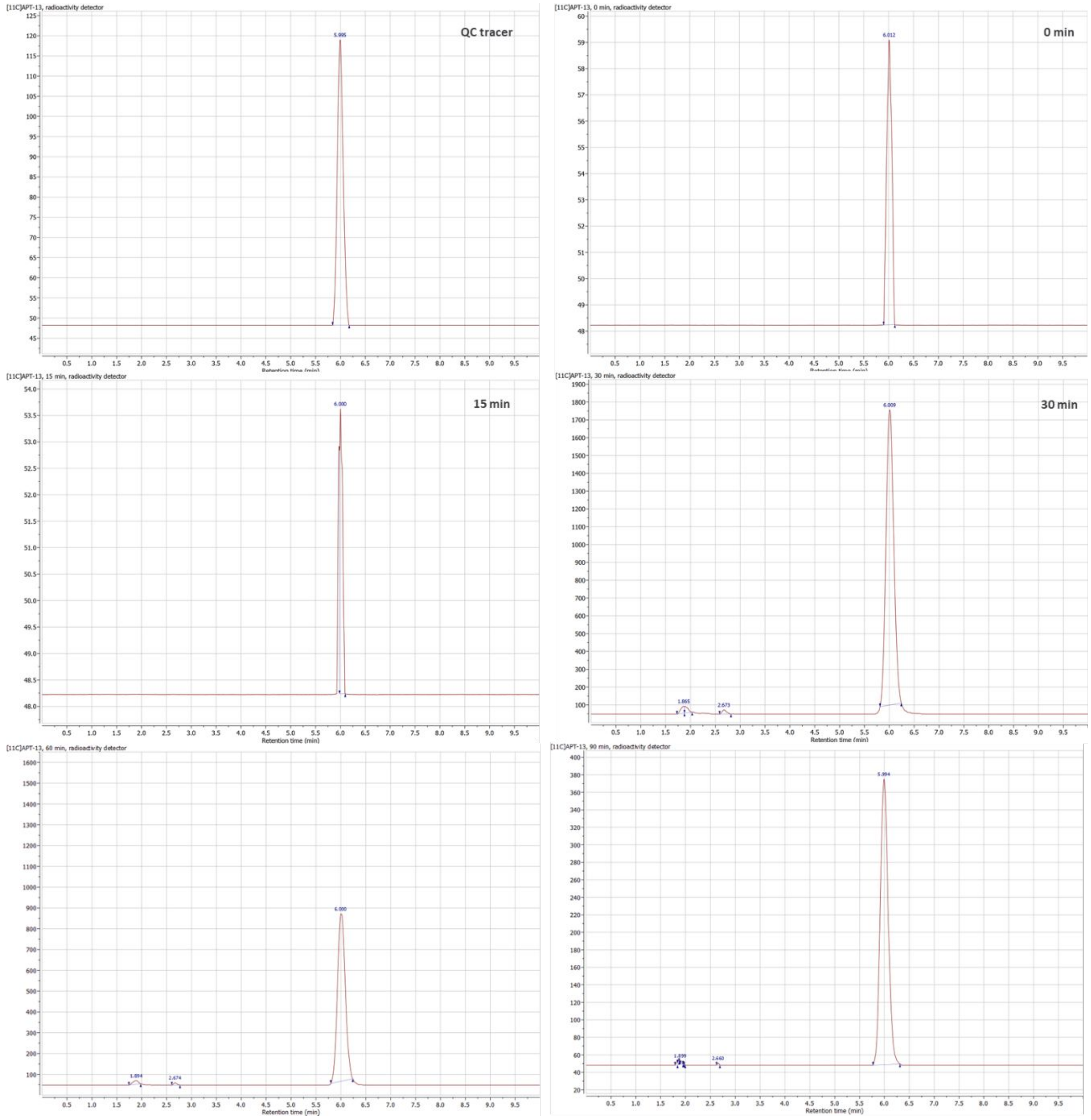

Figure S5. Radioactivity detector chromatograms of the serum stability assay at 0, 15, 60 and 90 minutes.

2.2 Binding assay curves

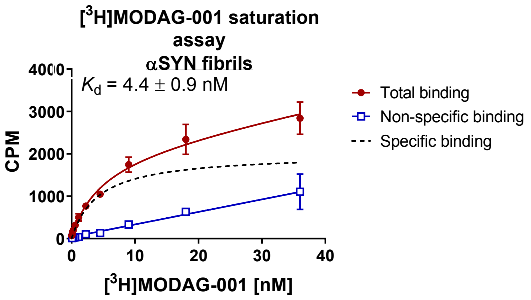

Figure S6. Binding curve for [^{3}H]MODAG-001 saturation assay on α-synuclein (α-syn) fibrils. The saturation binding assay curve for [^{3}H]MODAG-001 on amyloid-β ($A\beta_{1-42}$) fibrils (Di Nanni, Saw et al. 2023) was already reported by Di Nanni and colleagues (Di Nanni, Saw et al. 2023).

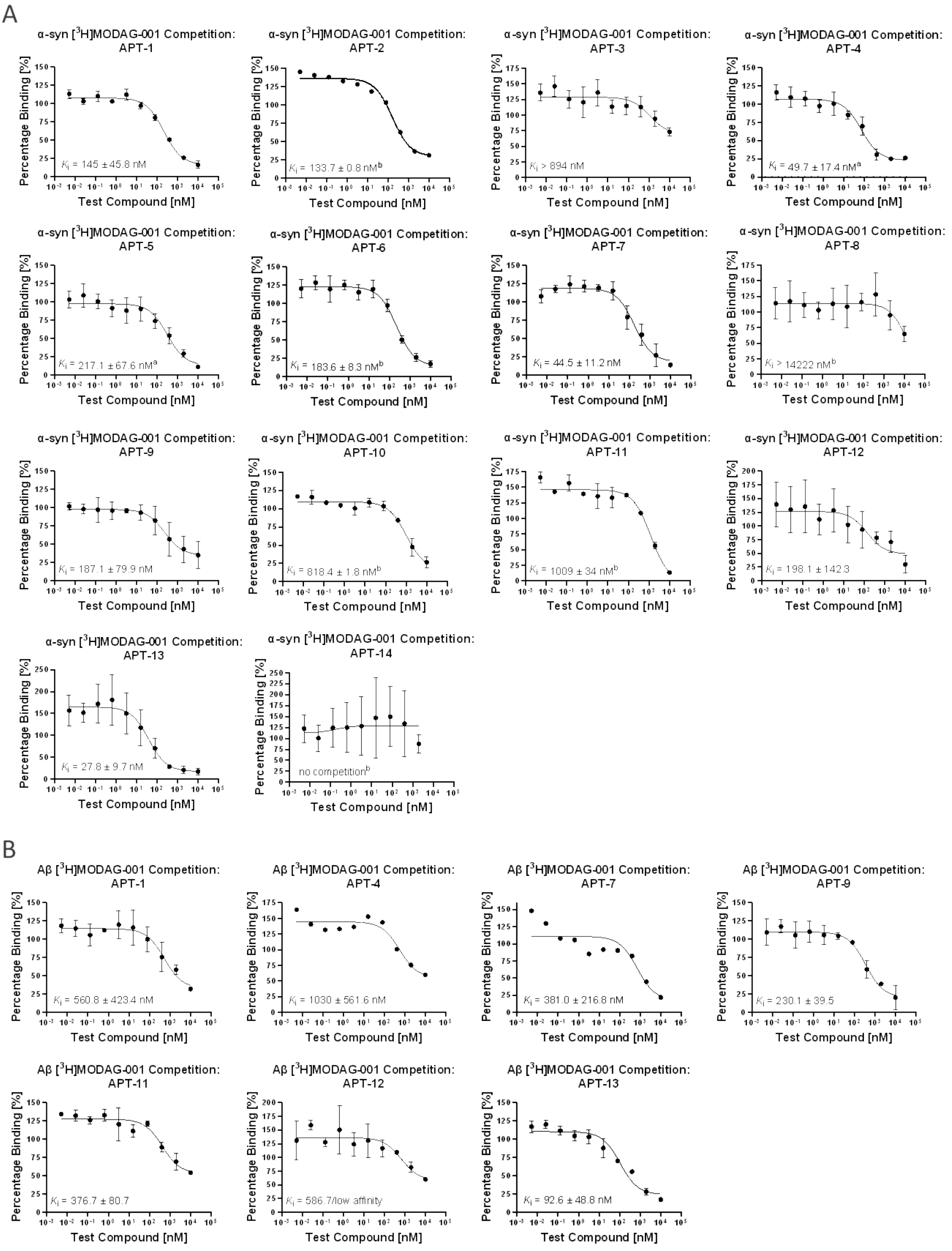

Figure S7. Binding curves for [^{3}H]MODAG-001 competition assay on α-syn for all APT compounds (A) and on $A\beta_{1-42}$ fibrils for selected APT compounds (B). K_i data three data points (dp) for α-syn, two dp for Aβ; [a]dp = 4, [b]dp =2.

Table S2. K_i value for APT library on α-syn and Aβ. K_i data reported in MEAN K_i + SEM, three data points (dp) for α-syn, two dp for Aβ; [a]dp = 4, [b]dp =2; n.d. = not determined

Competition binding assay on [^{3}H]MODAG-001				
	**APT-n**	**Chemical structure**	**K_i α-syn (nM)**	**K_i $A\beta_{1-42}$ (nM)**
**Group A**	APT-5	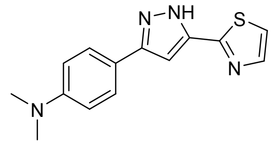	217 ± 68	n.d.
	APT-6	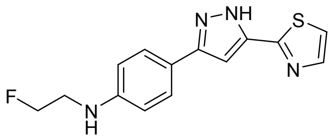	184 ± 8	n.d.
	APT-7	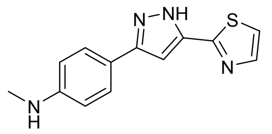	45 ± 11	381 ± 217
**Group B**	APT-1	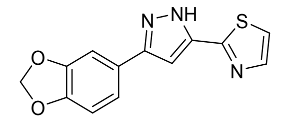	145 ± 46	561 ± 423
	APT-3	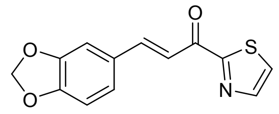	> 894	n.d.
	APT-8	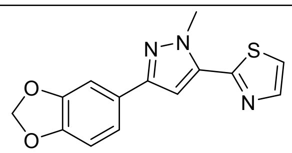	> 14222	n.d.
**Group C**	APT-2	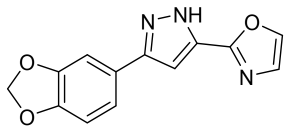	134 ± 0.8	n.d.
	APT-4	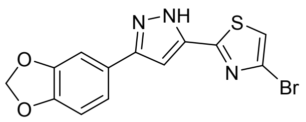	50 ± 17	1030 ± 562

	APT-9	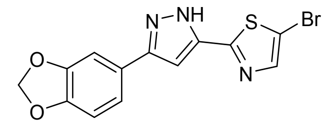	187 ± 80	230 ± 40
	APT-10	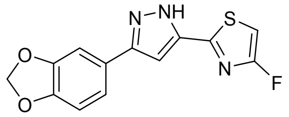	814 ± 2	n.d.
**Designed optimal compounds**	APT-11	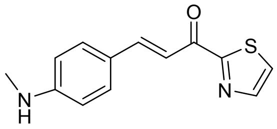	1009 ± 34	376.7 ± 80.7
	APT-12	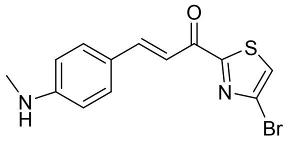	198.1 ± 142.3	586.7/low affinity
	APT-13	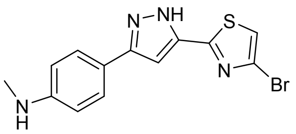	27.8 ± 9.7	92.6 ± 48.8
	APT-14	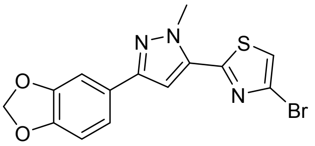	no competition	n.d.

Table S3. Results from LogD determination assay.

C-11 CPM	Description	**Mean value**	**Mean value * dil**
28.68	Blank		
1262.00	OCT2 - 1	1562.0	1562000
1543.00	OCT2 - 2		
1472.00	OCT2 - 3		
1150.00	PBS2 - 1	1089.00	1089.00
1166.00	PBS2 - 2		
964.18	PBS2 - 3		

Calculated logD: $\log D = log_{10}\left(\frac{mean(OCT2.1{:}OCT2.3) * dil_{OCT2}}{mean(PBS2.1{:}PBS2.3) * dil_{PBS2}}\right) = log_{10}\left(\frac{1562.0 * 1000}{1089.00 * 1}\right) = 3.16$

1. ^{1}H and ^{13}C NMR spectra of compounds

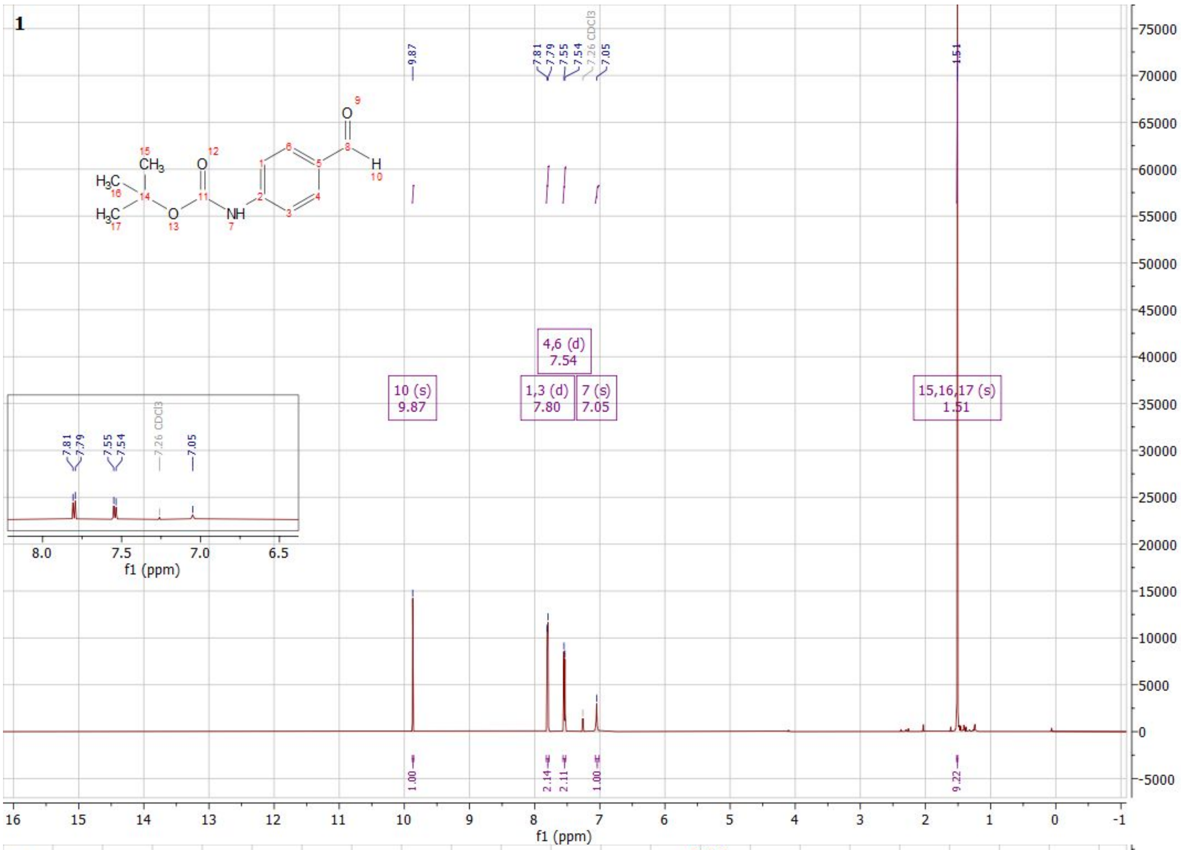

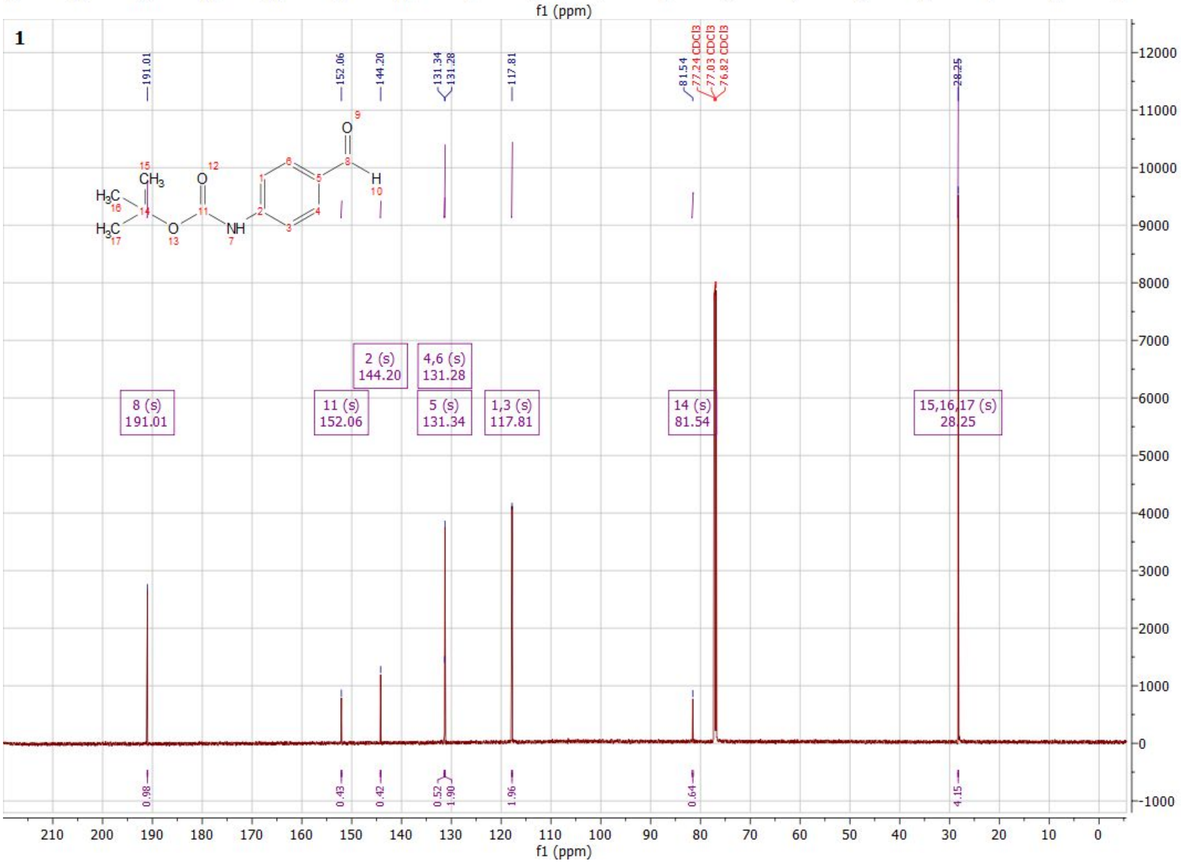

Figure S8. ^{1}H and ^{13}C NMR spectra of **1**.

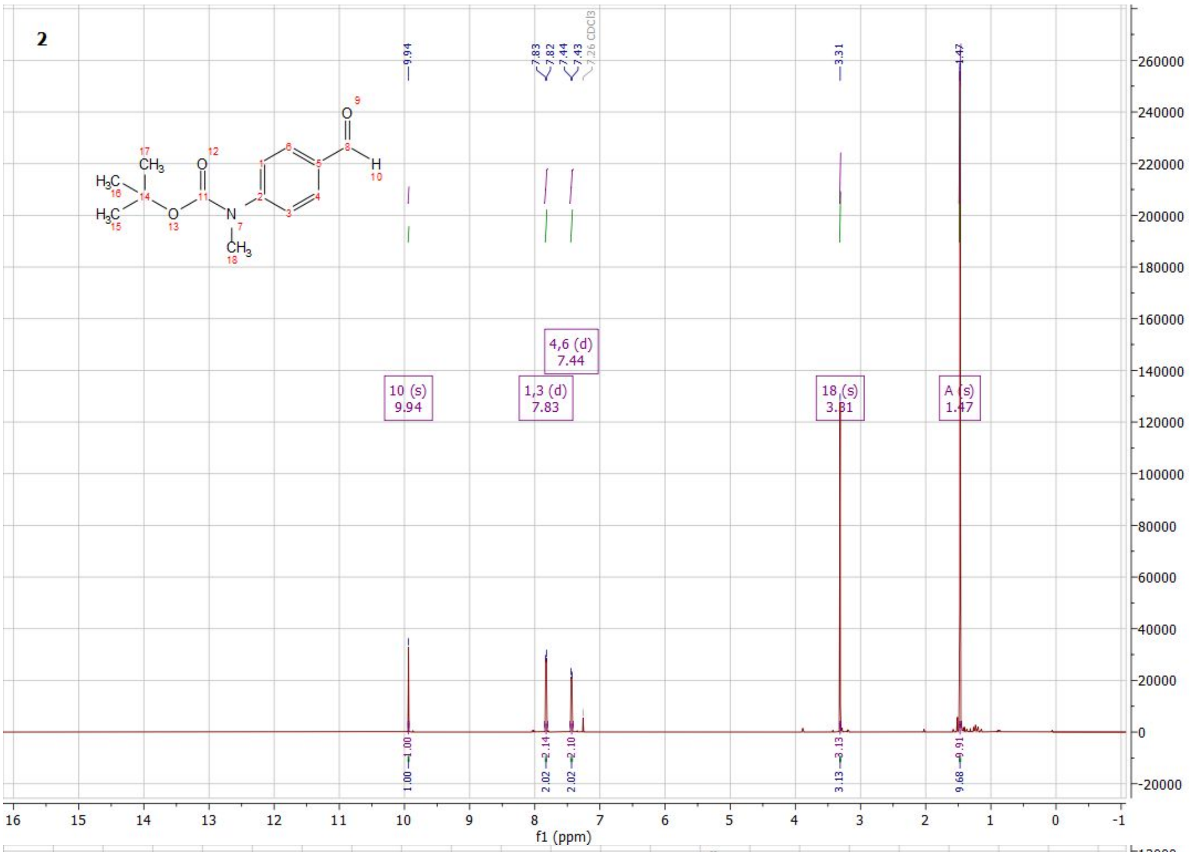

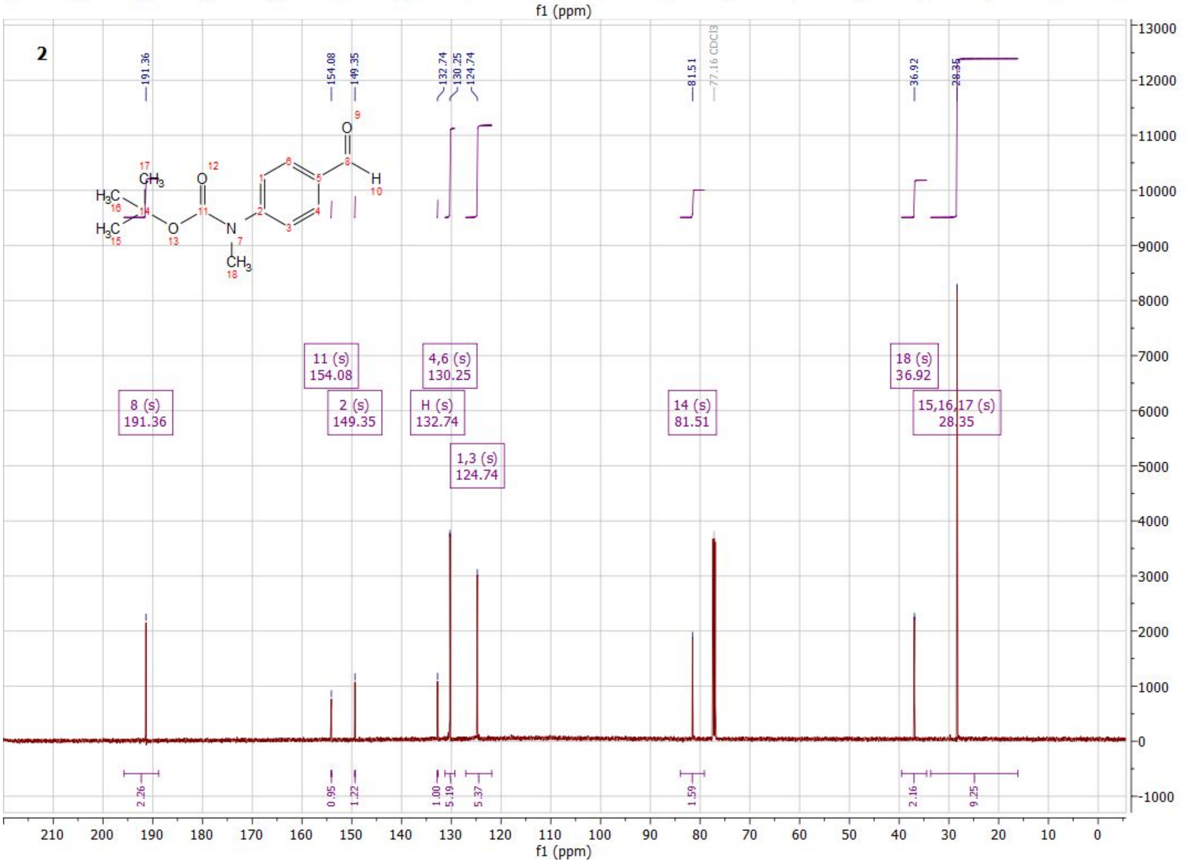

Figure S9. ^{1}H and ^{13}C NMR spectra of **2**.

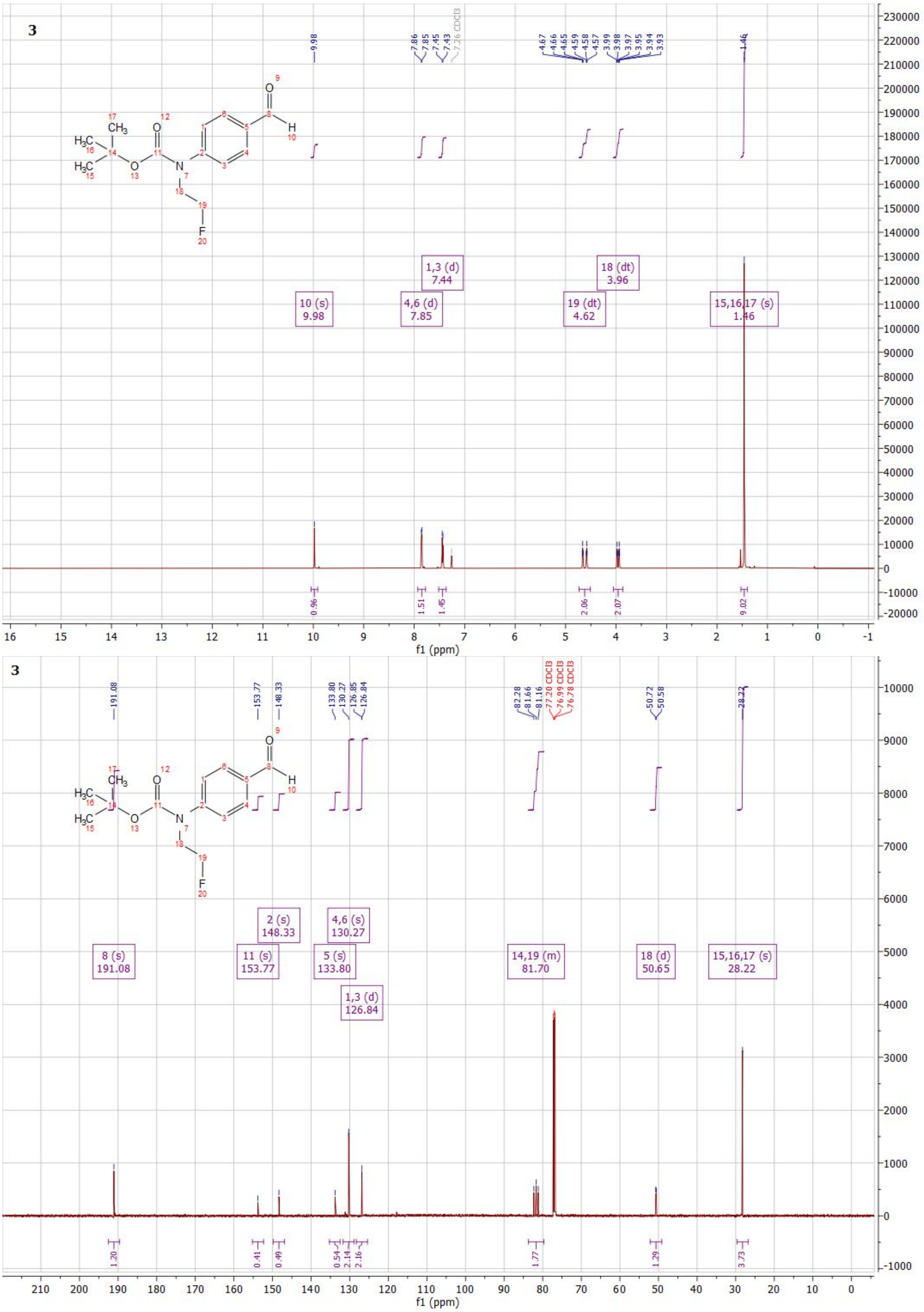

Figure S10. ^{1}H and ^{13}C NMR spectra of **3**.

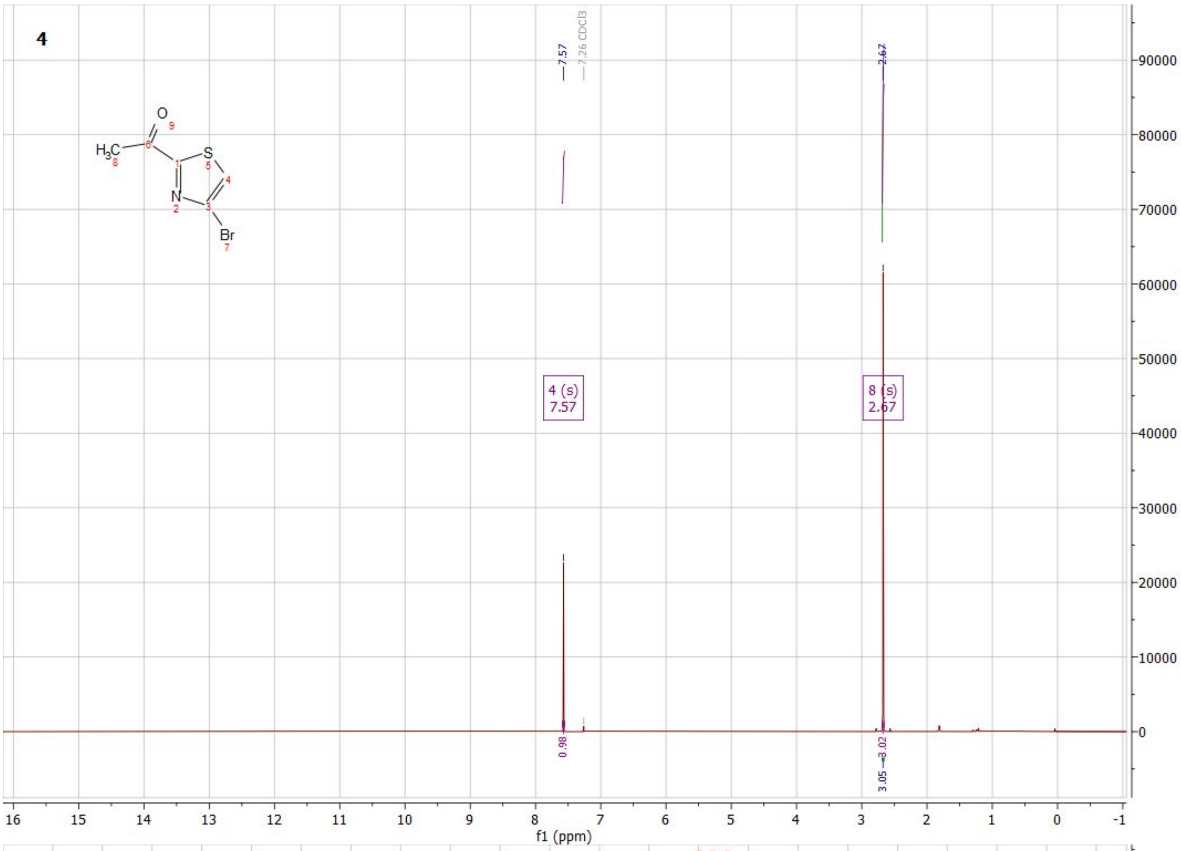

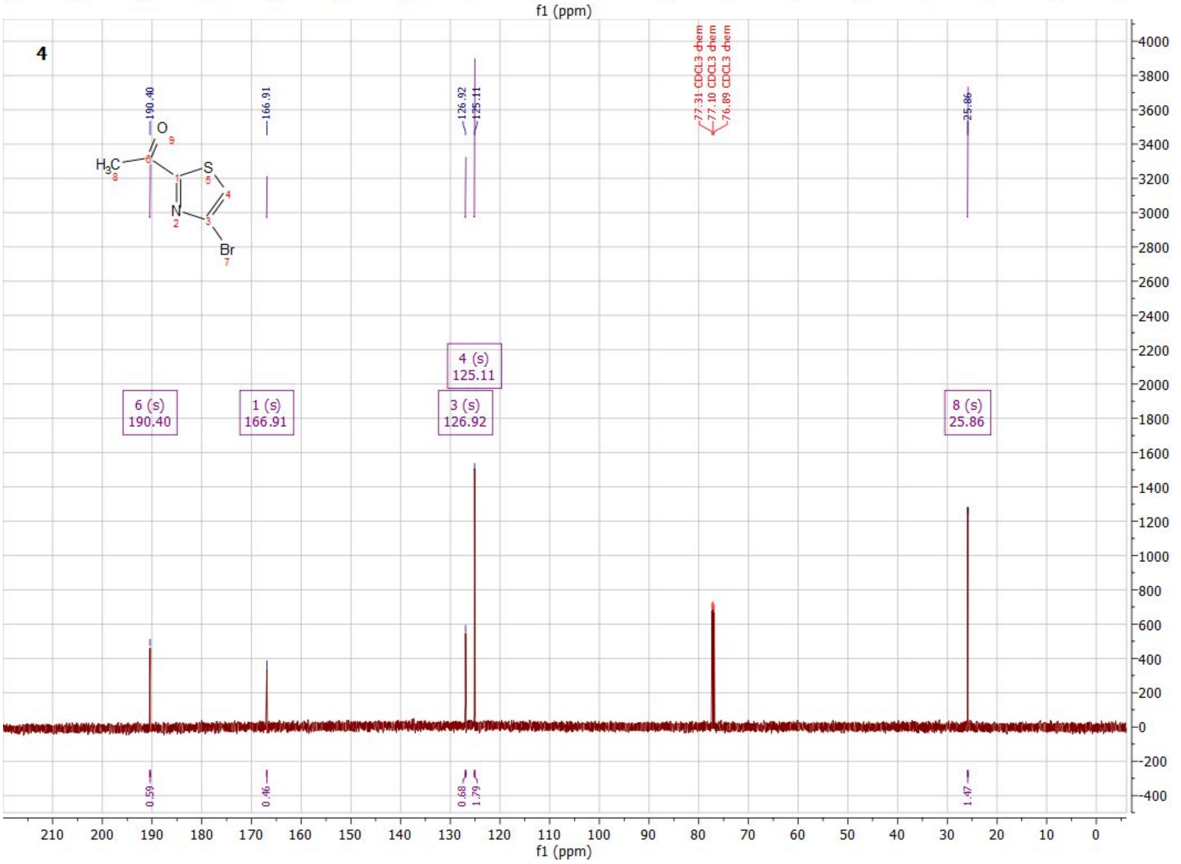

Figure S11. ^{1}H and ^{13}C NMR spectra of **4**.

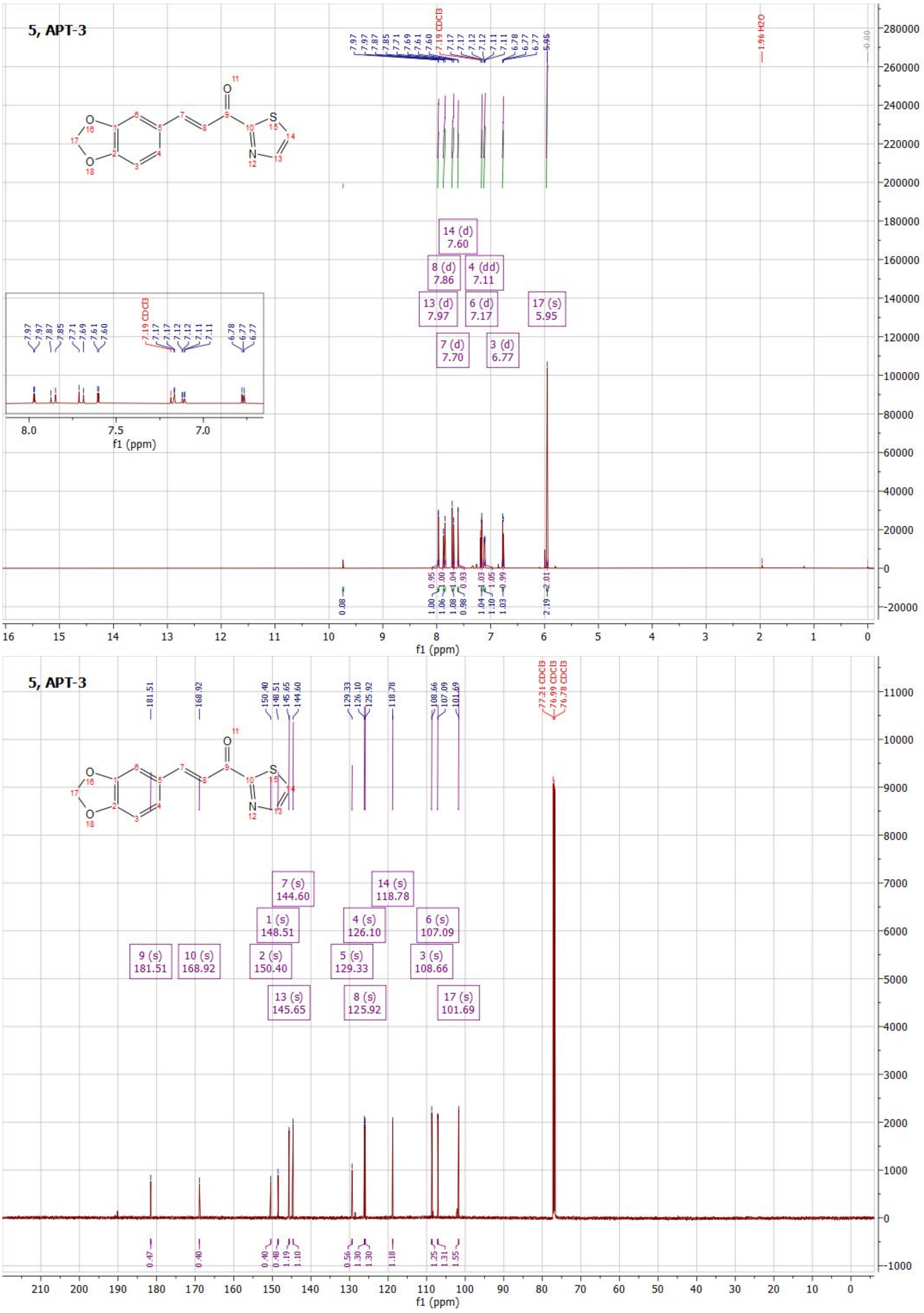

Figure S12. ^{1}H and ^{13}C NMR spectra of **5**.

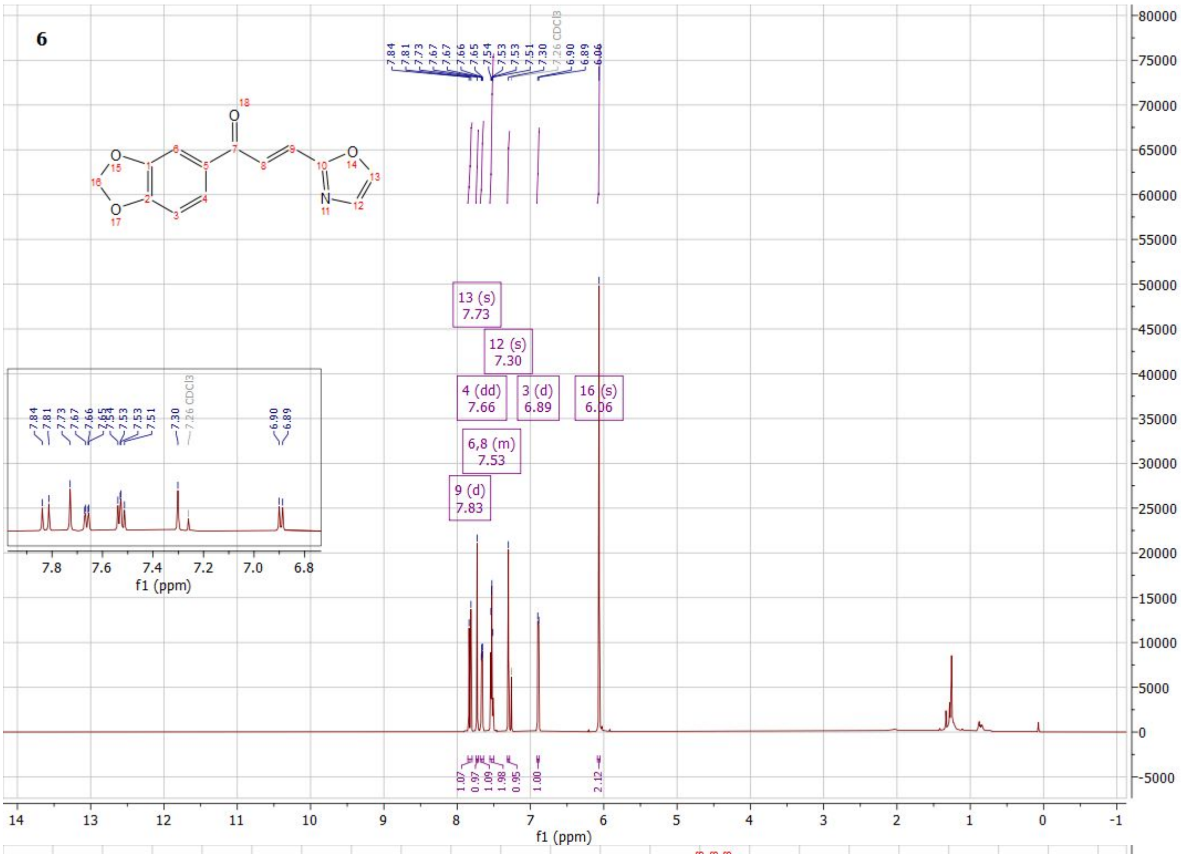

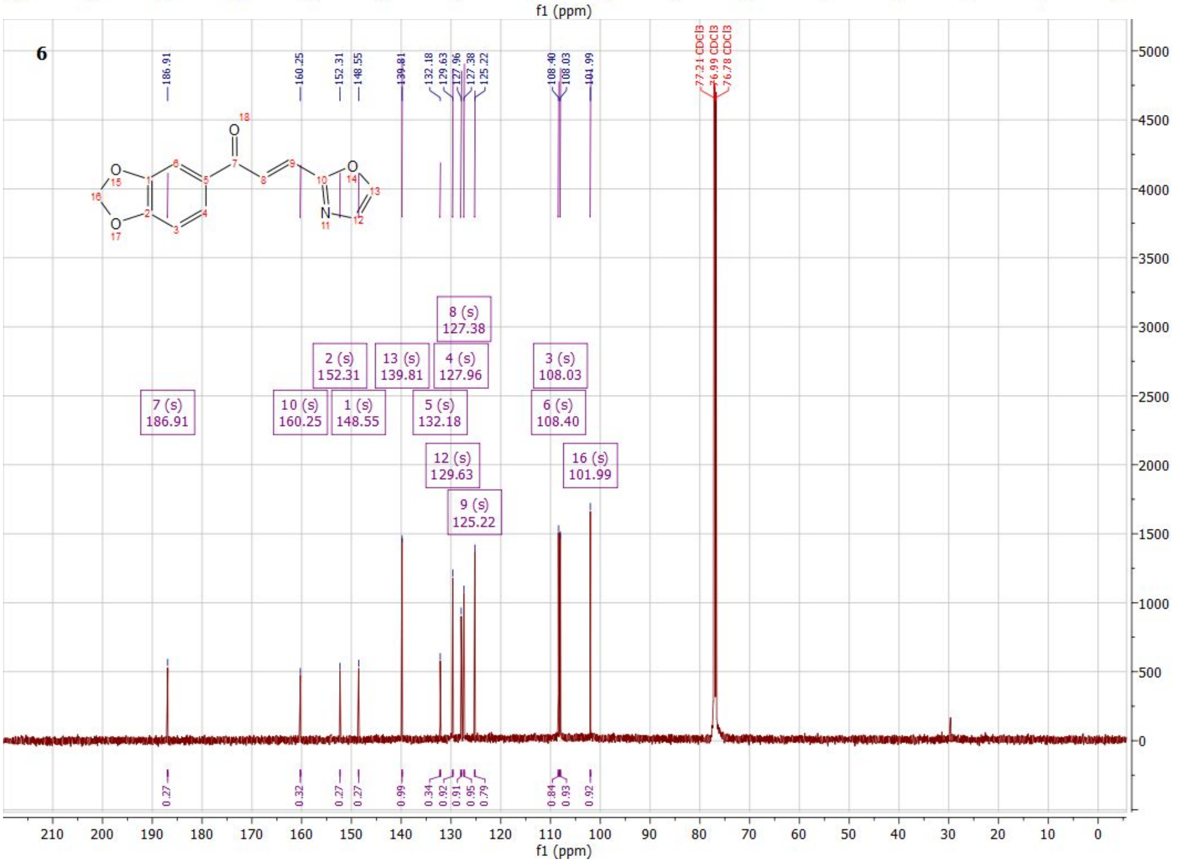

Figure S13. ^{1}H and ^{13}C NMR spectra of **6**.

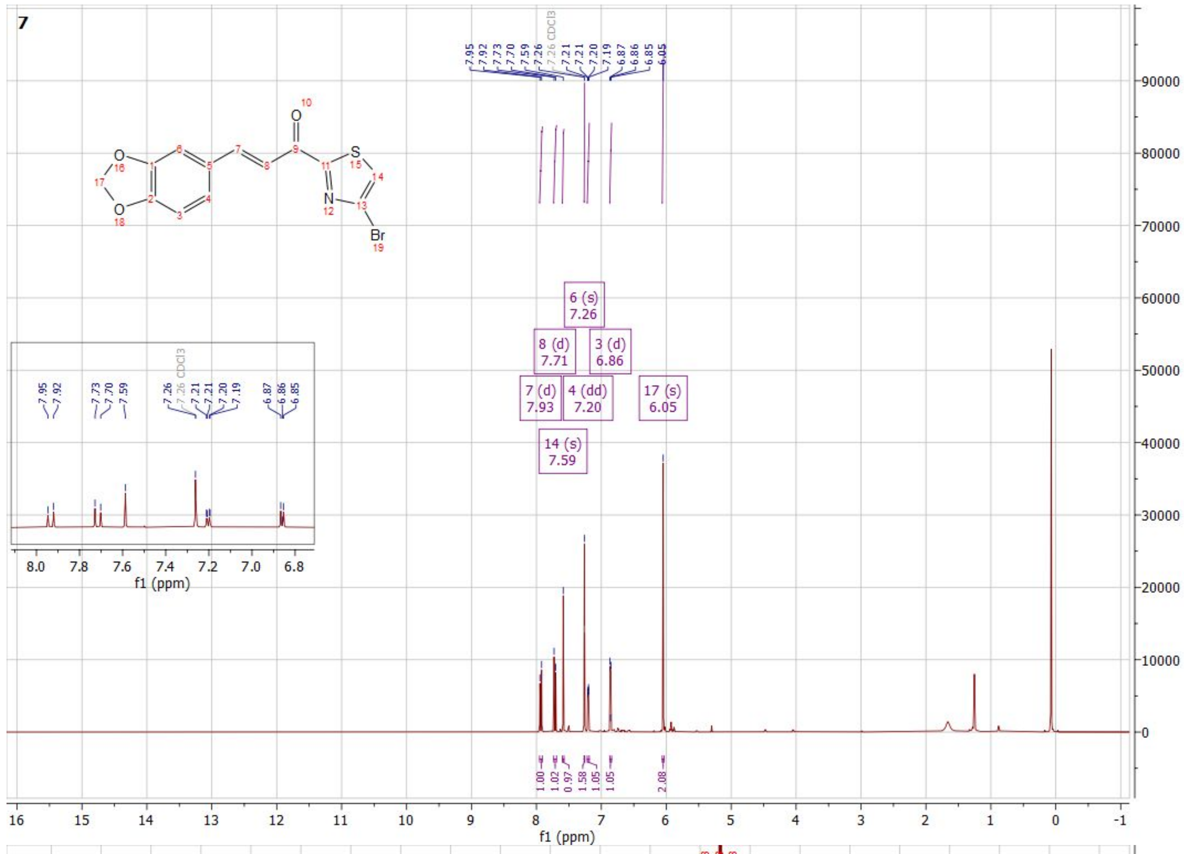

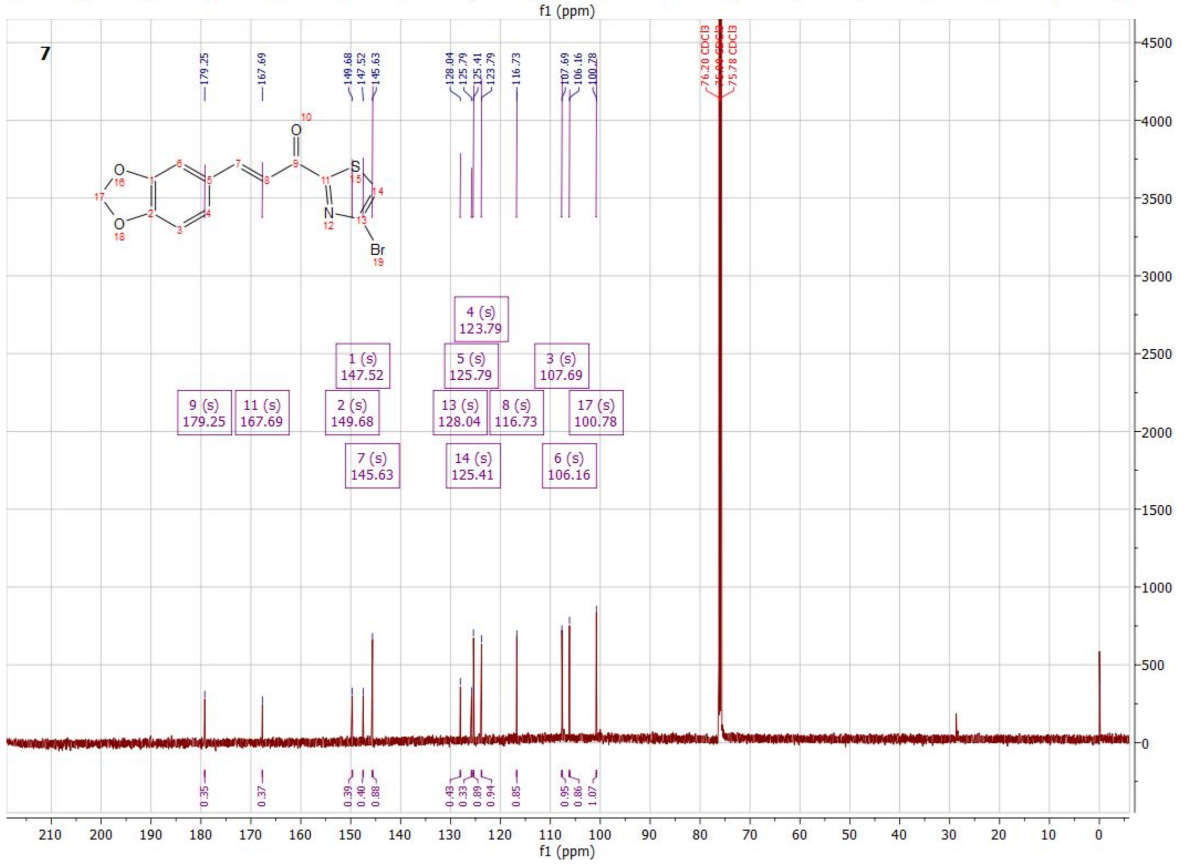

Figure S14. ^{1}H and ^{13}C NMR spectra of **7**.

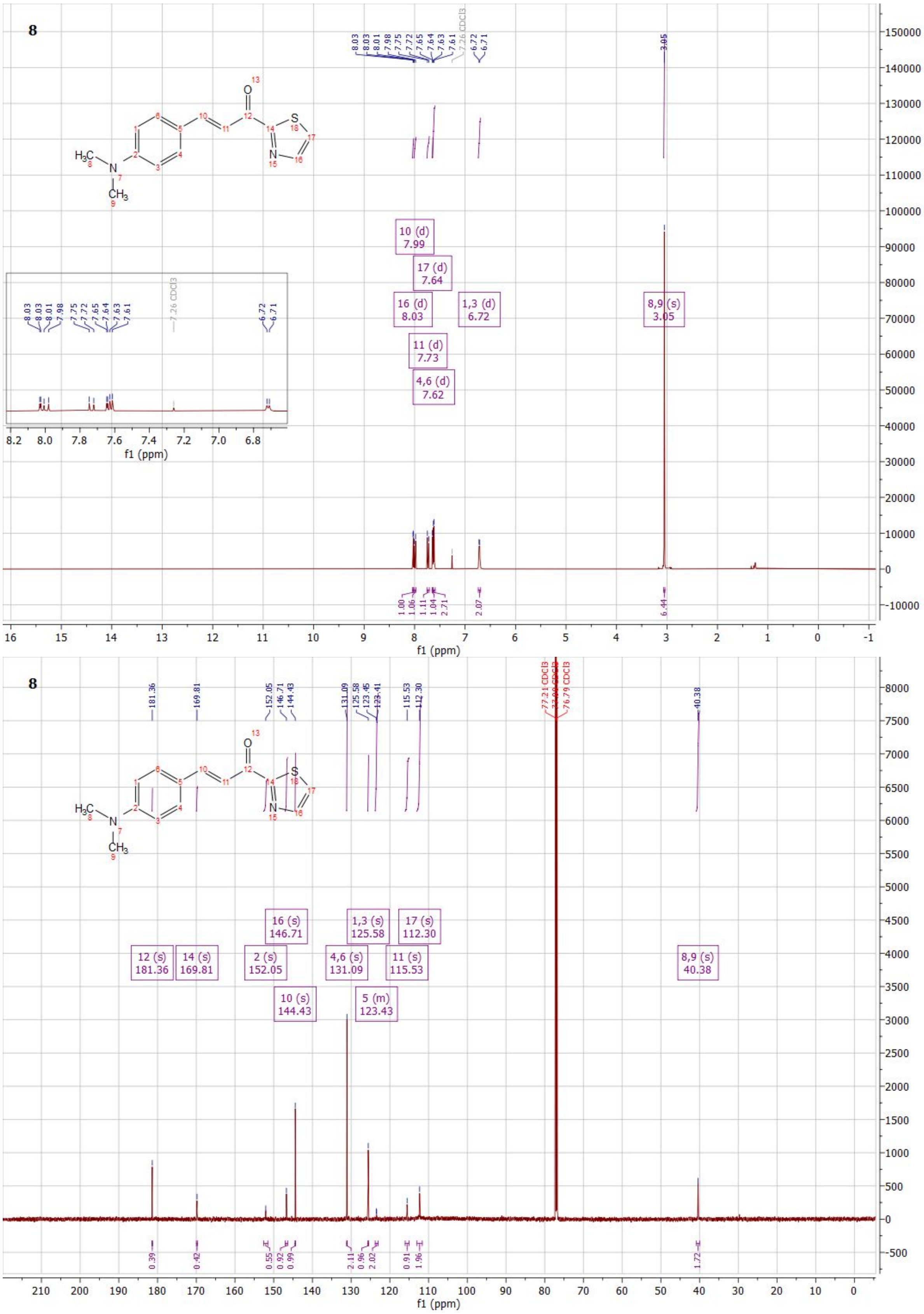

Figure S15. ^{1}H and ^{13}C NMR spectra of **8**.

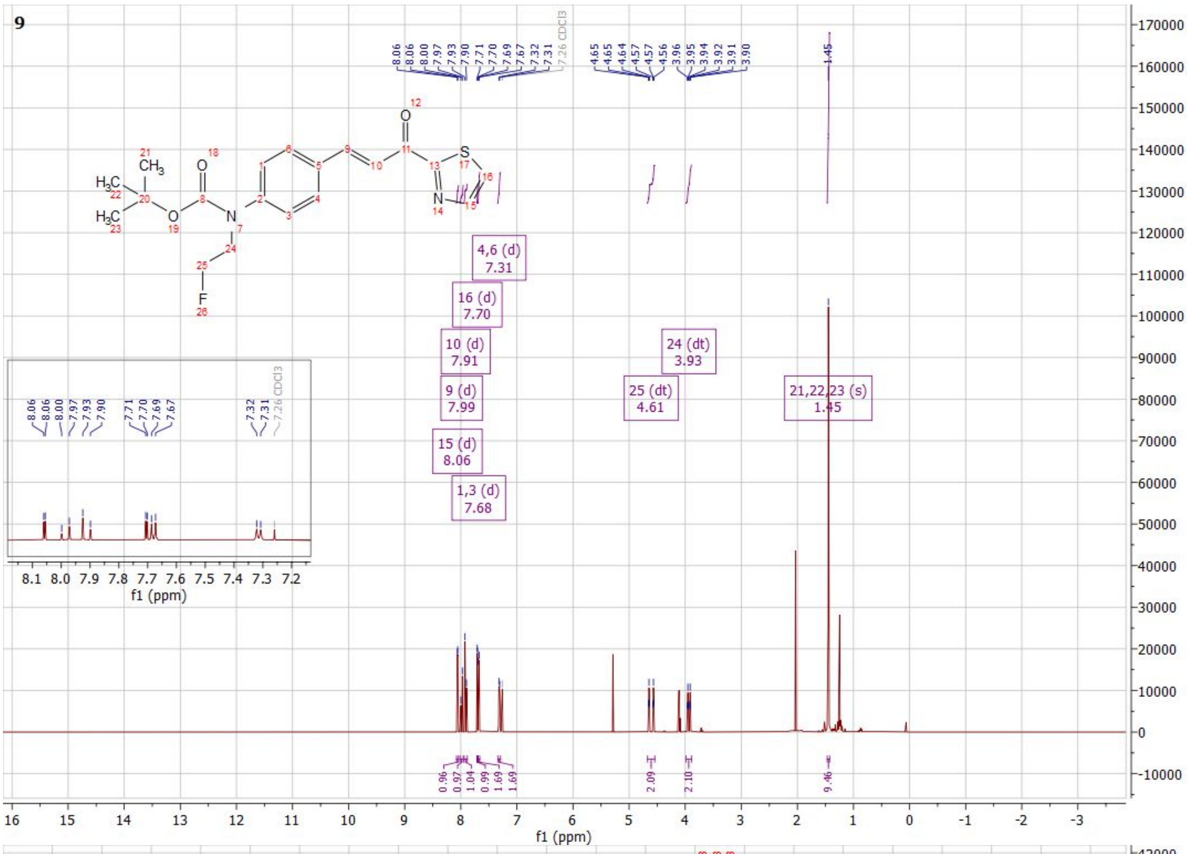

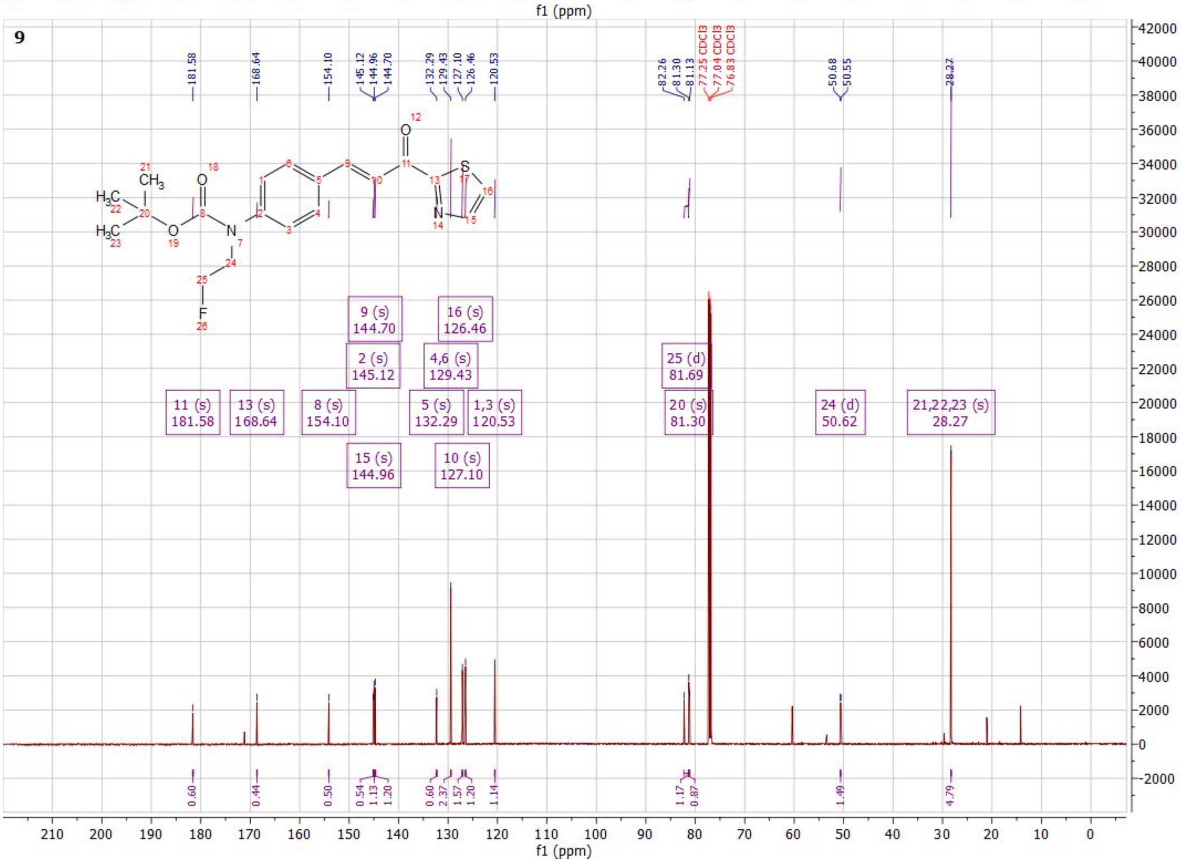

Figure S16. ^{1}H and ^{13}C NMR spectra of **9**.

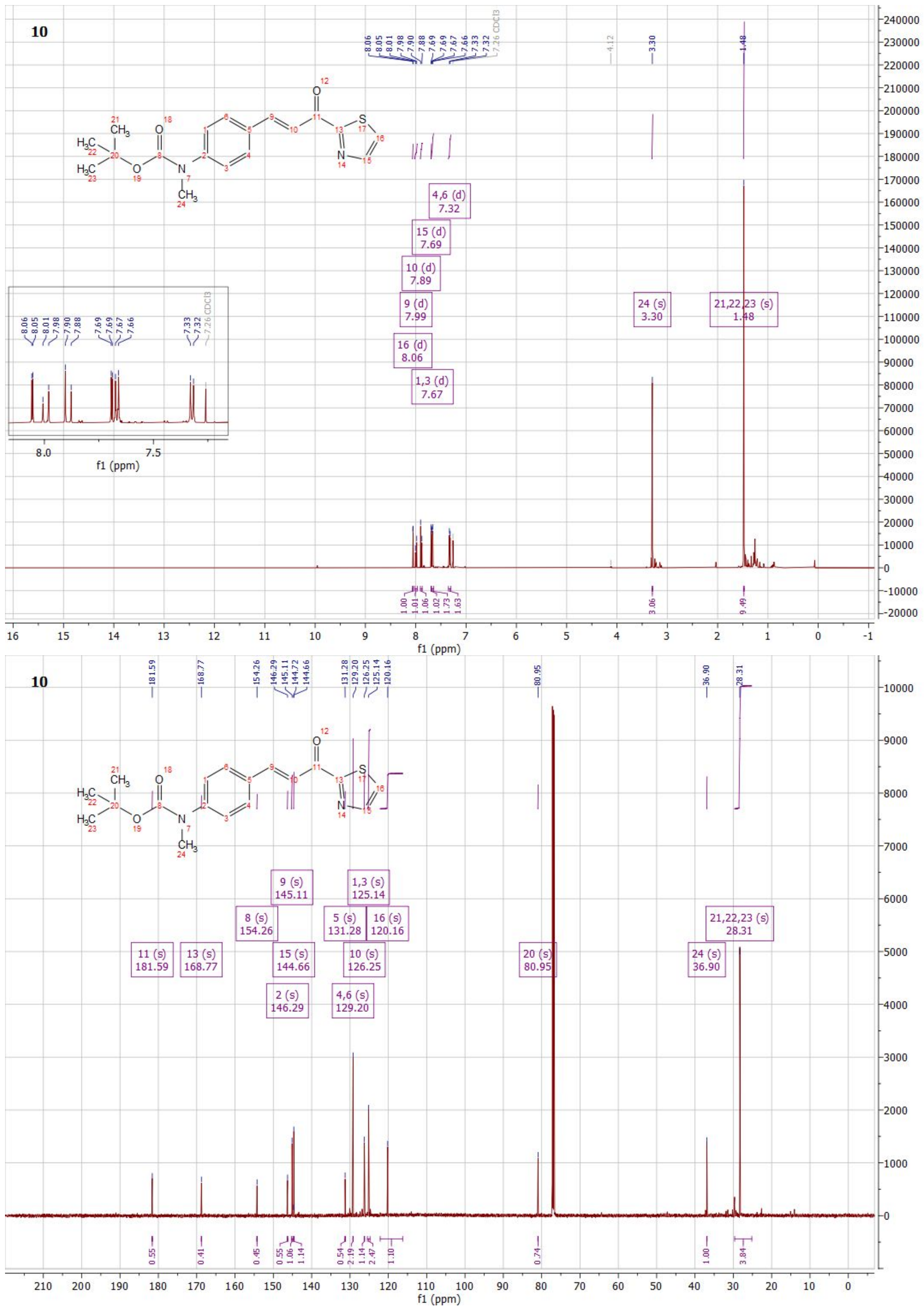

Figure S17. ^{1}H and ^{13}C NMR spectra of **10**.

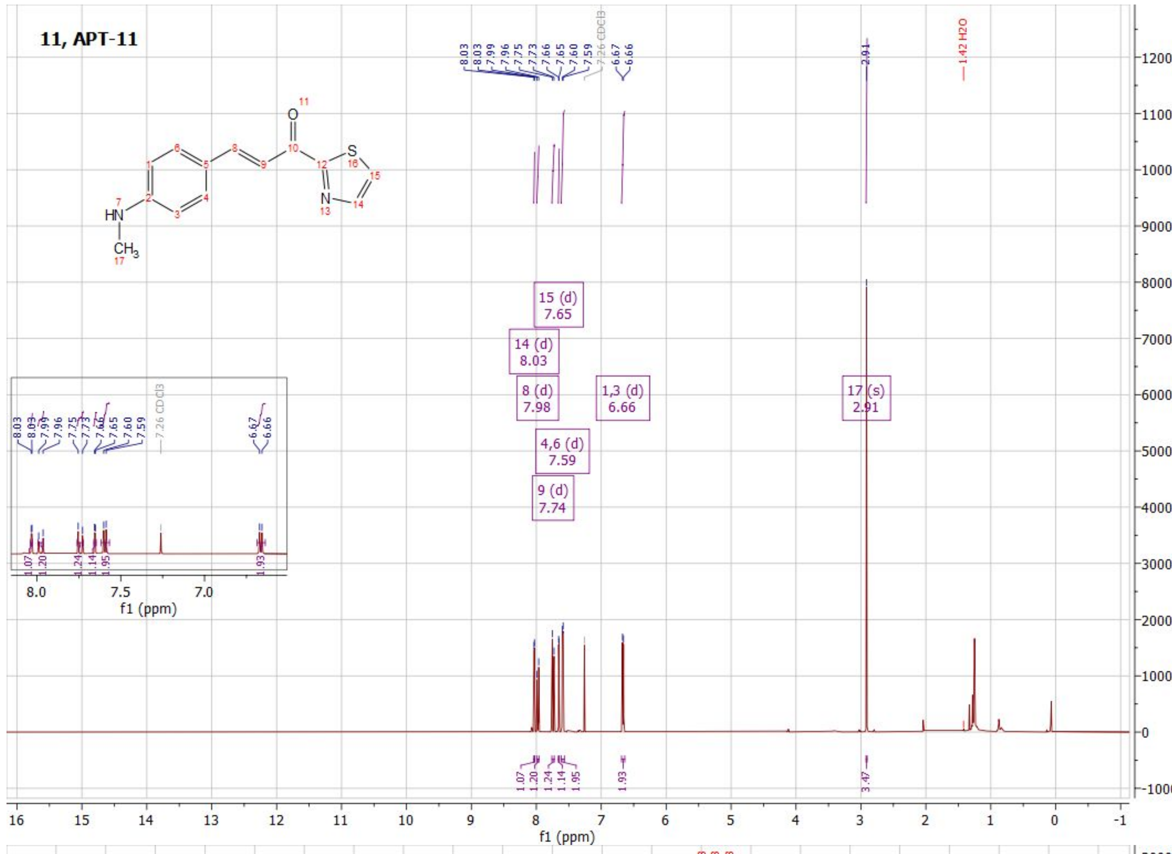

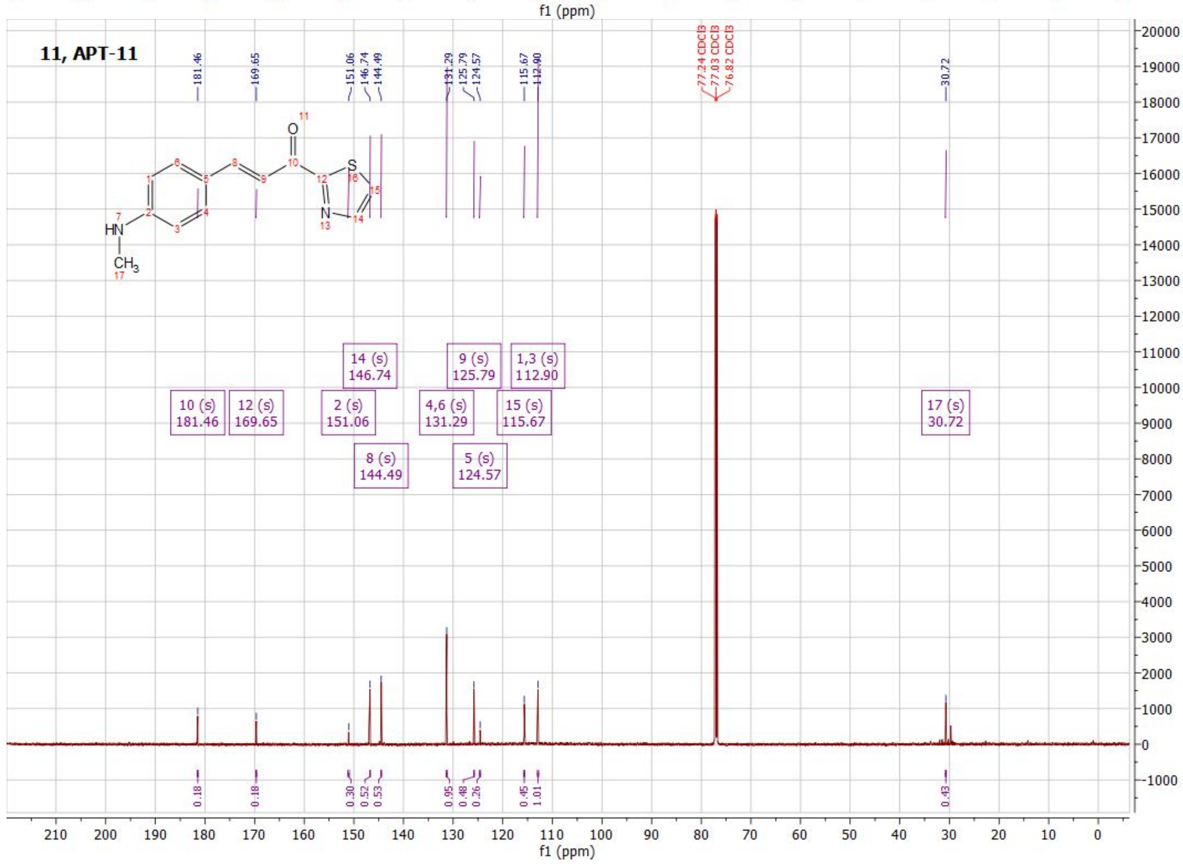

Figure S18. ^{1}H and ^{13}C NMR spectra of **11**.

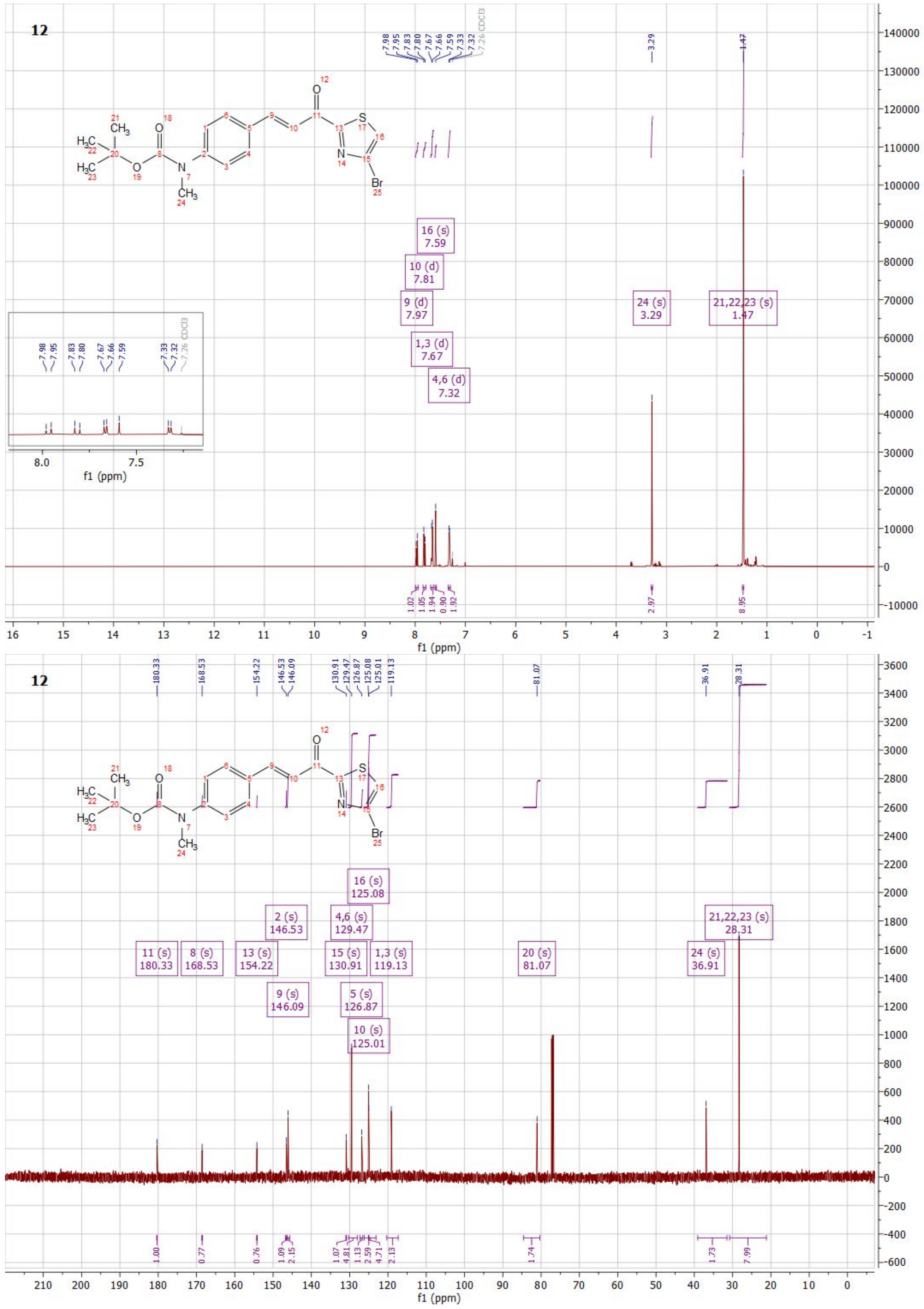

Figure S19. ^{1}H and ^{13}C NMR spectra of **12**.

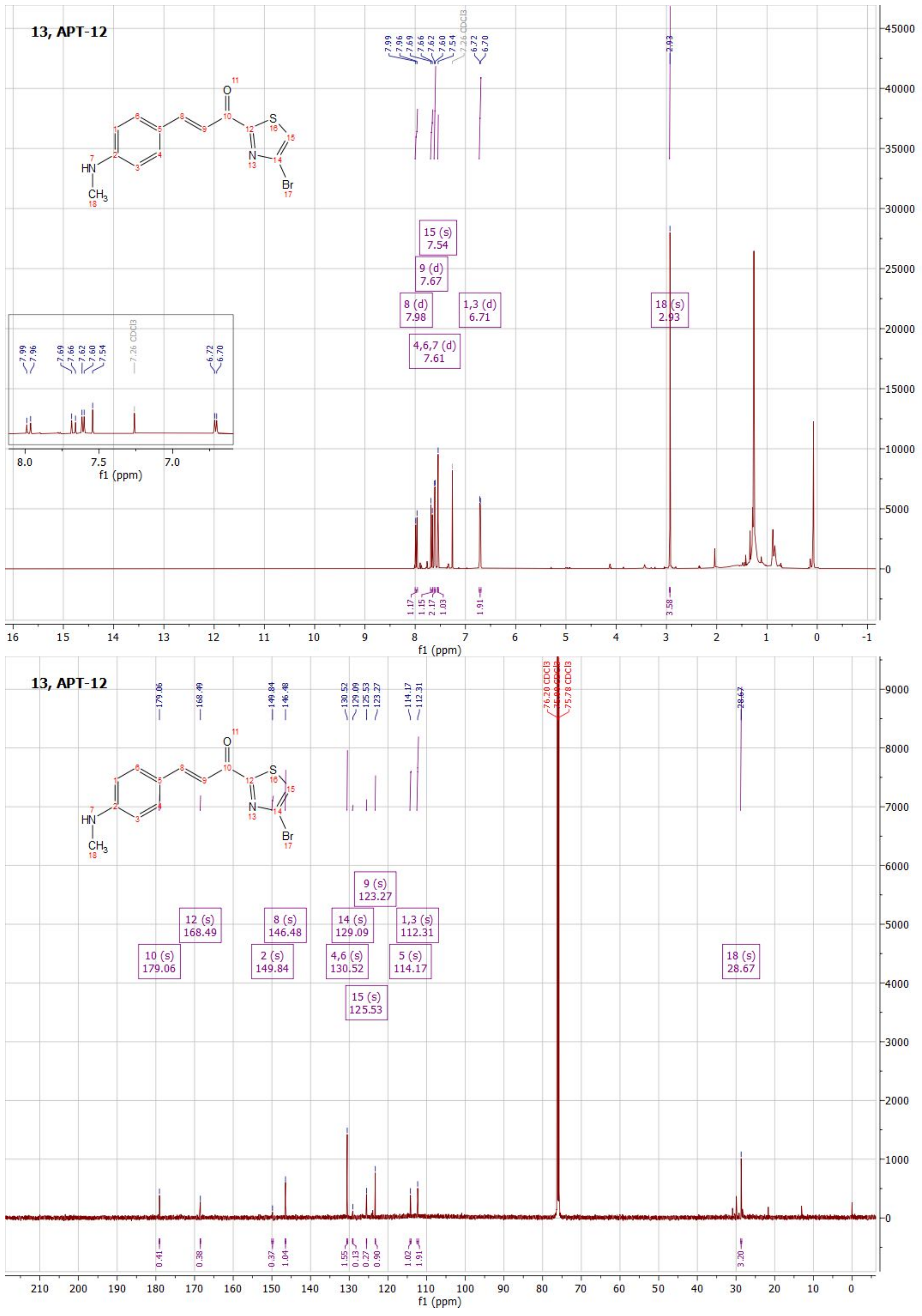

Figure S20. ^{1}H and ^{13}C NMR spectra of **13**.

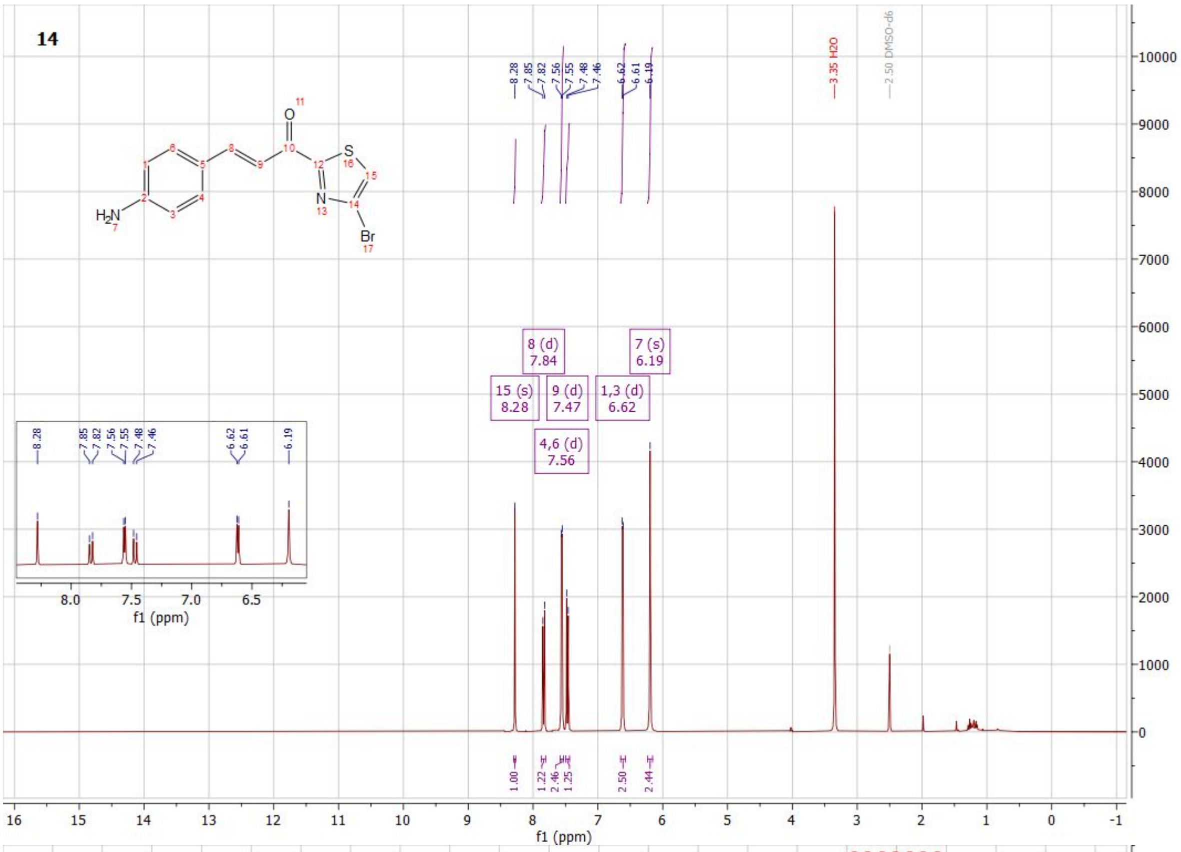

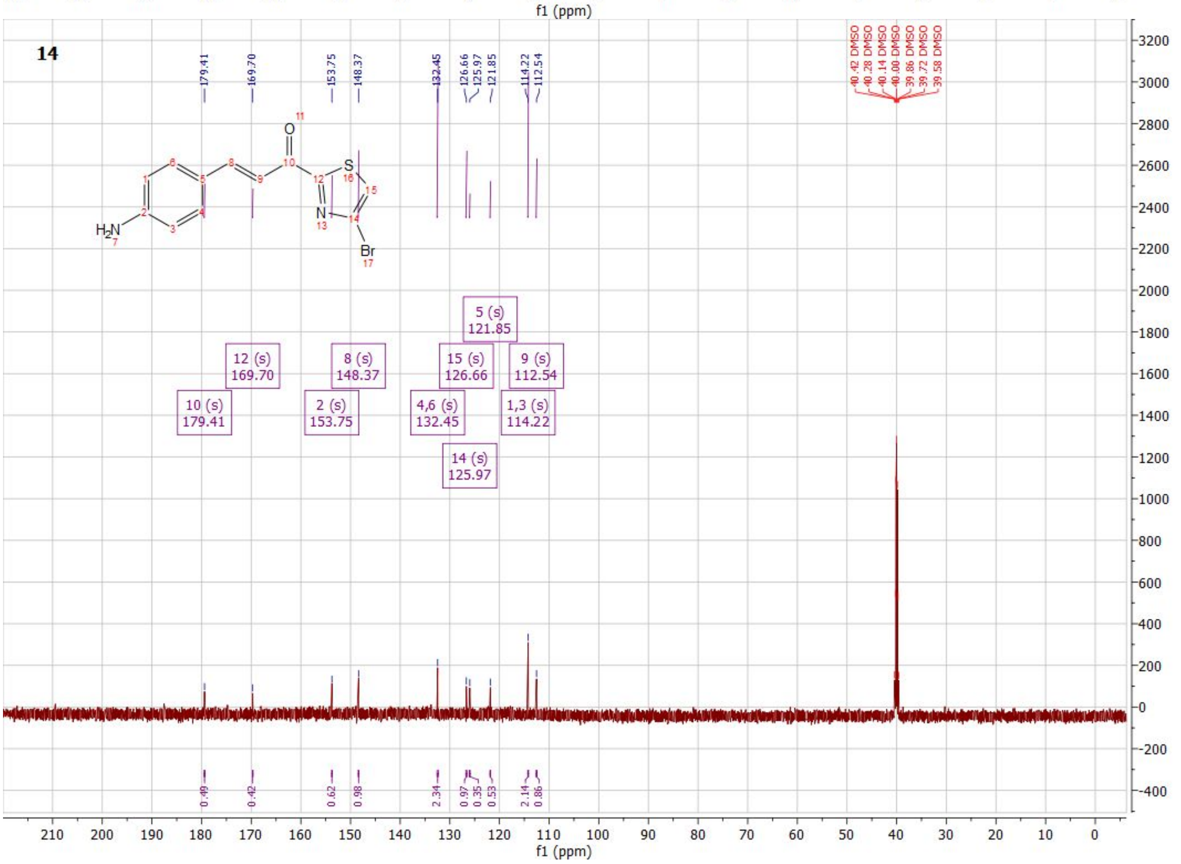

Figure S21. ^{1}H and ^{13}C NMR spectra of **14**.

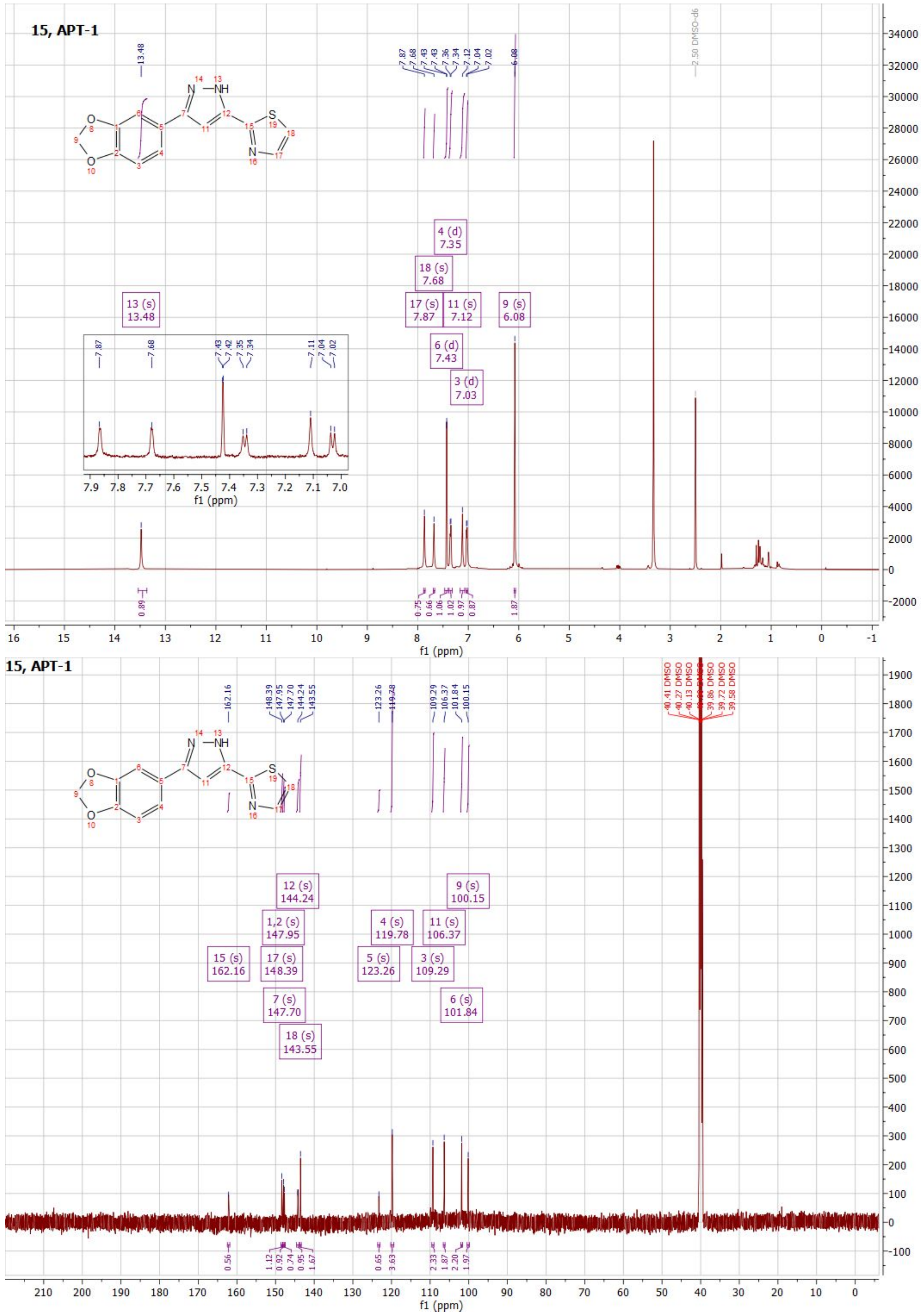

Figure S22. ^{1}H and ^{13}C NMR spectra of **15**.

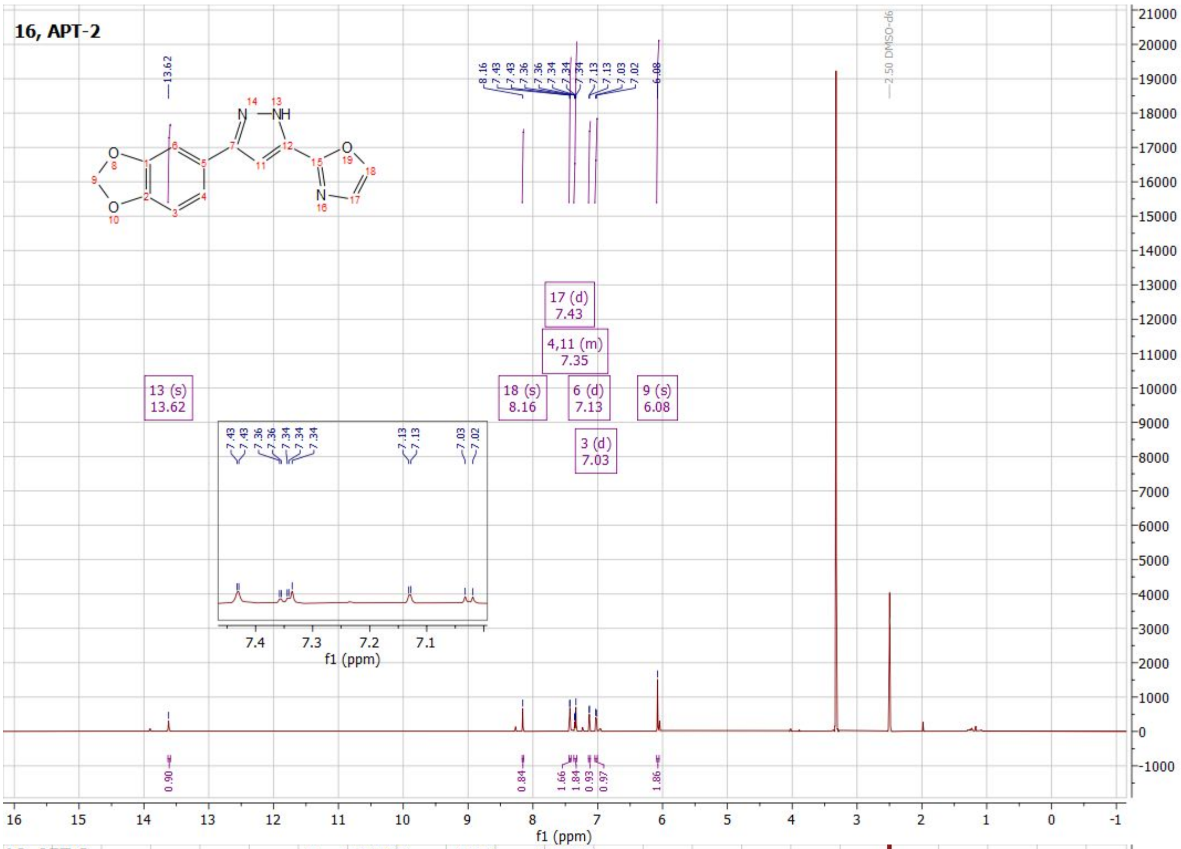

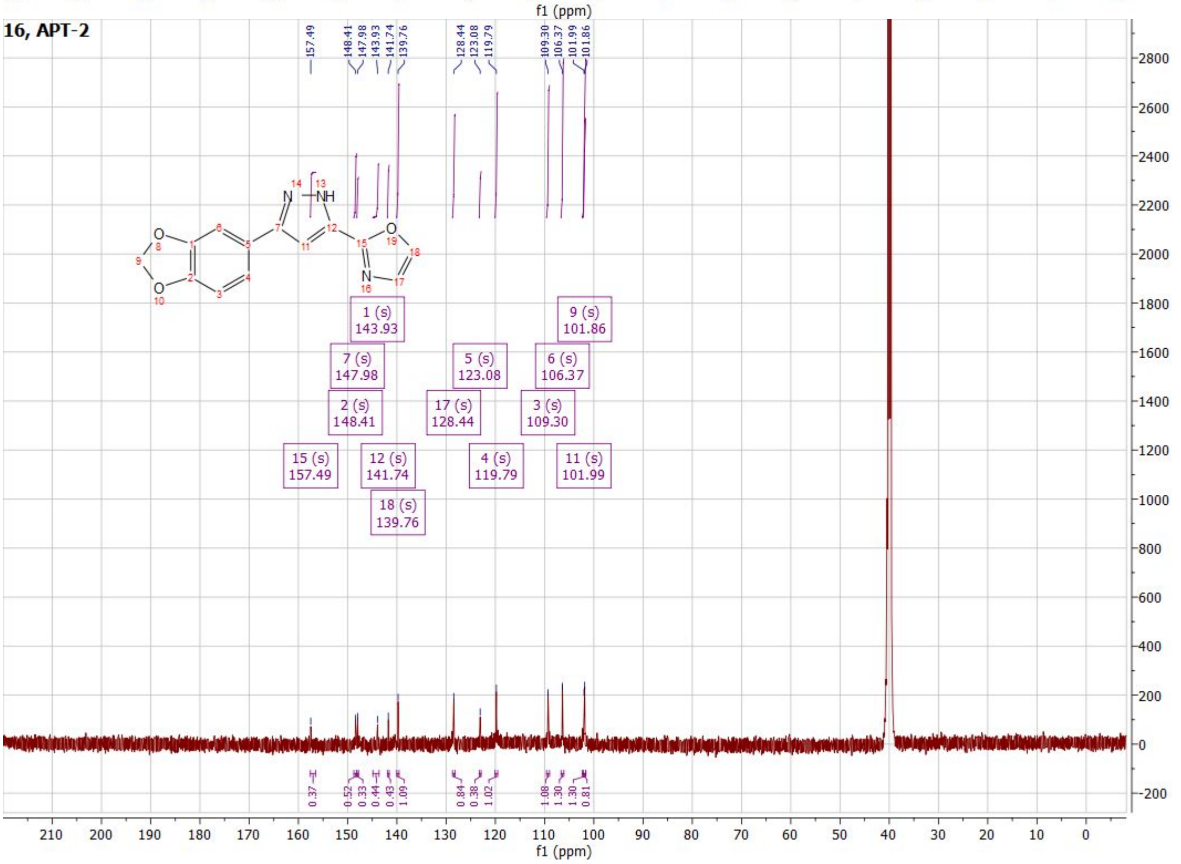

Figure S23. ^{1}H and ^{13}C NMR spectra of **16**.

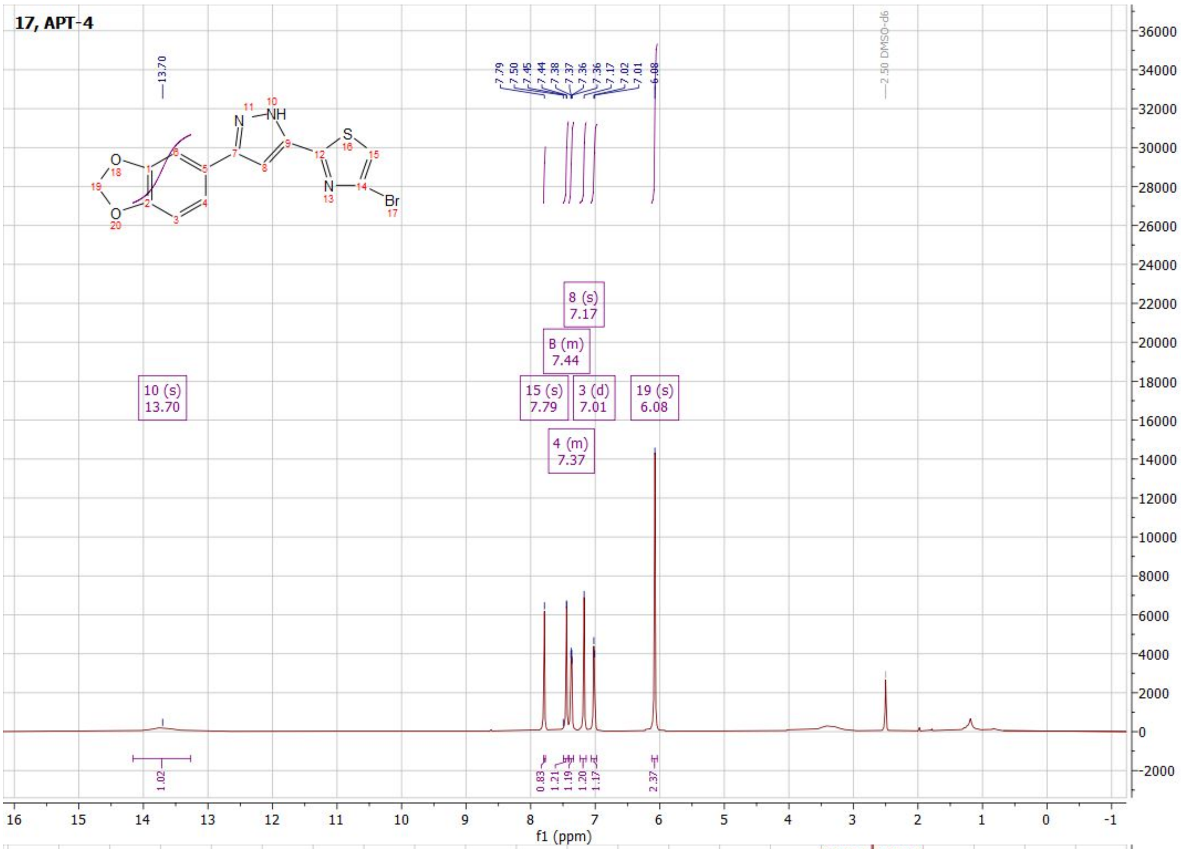

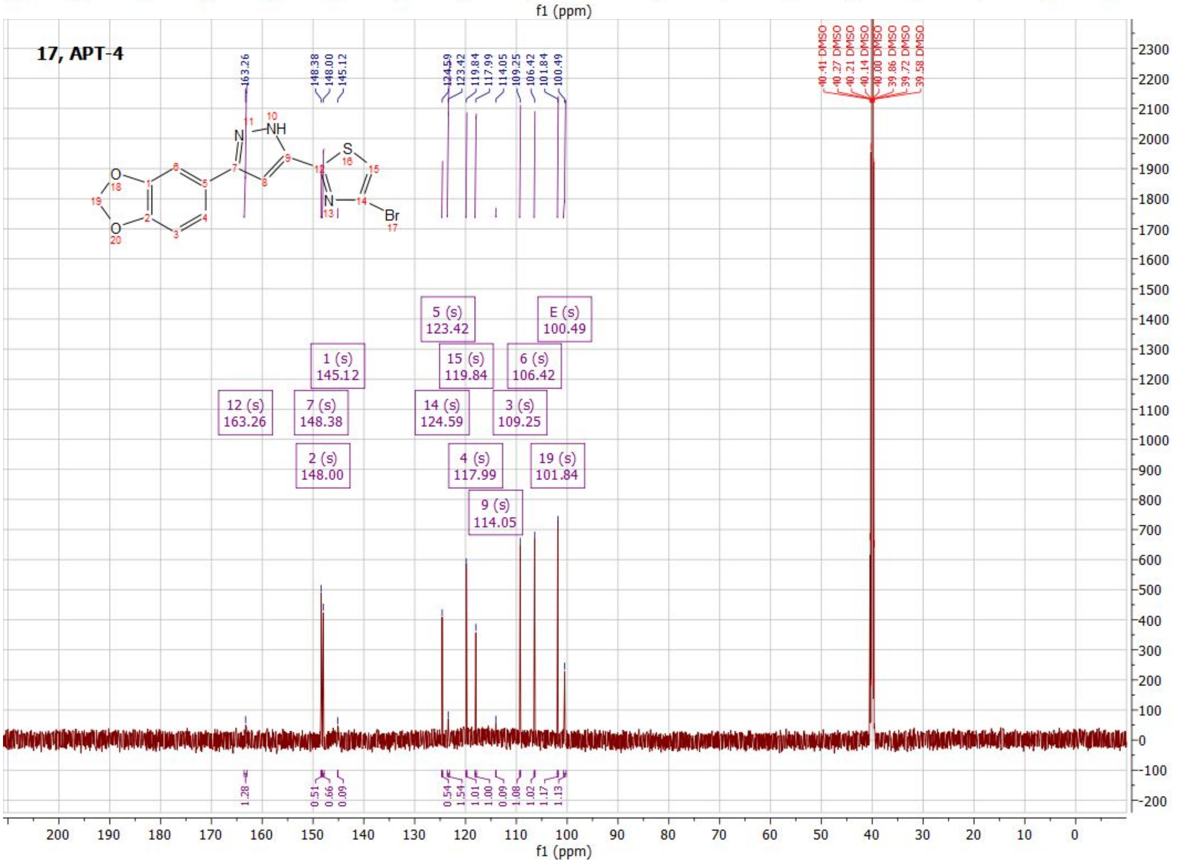

Figure S24. ^{1}H and ^{13}C NMR spectra of **17**.

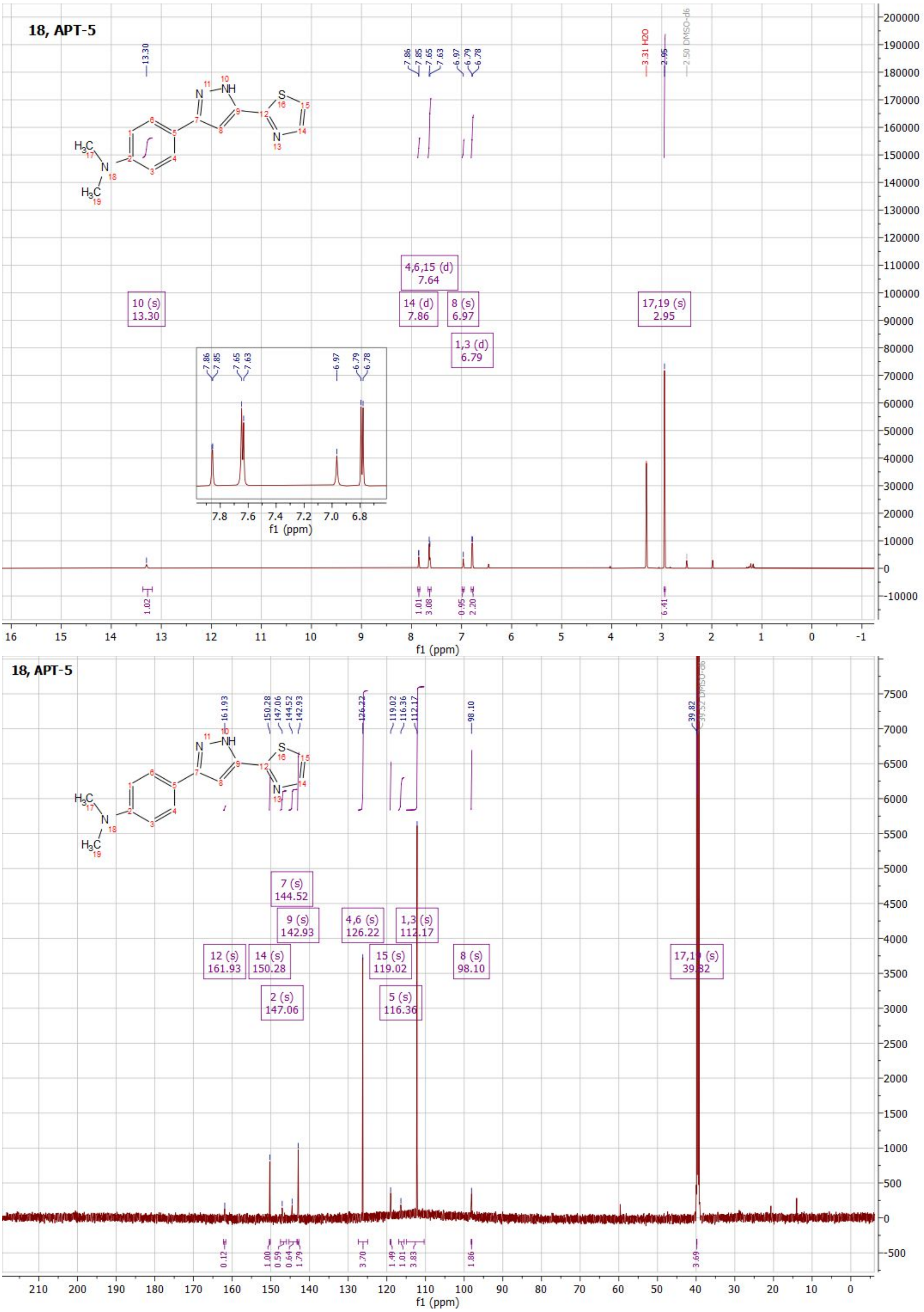

Figure S25. ^{1}H and ^{13}C NMR spectra of **18**.

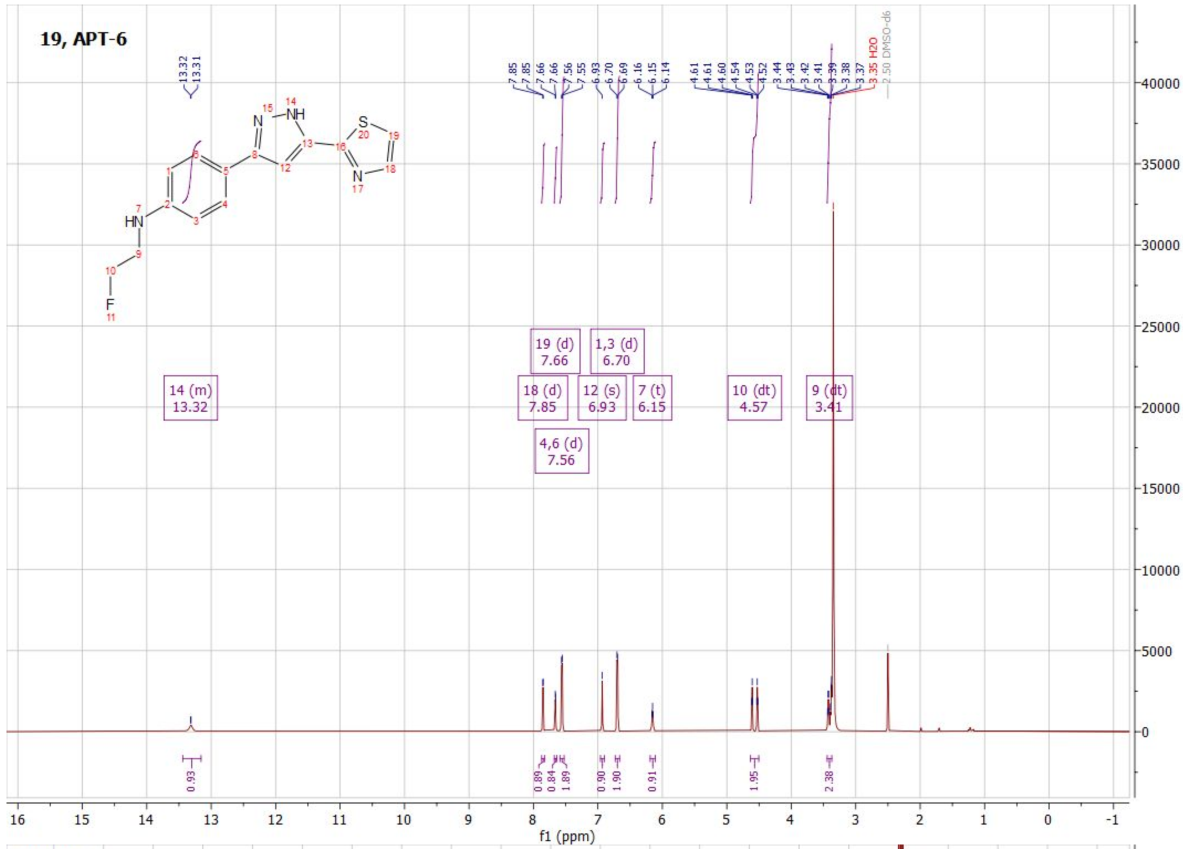

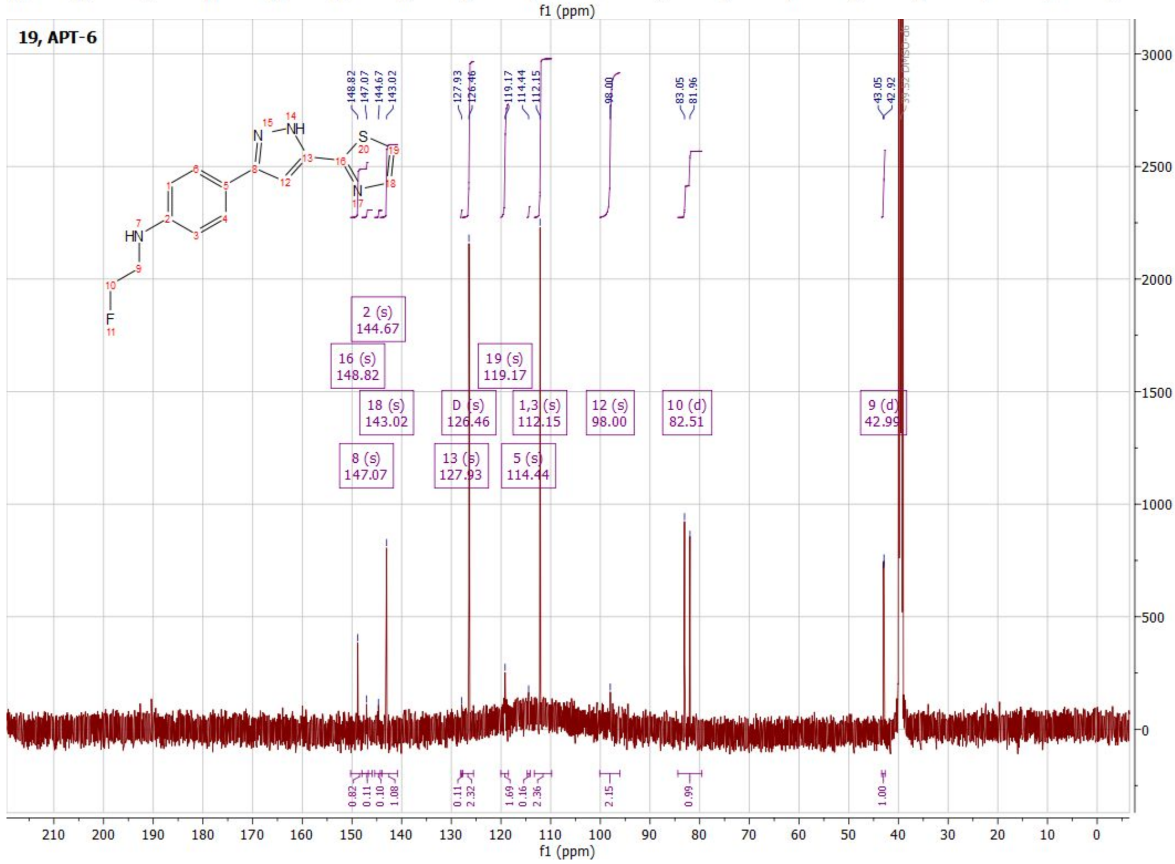

Figure S26. ^{1}H and ^{13}C NMR spectra of **19**.

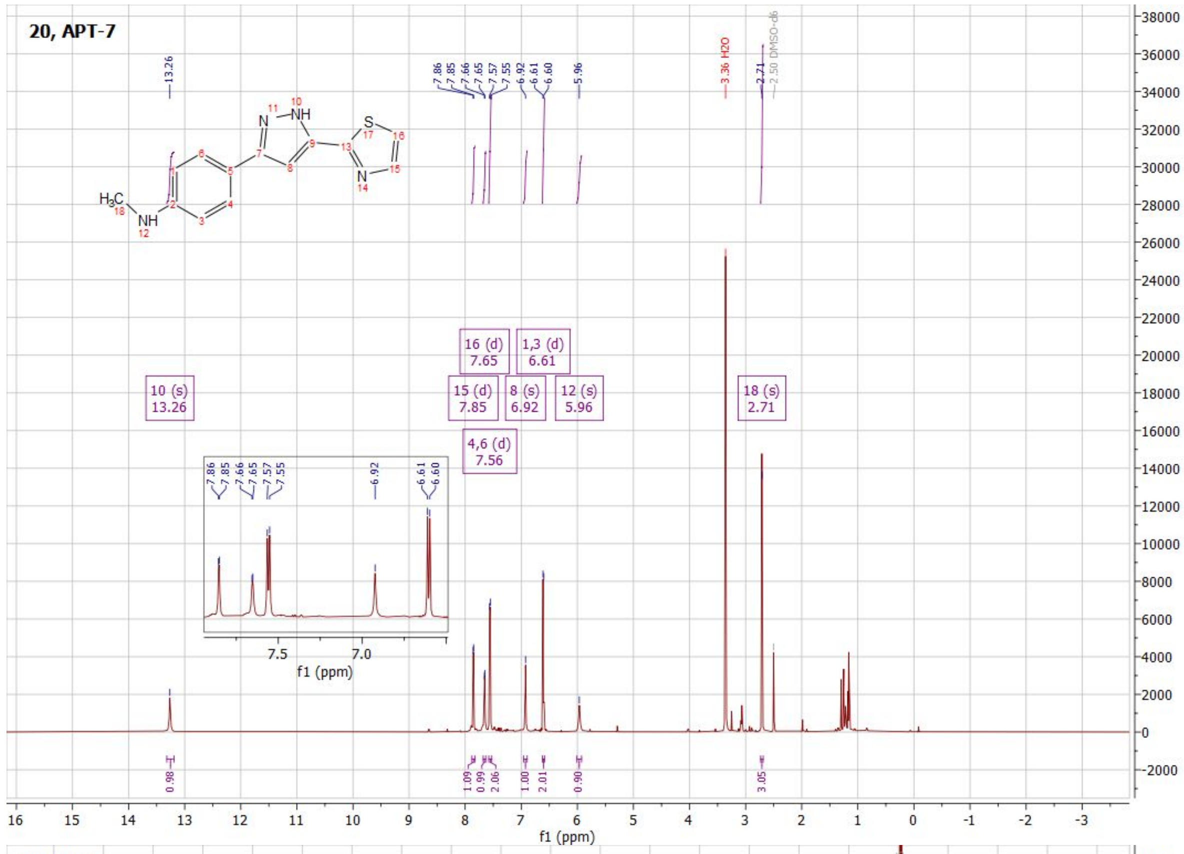

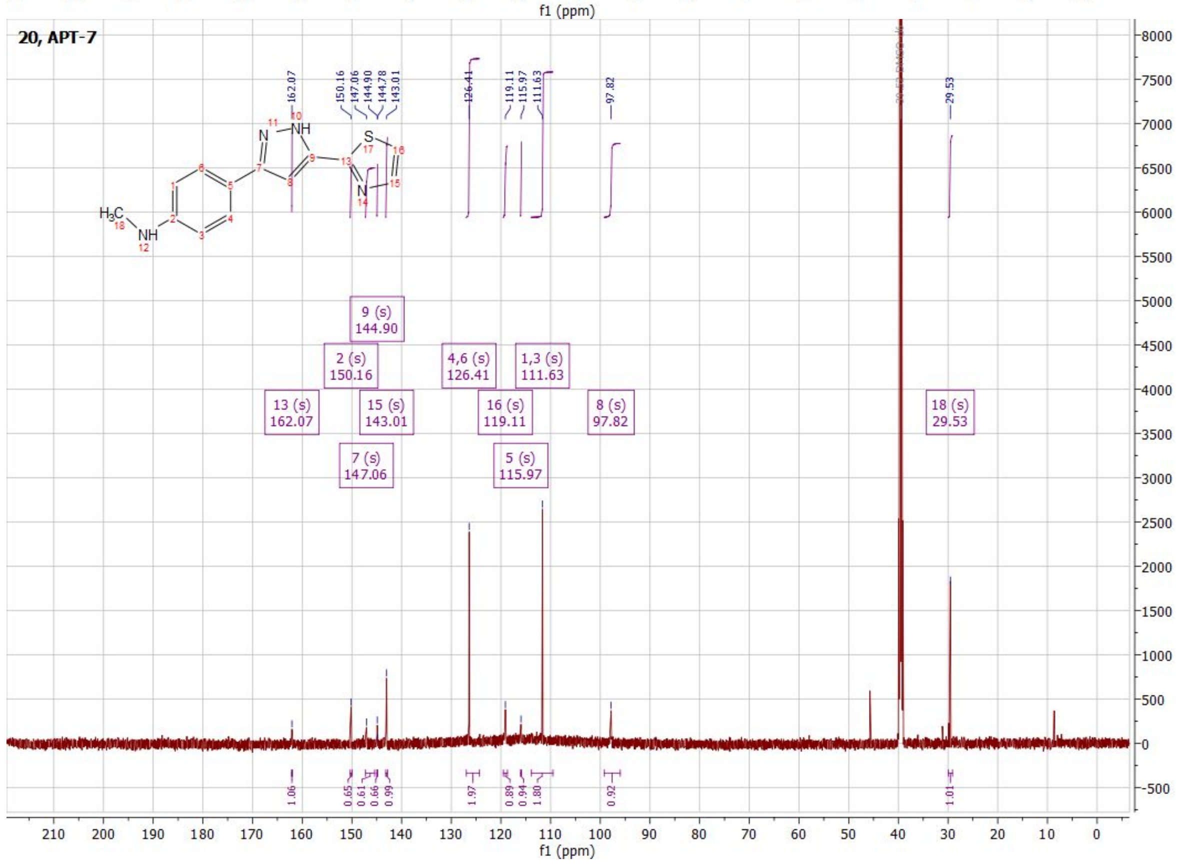

Figure S27. ^{1}H and ^{13}C NMR spectra of **20**.

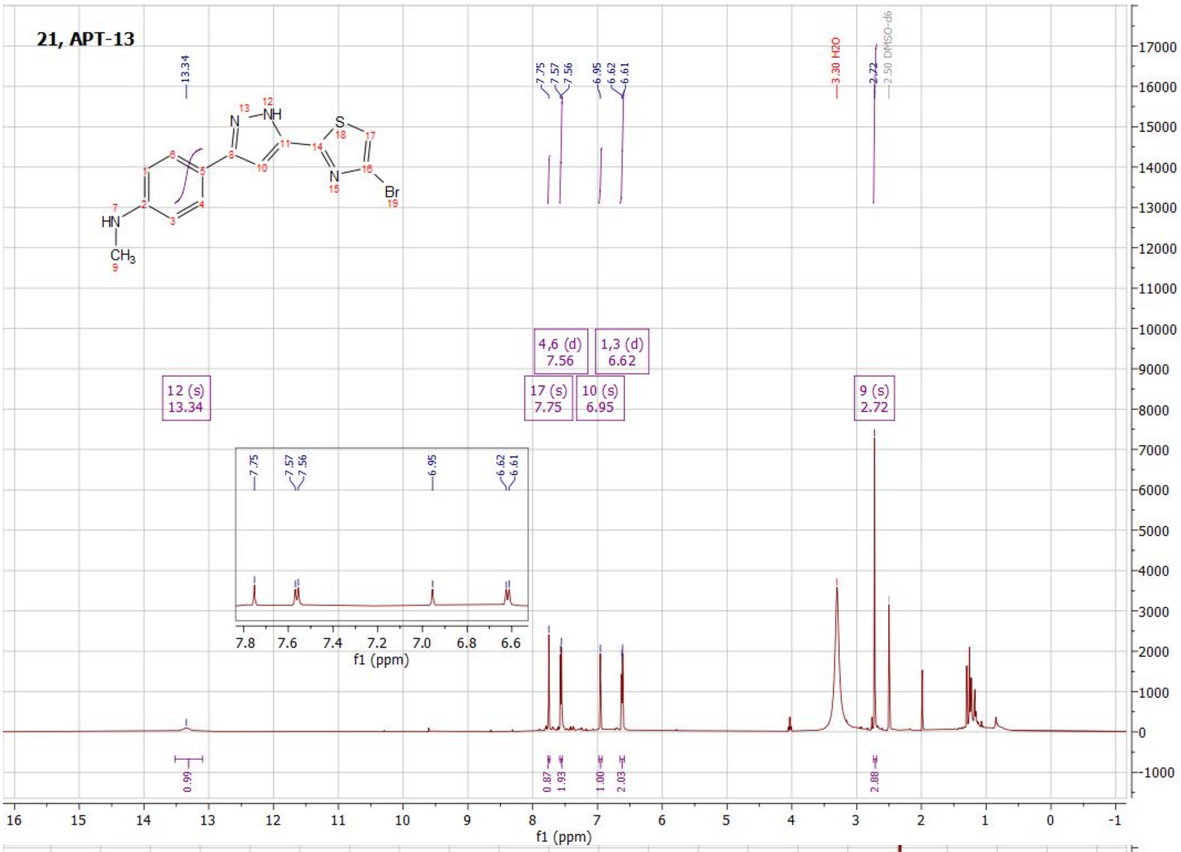

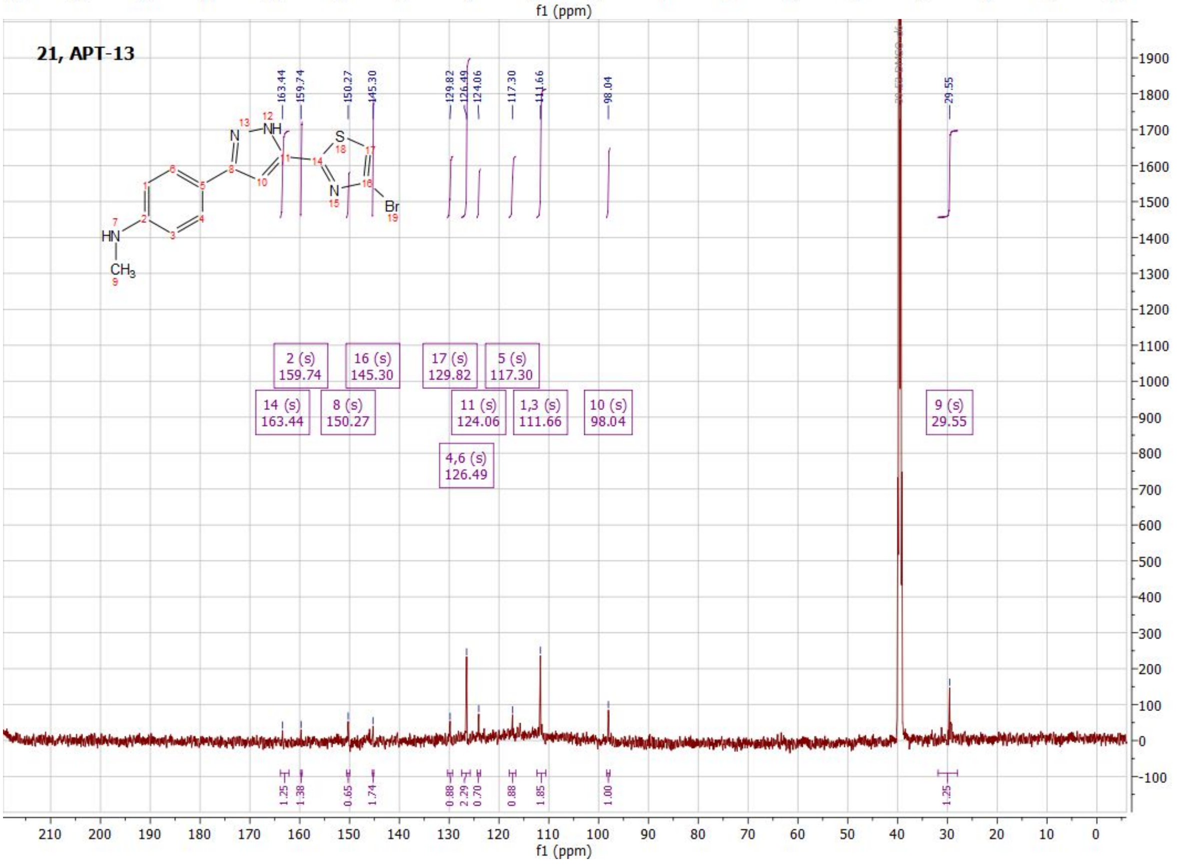

Figure S28. ^{1}H and ^{13}C NMR spectra of **21**.

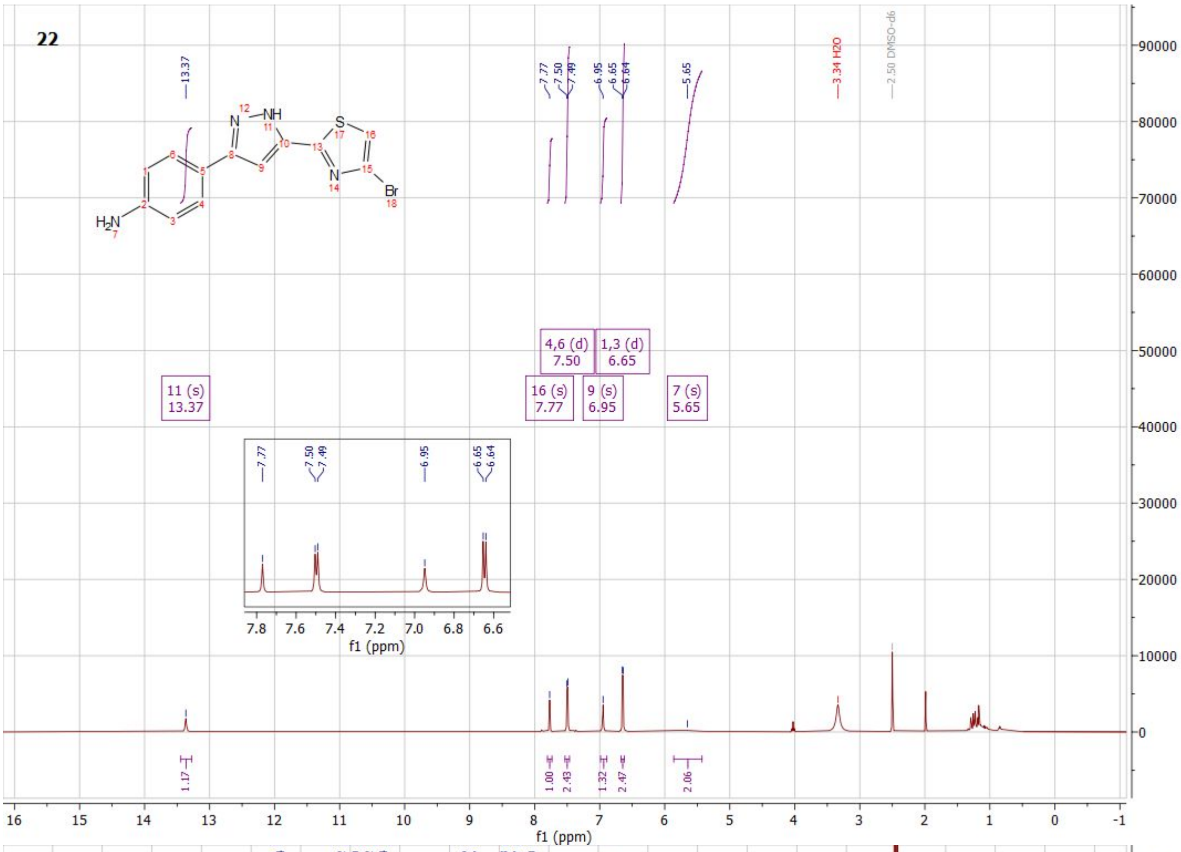

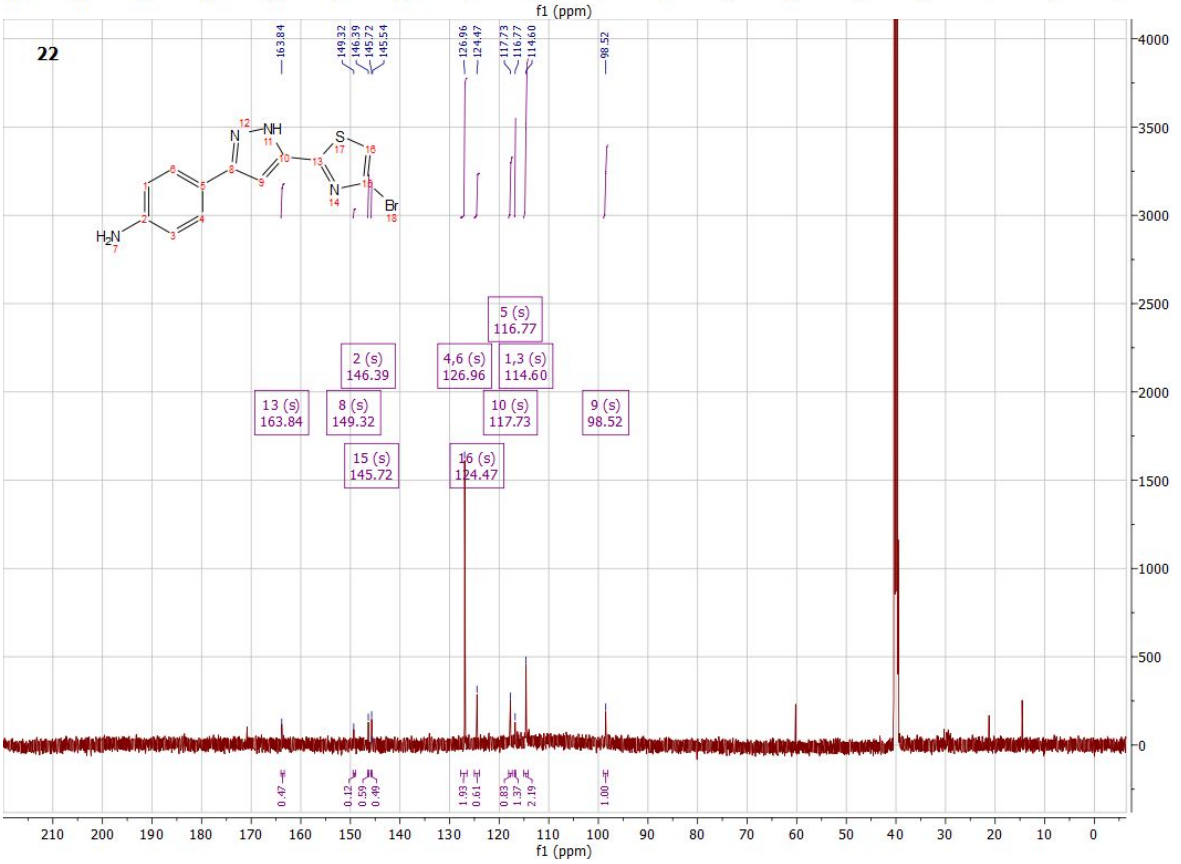

Figure S29. ^{1}H and ^{13}C NMR spectra of **22**.

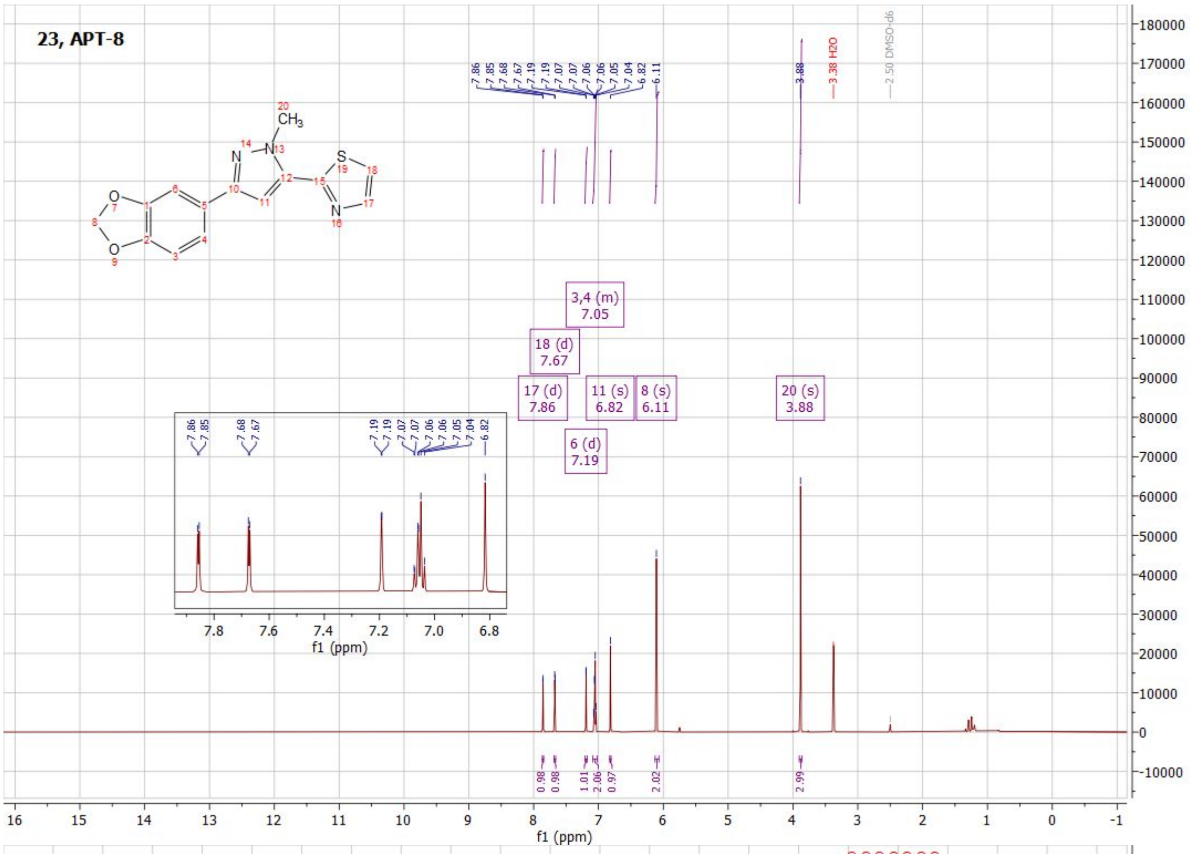

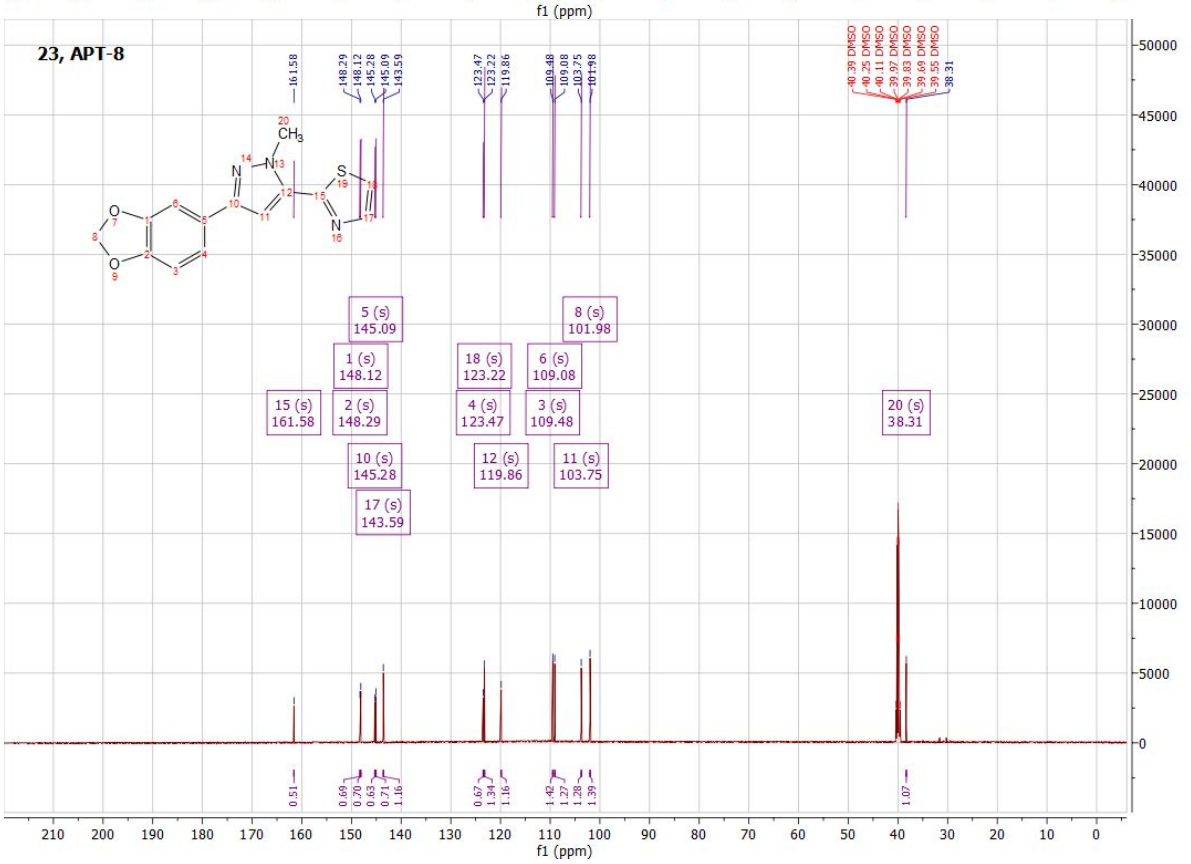

Figure S30. ^{1}H and ^{13}C NMR spectra of **23**.

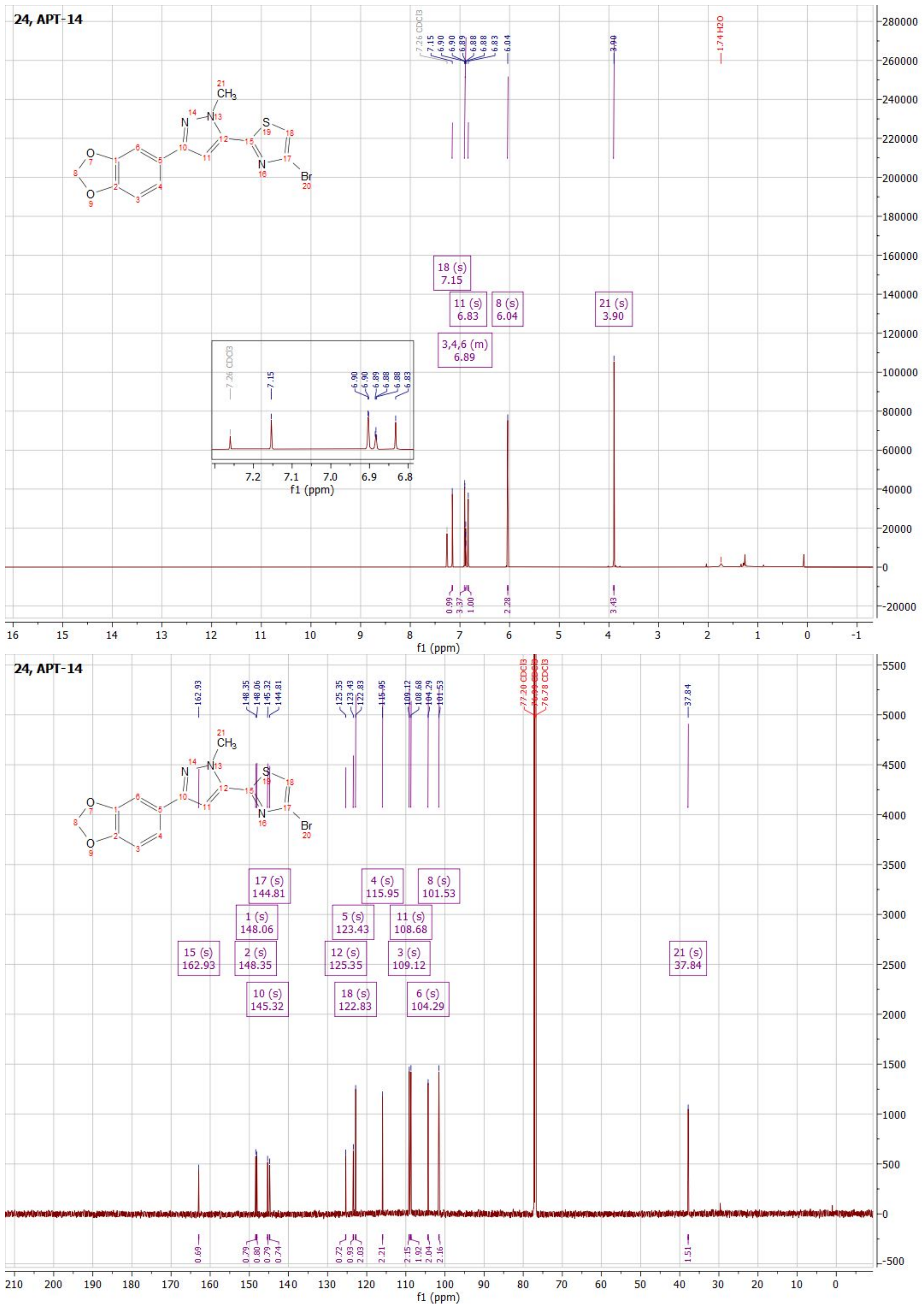

Figure S31. ^{1}H and ^{13}C NMR spectra of **24**.

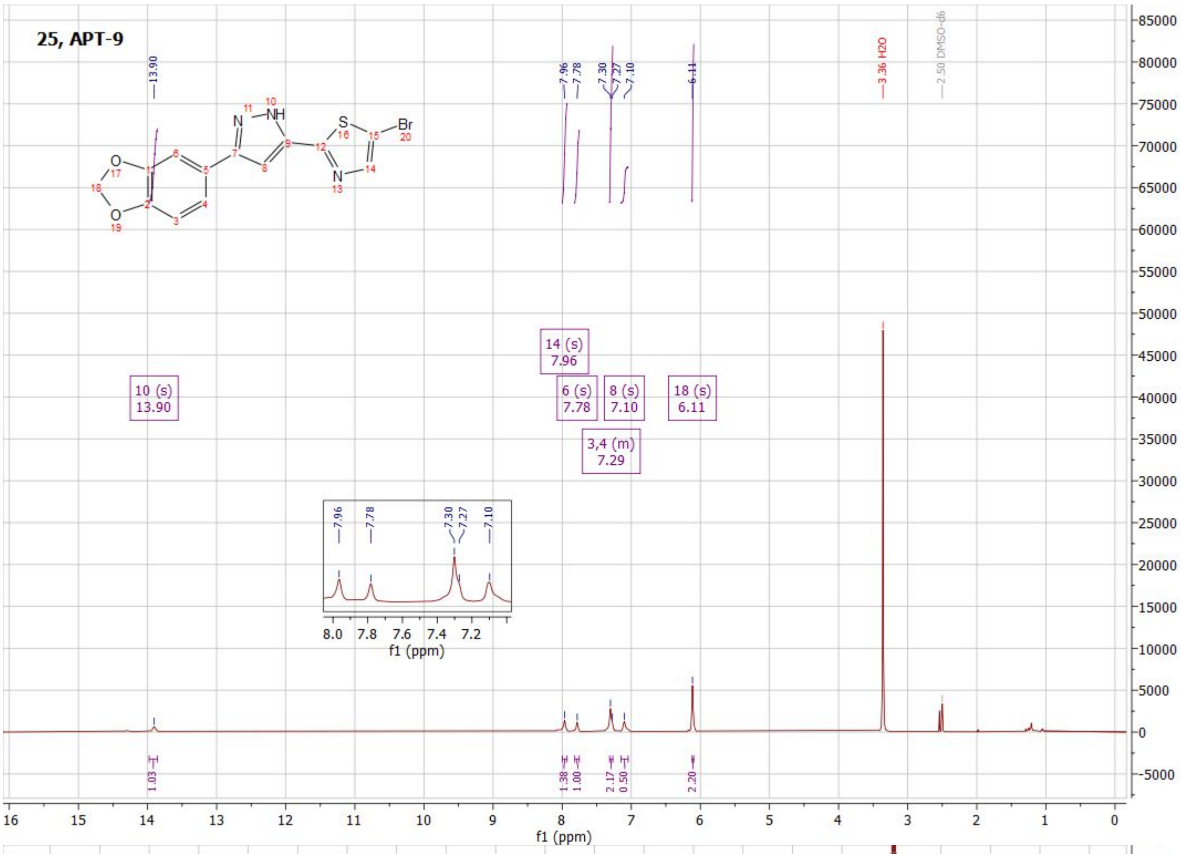

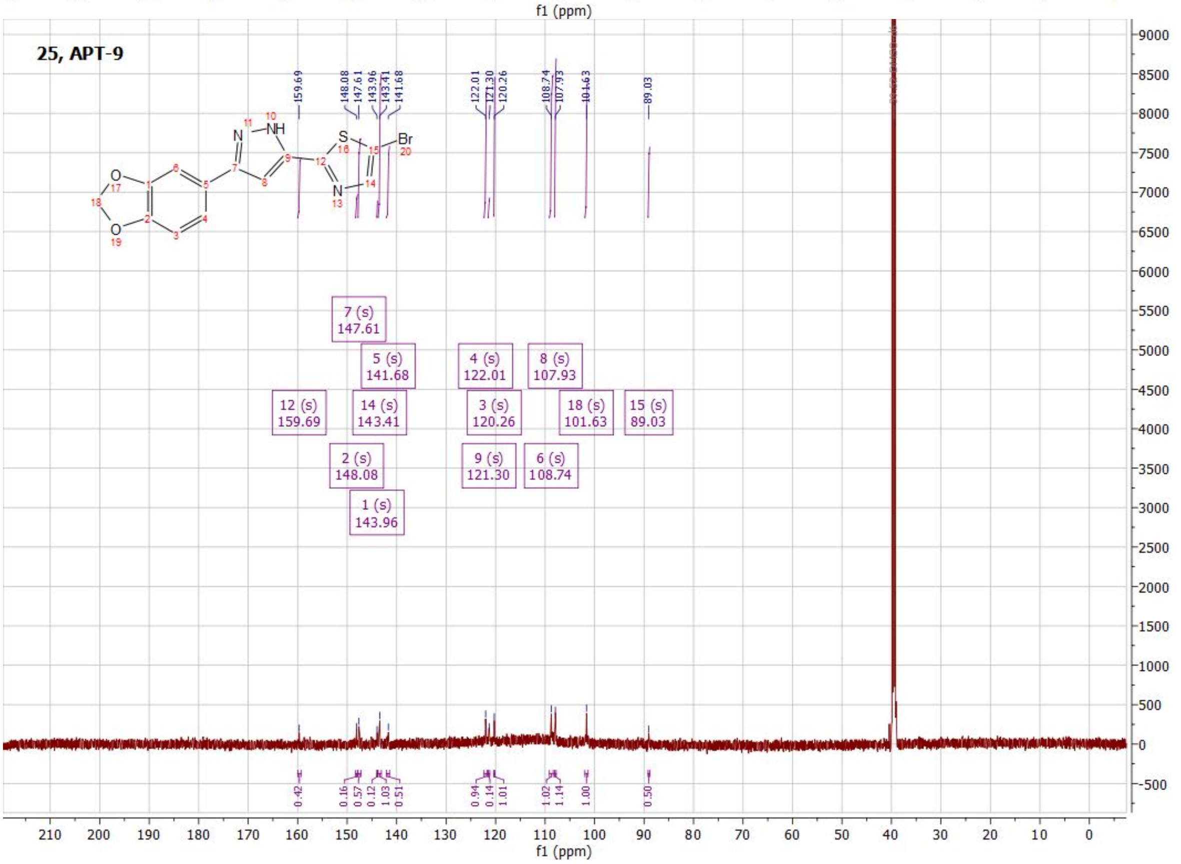

Figure S32. ^{1}H and ^{13}C NMR spectra of **25**.

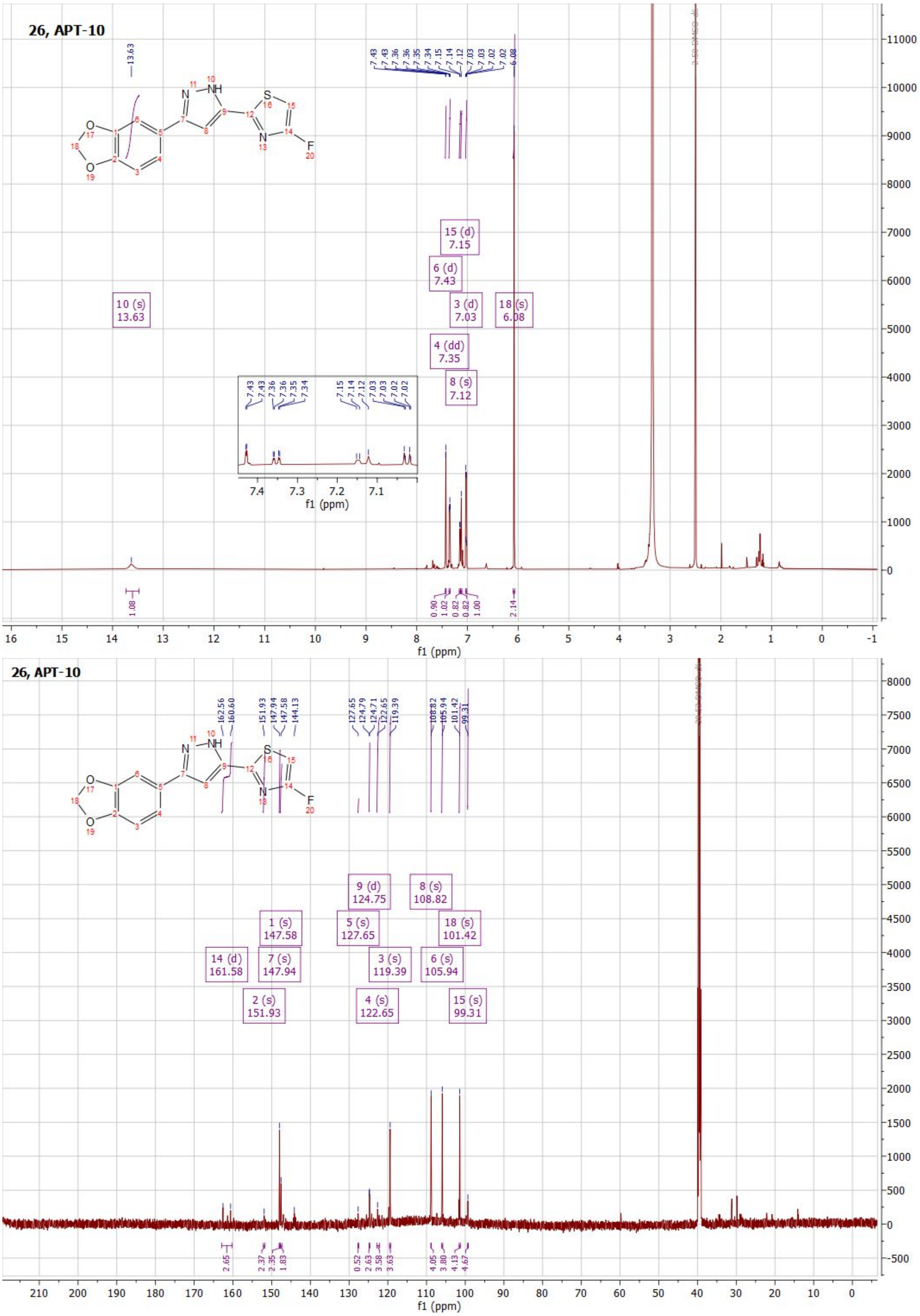

Figure S33. ^{1}H and ^{13}C NMR spectra of **26**.

The NMR spectra of compounds were analysed using MestreNova.

4. HPLC-MS chromatograms of APT-1-14

Chromatograms of APT-1-14 at Sig. 254 nm and mass spectrums correspondent to the main peak $[M+H]^+$.

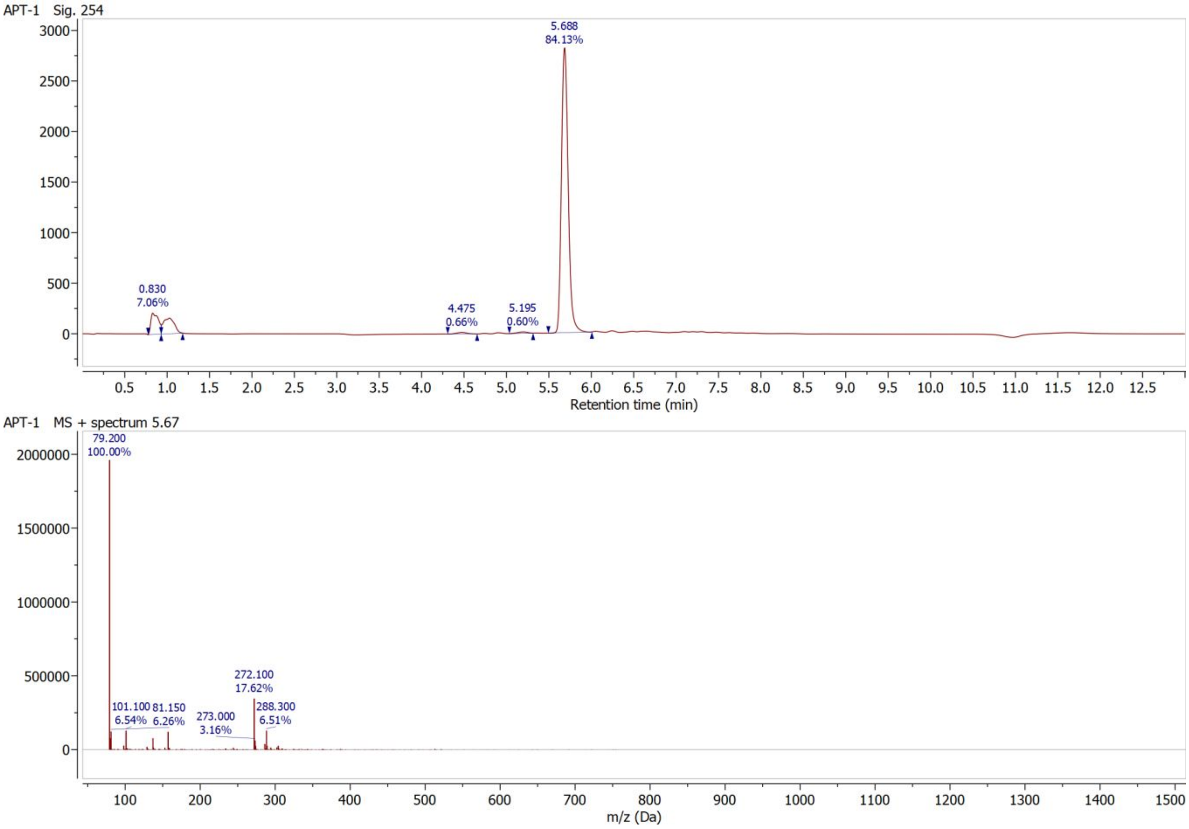

Figure S34. Chromatogram of APT-1 at Sig. 254 nm and mass spectrum of the main peak $[M+H]^+$.

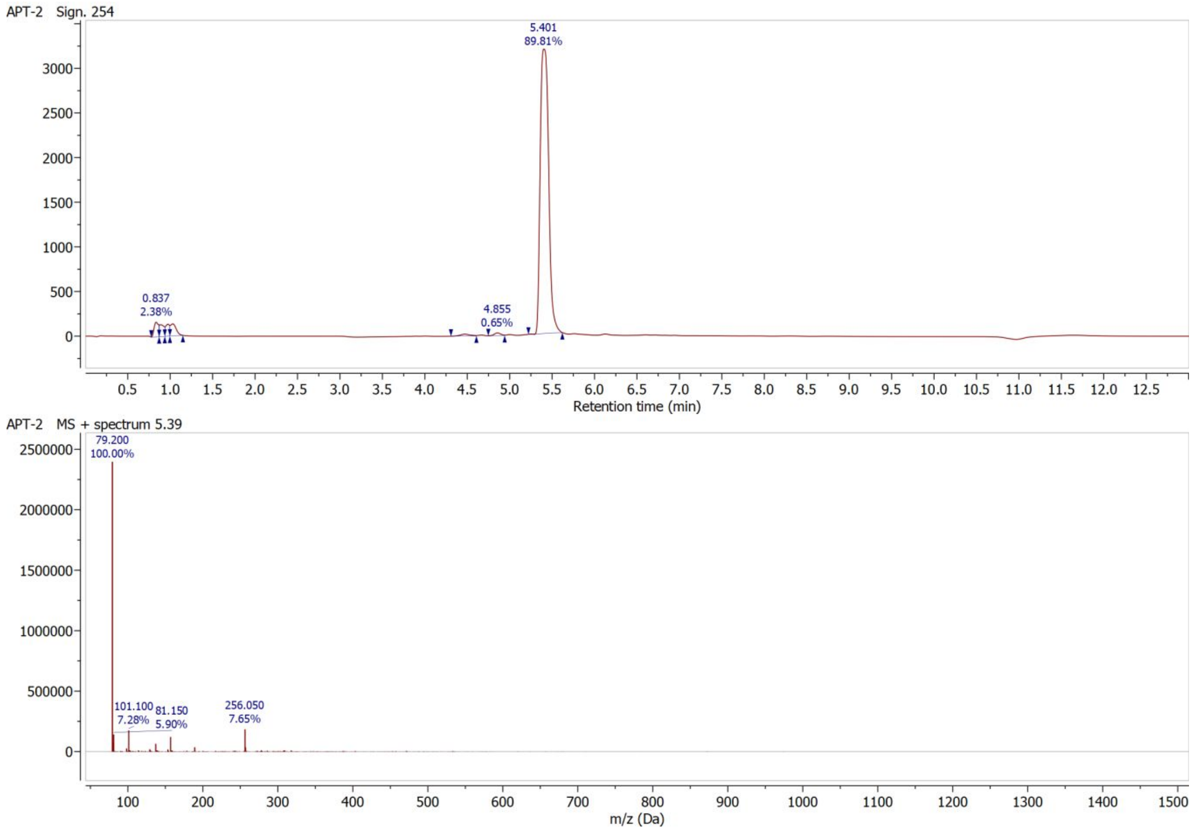

Figure S35. Chromatogram of APT-2 at Sig. 254 nm and mass spectrum of the main peak $[M+H]^+$.

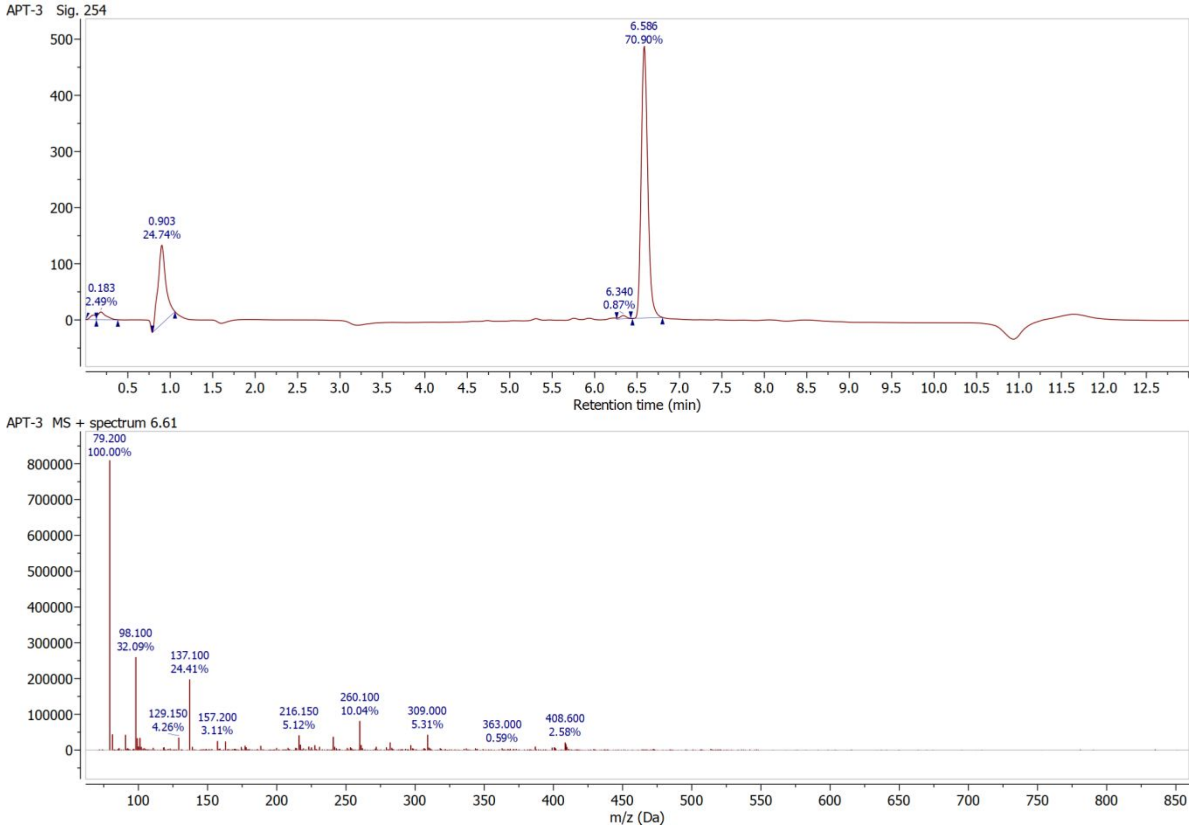

Figure S36. Chromatogram of APT-3 at Sig. 254 nm and mass spectrum of the main peak $[M+H]^+$.

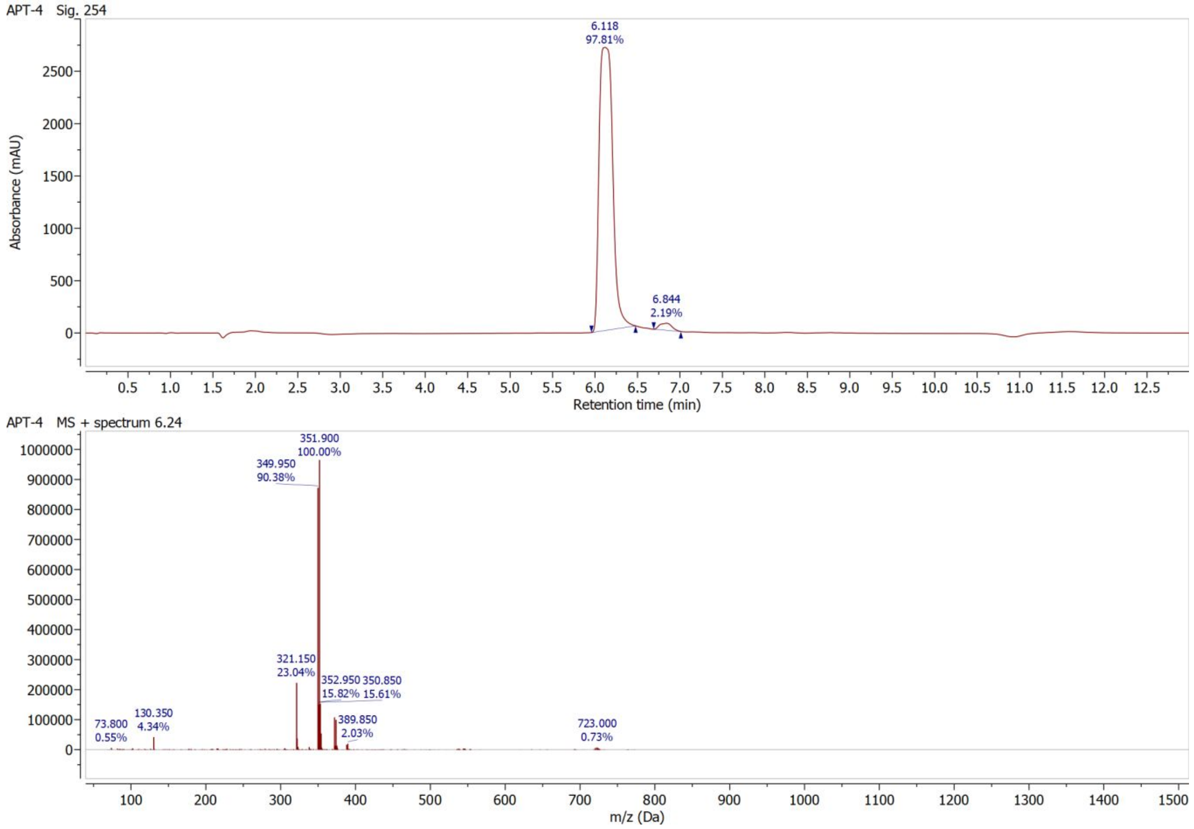

Figure S37. Chromatogram of APT-4 at Sig. 254 nm and mass spectrum of the main peak $[M+H]^+$.

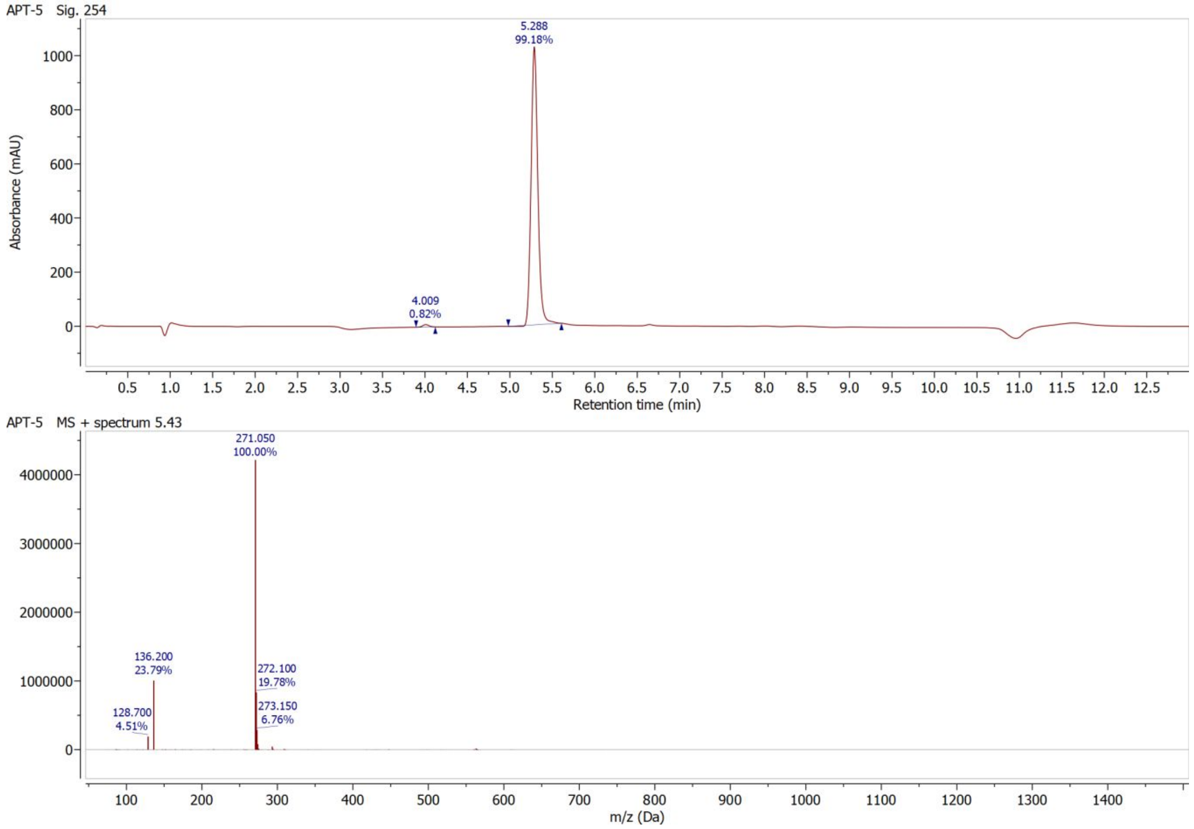

Figure S38. Chromatogram of APT-5 at Sig. 254 nm and mass spectrum of the main peak $[M+H]^+$.

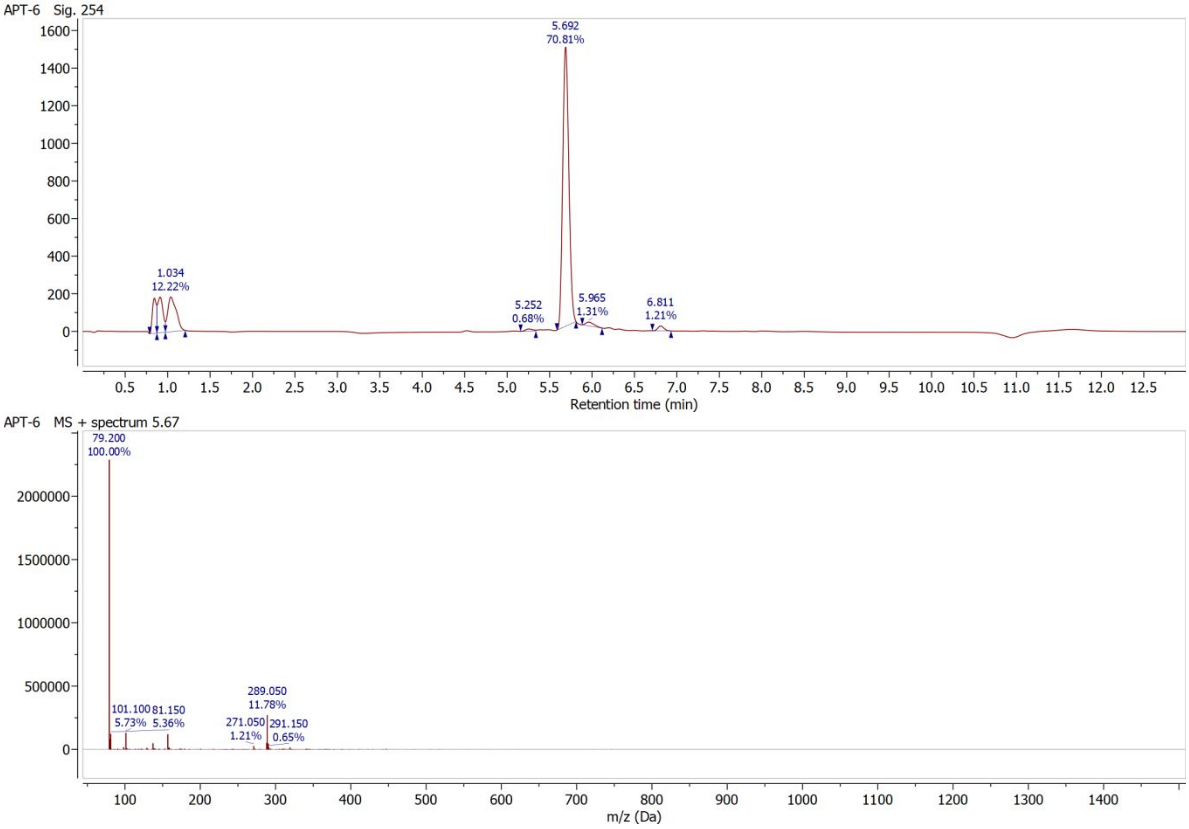

Figure S39. Chromatogram of APT-6 at Sig. 254 nm and mass spectrum of the main peak $[M+H]^+$.

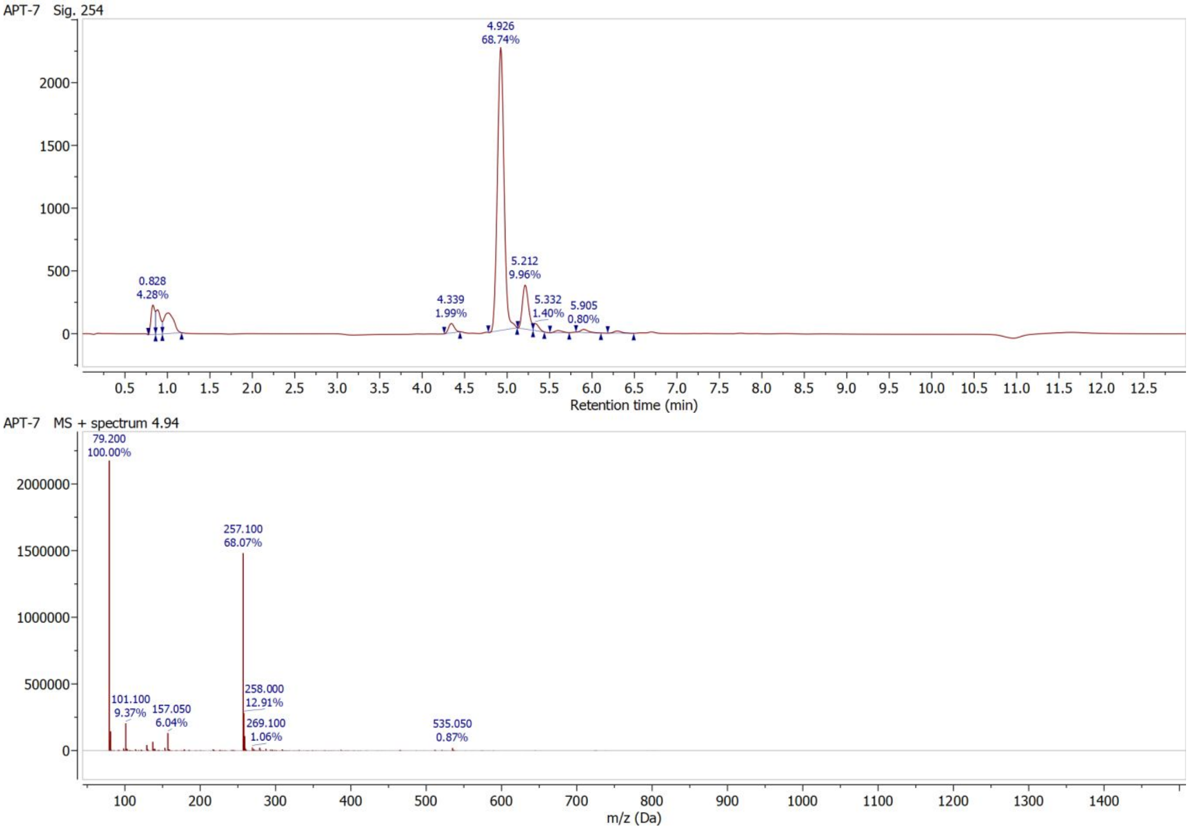

Figure S40. Chromatogram of APT-7 at Sig. 254 nm and mass spectrum of the main peak $[M+H]^+$.

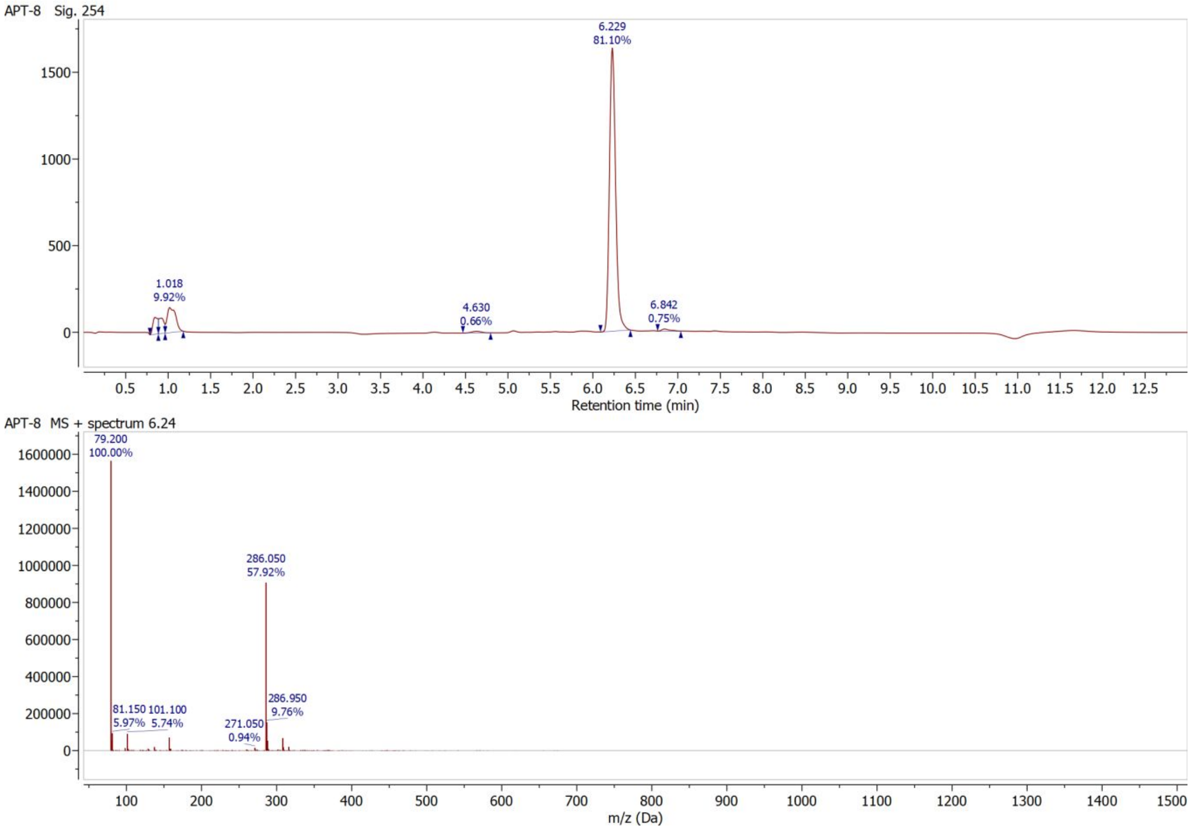

Figure S41. Chromatogram of APT-8 at Sig. 254 nm and mass spectrum of the main peak $[M+H]^+$.

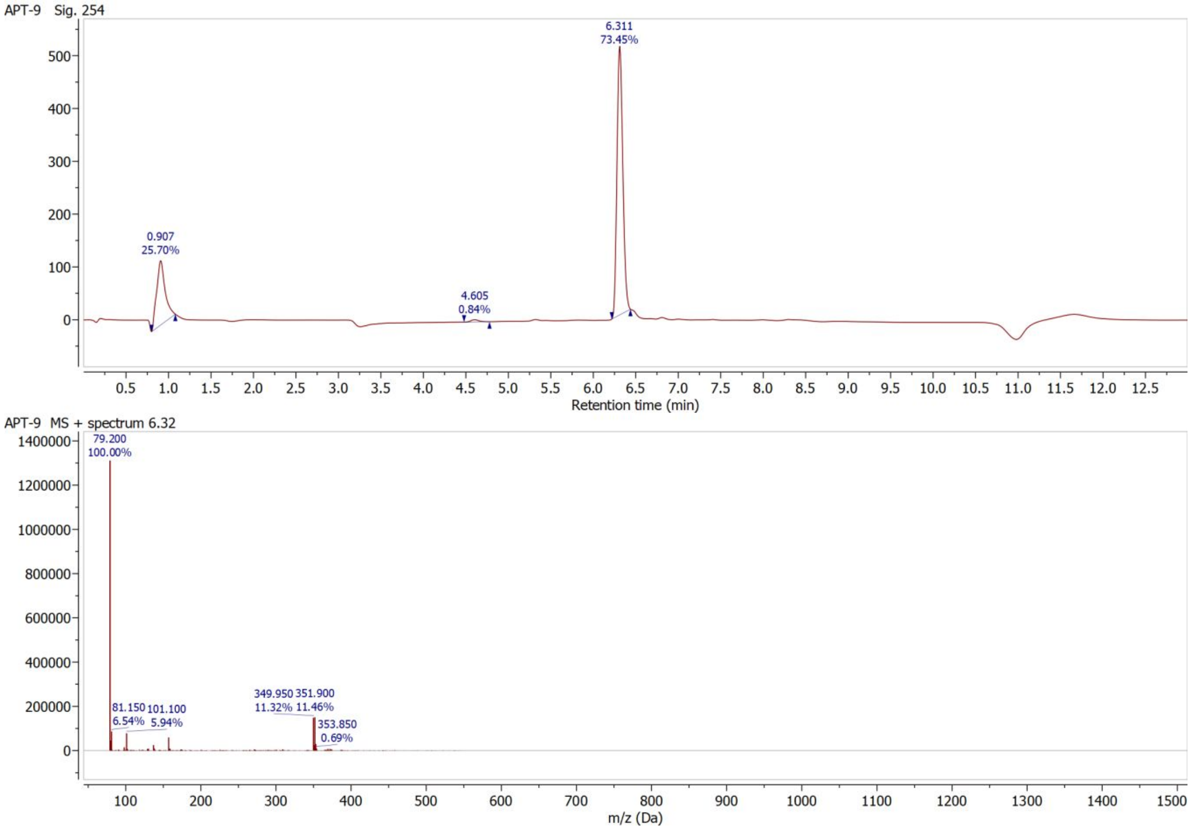

Figure S42. Chromatogram of APT-9 at Sig. 254 nm and mass spectrum of the main peak $[M+H]^+$.

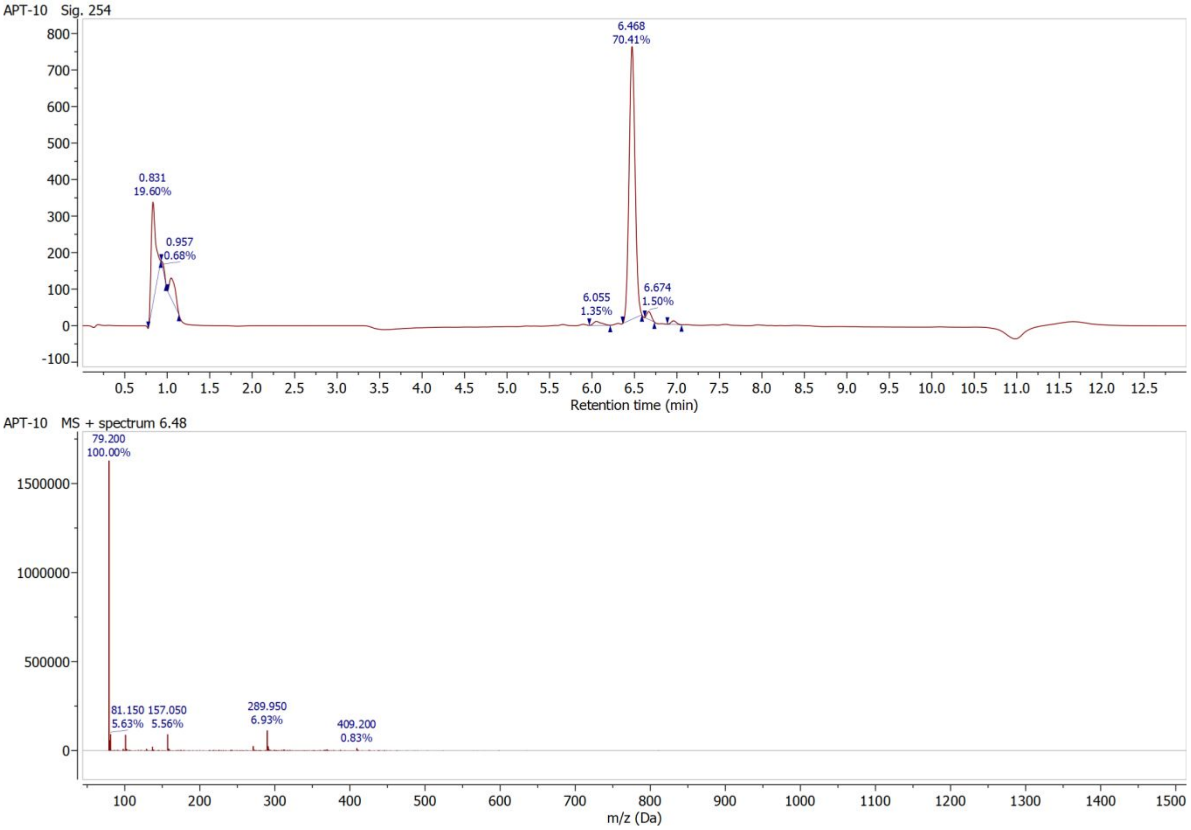

Figure S43. Chromatogram of APT-10 at Sig. 254 nm and mass spectrum of the main peak [M+H]⁺.

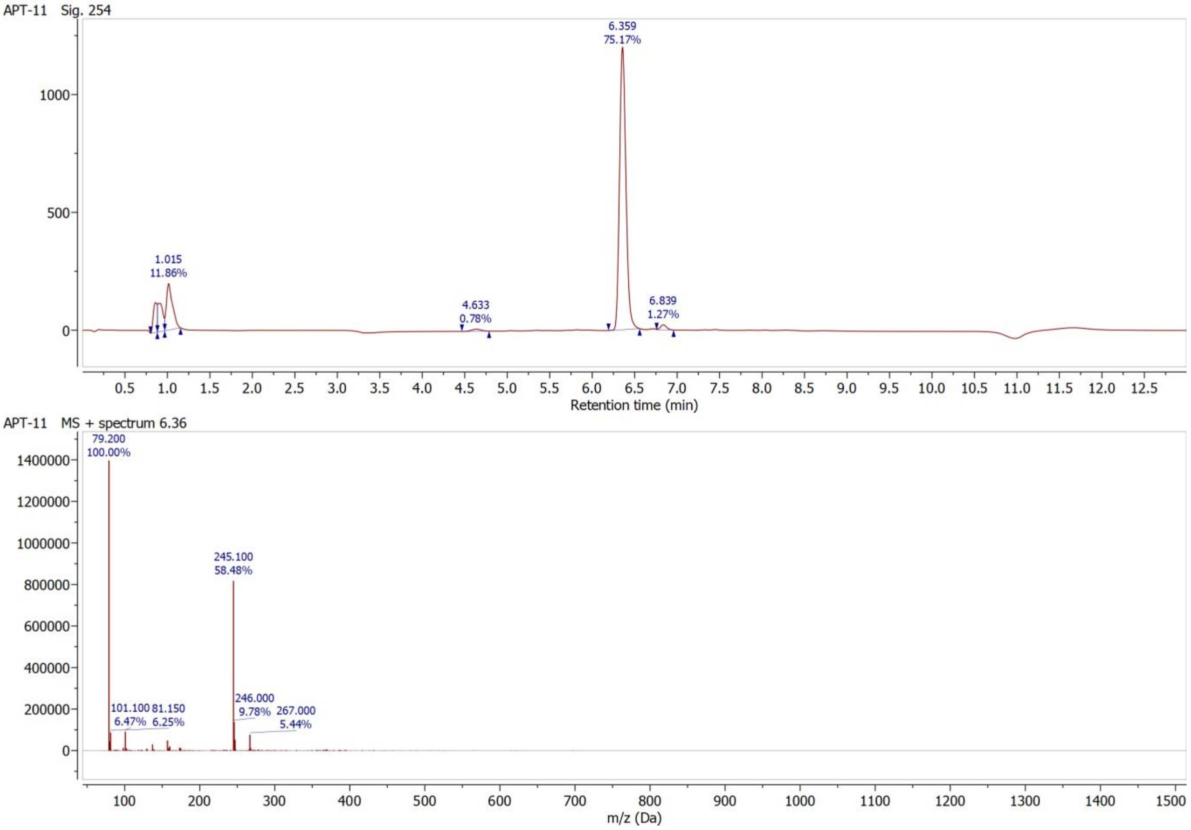

Figure S44. Chromatogram of APT-11 at Sig. 254 nm and mass spectrum of the main peak [M+H]⁺.

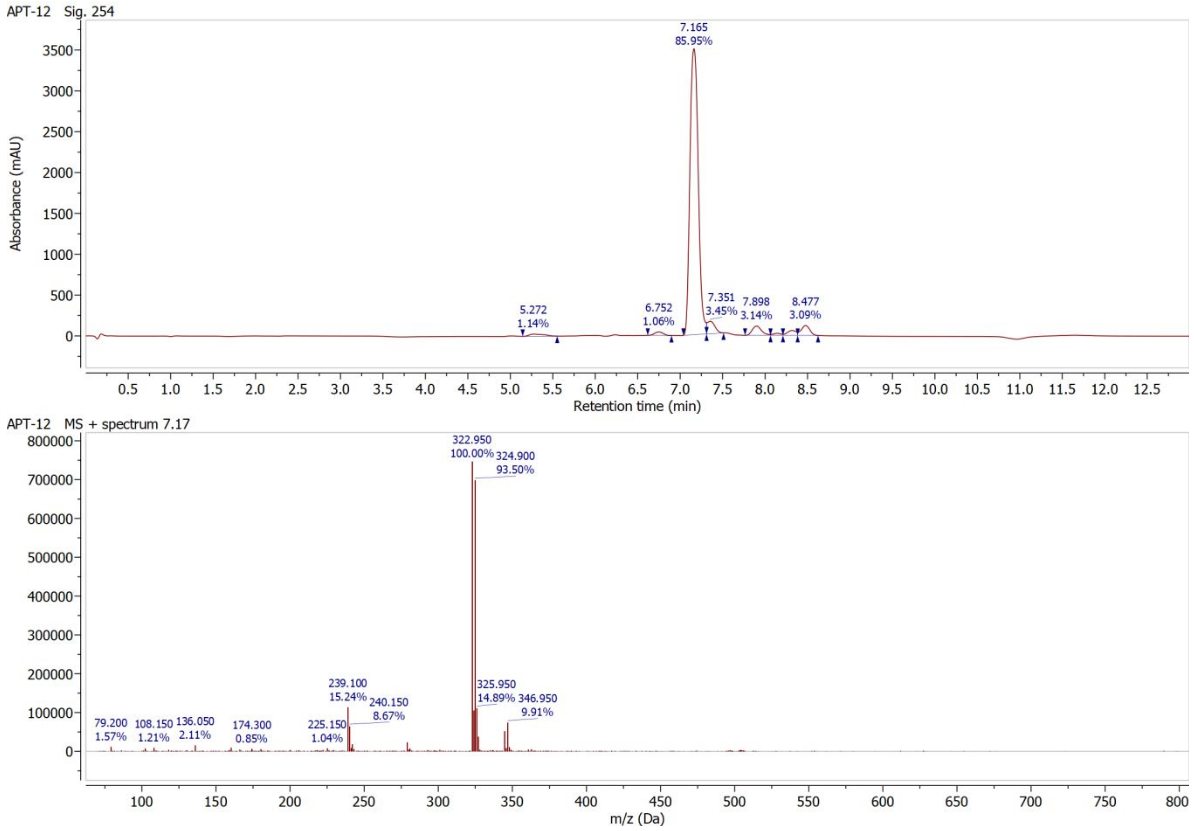

Figure S45. Chromatogram of APT-12 at Sig. 254 nm and mass spectrum of the main peak $[M+H]^+$.

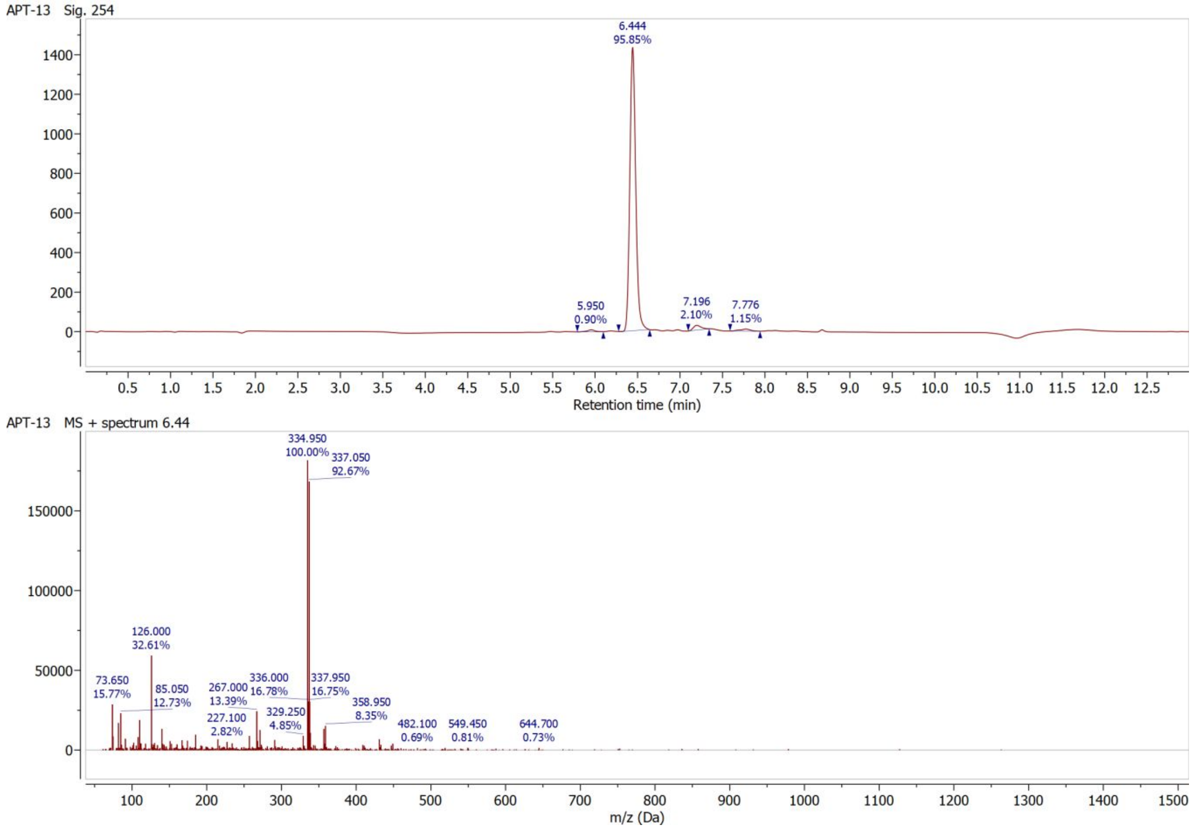

Figure S46. Chromatogram of APT-13 at Sig. 254 nm and mass spectrum of the main peak $[M+H]^+$.

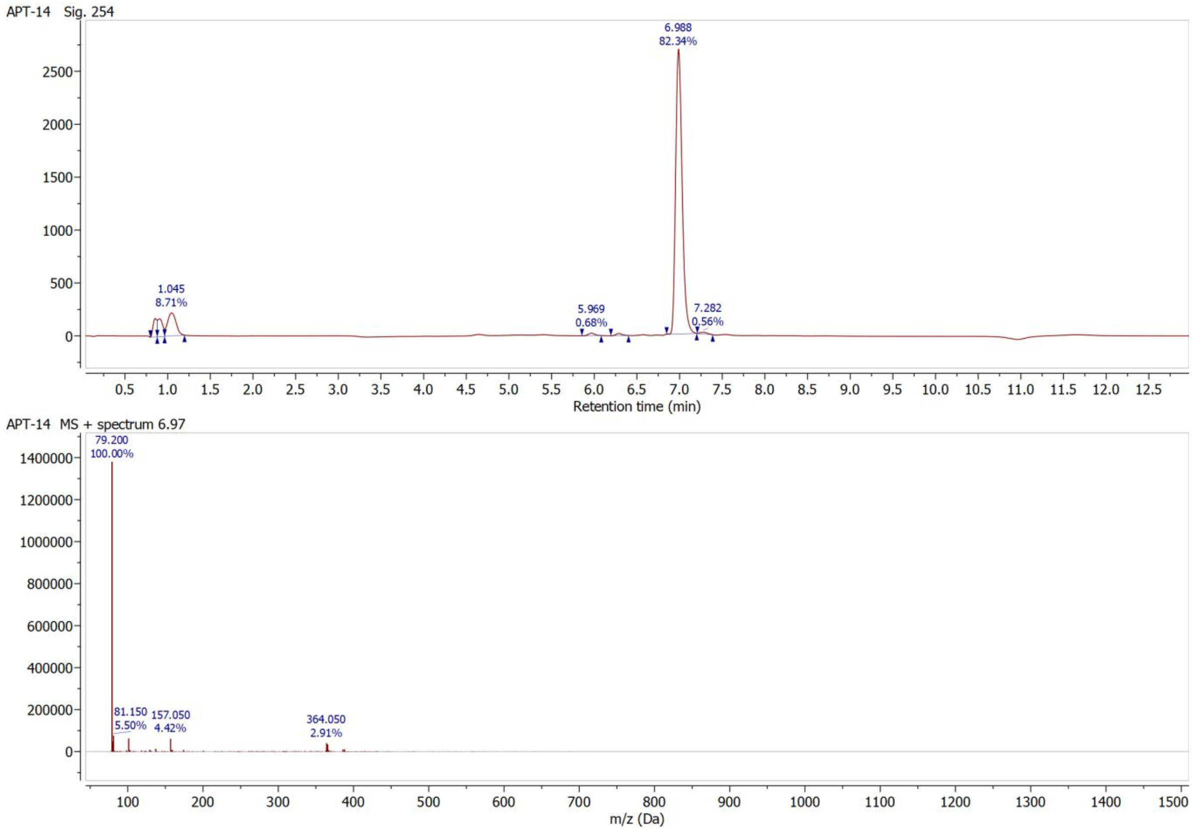

Figure S47. Chromatogram of APT-14 at Sig. 254 nm and mass spectrum of the main peak $[M+H]^+$.

REFERENCES

Di Nanni, A., R. S. Saw, U. M. Battisti, G. D. Bowden, A. Boeckermann, K. Bjerregaard-Andersen, B. J. Pichler, K. Herfert, M. M. Herth and A. Maurer (2023). "A Fluorescent Probe as a Lead Compound for a Selective α-Synuclein PET Tracer: Development of a Library of 2-Styrylbenzothiazoles and Biological Evaluation of [18F]PFSB and [18F]MFSB." ACS Omega **8**(34): 31450-31467.
